# A new Aura virus isolate in Brazil shows segment duplication in the variable region of the nsP3 gene

**DOI:** 10.1186/s13071-018-2907-4

**Published:** 2018-05-29

**Authors:** Ana Luiza Pamplona Mosimann, Mirian Krystel de Siqueira, Ligia Fernanda Ceole, Claudia Nunes Duarte dos Santos

**Affiliations:** 1 Laboratory of Molecular Virology, Instituto Carlos Chagas, FIOCRUZ, Rua Prof. Algacyr Munhoz Mader 3775, Cidade Industrial, Curitiba, PR 81350-010 Brazil; 2 Present Address: Department of Genetics, Evolution and Bioagents, Institute of Biology, University of Campinas, Campinas, SP 13083-970 Brazil; 3 Laboratory of Cell Biology, Instituto Carlos Chagas, FIOCRUZ, Rua Prof. Algacyr Munhoz Mader 3775, Cidade Industrial, Curitiba, PR 81350-010 Brazil

**Keywords:** Aura virus, nsP3, Duplication

## Abstract

**Background:**

A new isolate of Aura virus serendipitously discovered as a cell culture contaminant is reported in this manuscript. Aura virus belongs to the family *Togaviridae* and is classified in the genus *Alphavirus*. There are only two reports of Aura virus isolation from mosquitoes in the scientific literature, and the existence of a vertebrate host is still unknown. The discovery of this new isolate was based on transmission electron microscopy and nucleic acid amplification through a non-specific RT-PCR amplification protocol followed by sequencing.

**Results:**

Genetic analysis has shown that the new virus shares a high degree of identity with the previously described isolate (GenBank: AF126284.1). A major difference was observed in the nsP3 gene in which a 234-nucleotide duplication has been identified. Furthermore, a pronounced difference was observed in cell cultures compared to the data available for the previously described isolate. Cell permissiveness and phenotypic characteristics in C6/36, Vero and BHK-21 cells were found to differ from previous reports. This may be due to the genetic differences that have been observed.

**Conclusions:**

The genetic and biological characteristics of the new Aura virus isolate are suggestive of viral adaptation to the cell substrate. The development of a cDNA clone will lend a perspective and better understanding of these results as well as open avenues for its use as a biotechnological tool, as seen for other alphaviruses.

**Electronic supplementary material:**

The online version of this article (10.1186/s13071-018-2907-4) contains supplementary material, which is available to authorized users.

## Background

Aura virus (AURAV) is a member of the family *Togaviridae*, genus *Alphavirus*. Most alphaviruses are arthropod-borne viruses (arboviruses) that are involved in the etiology of human viral diseases whose main symptoms are rash, fever and arthralgia (Chikungunya virus, Mayaro virus, Ross River virus, and O’nyong-nyong virus) or encephalitis (Western equine encephalitis virus, Eastern equine encephalitis virus and Venezuelan equine encephalitis virus) [1]. Their genome consists of a positive sense single-stranded RNA of approximately 11.7 kb presenting two open reading frames with a cap at its 5' end and a poly-A tail at its 3' end [2].

The first isolations of AURAV were carried out in 1959, 1960 and 1961 by Causey et al. [3] from pools of *Culex* sp. and *Aedes serratus* mosquitoes that were collected in the vicinity of the city of Belém (Pará, Brazil). Some years later, this same virus was isolated from *Aedes serratus* collected in Misiones Province in Argentina [4]. As there are no other reports in the scientific literature of new isolations, the distribution is considered to be restricted to South America [5]. Despite being a virus that seems to be restricted to mosquitoes, it is not considered an insect-specific virus according to Bolling et al. [6]. It also does not possess a known vertebrate host; to date, it is considered non-pathogenic to humans [3]. Initial hemagglutination inhibition and complement fixation studies indicate that this virus is more closely related to Western equine encephalitis virus (WEEV) and Sindbis virus (SINV). However, serum neutralization studies indicate that it is quite antigenically different from these viruses [7, 8]. In later studies, the nucleotide sequencing of the prototype strain of AURAV (BeAR 10315) showed a higher genetic identity with SINV [9], and more recent phylogenetic studies of the genus *Alphavirus* have confirmed a closer genetic relationship with SINV and WEEV [5].

While working with a supernatant of the fifth passage (BR/P05) in an insect cell culture of a clinical sample in which dengue virus (DENV) type 3 had been previously identified, a phenotype that was not compatible with DENV infection was noticed. During infection kinetics (24, 48 and 72 h) in the Huh7.5 and C6/36 cells, the percentage of infected cells could not clearly be distinguished from the mock-infected cells when measured through flow cytometry using an anti-flavivirus monoclonal antibody (4G2). However, when the supernatants of these infection kinetics were titrated by plaque assay in C6/36 cell cultures, the titer of the supernatants from the C6/36 cell cultures increased over time, while almost no virus could be detected in the supernatant of Huh7.5 cell cultures. These results raised suspicion of the presence of a different virus in the BR/P05 sample.

## Results

**Fig. 1:**
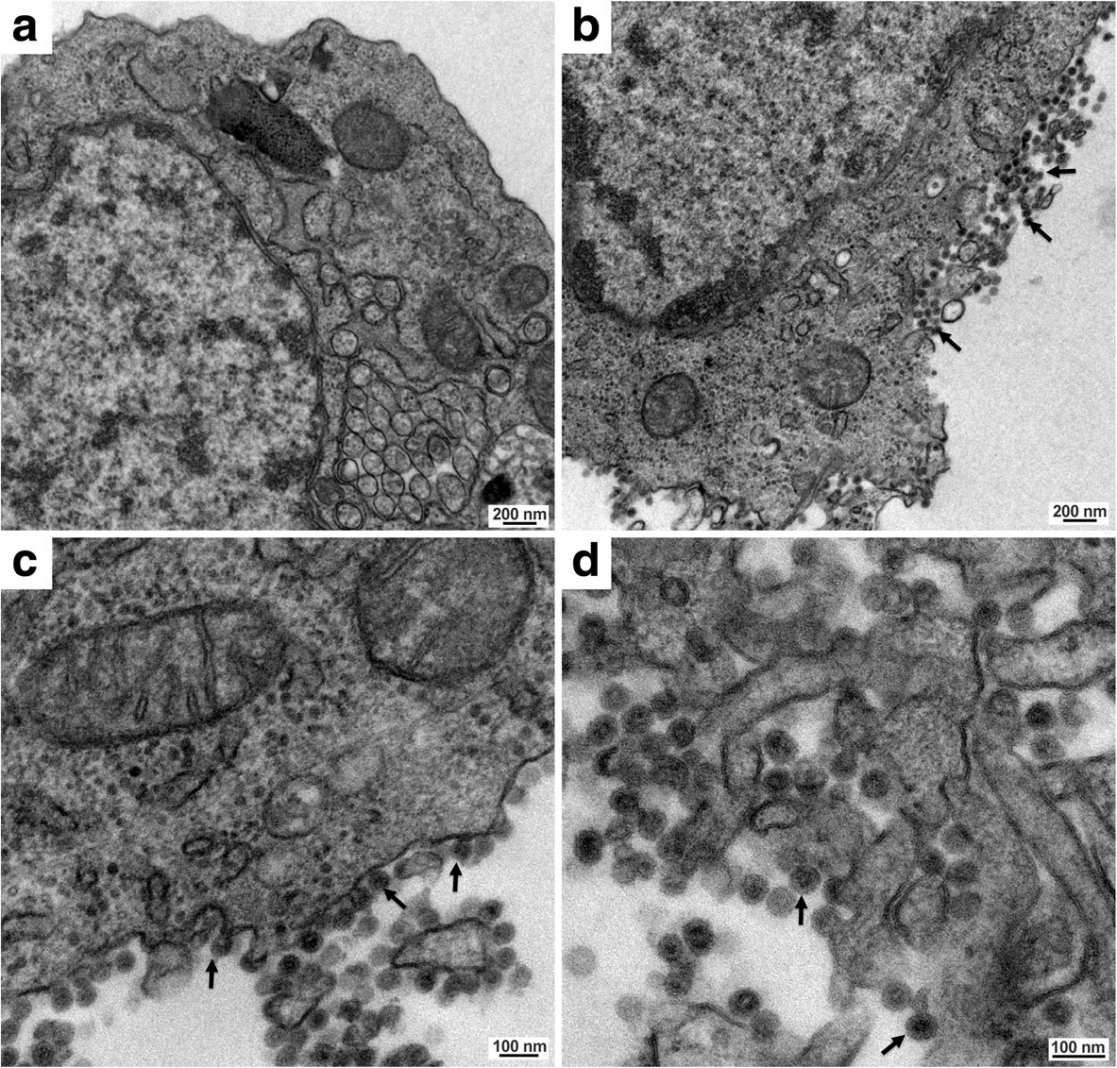
TEM of mock (**a**) and BR/P05 (**b-d**) C6/36 infected cells at 48 h post-infection. Arrows point to some of the virus particles that are budding or have just budded from the cell membrane. **b** through **d** represent progressively higher magnification fields of infected cells. *Scale-bars*: **a**, **b**, 200 nm; **c**, **d**, 100 nm

To address this question, we performed transmission electron microscopy (TEM) of C6/36 cells infected with BR/P05. As seen in Fig. 1, most of the identified viral particles were in close proximity to the cell, as if they had just budded from the cell membrane. This result was not compatible with TEM of DENV infection [10].

Next, we used an adapted non-specific RT-PCR amplification protocol that was designed to amplify any kind of viral nucleic acid. First, we carried out an enrichment step, which consisted of supernatant filtration through 0.22 μm, virion precipitation using polyethylene glycol 8000 and NaCl and DNase digestion to eliminate any cellular DNA. Next, total nucleic acids were extracted and either directly used in degenerate-oligonucleotide primed polymerase chain reaction (DOP-PCR), as previously described by Nanda et al. [11], or first subjected to reverse transcription using random primers. The amplified DNA was cloned into a TA vector and sequenced. The sequences presented homology with AURAV strain BeAr10315 (NC_003900.1 or AF126284.1), which is the only complete sequence of AURAV available in the GenBank database (Additional file 1: Table S1 and Additional file 1: Supplementary sequence data). In addition, the virions seen in the TEM presented an average size of 55.0 nm (Fig. 1) in accordance with a previous report on AURAV [12].

**Fig. 2:**
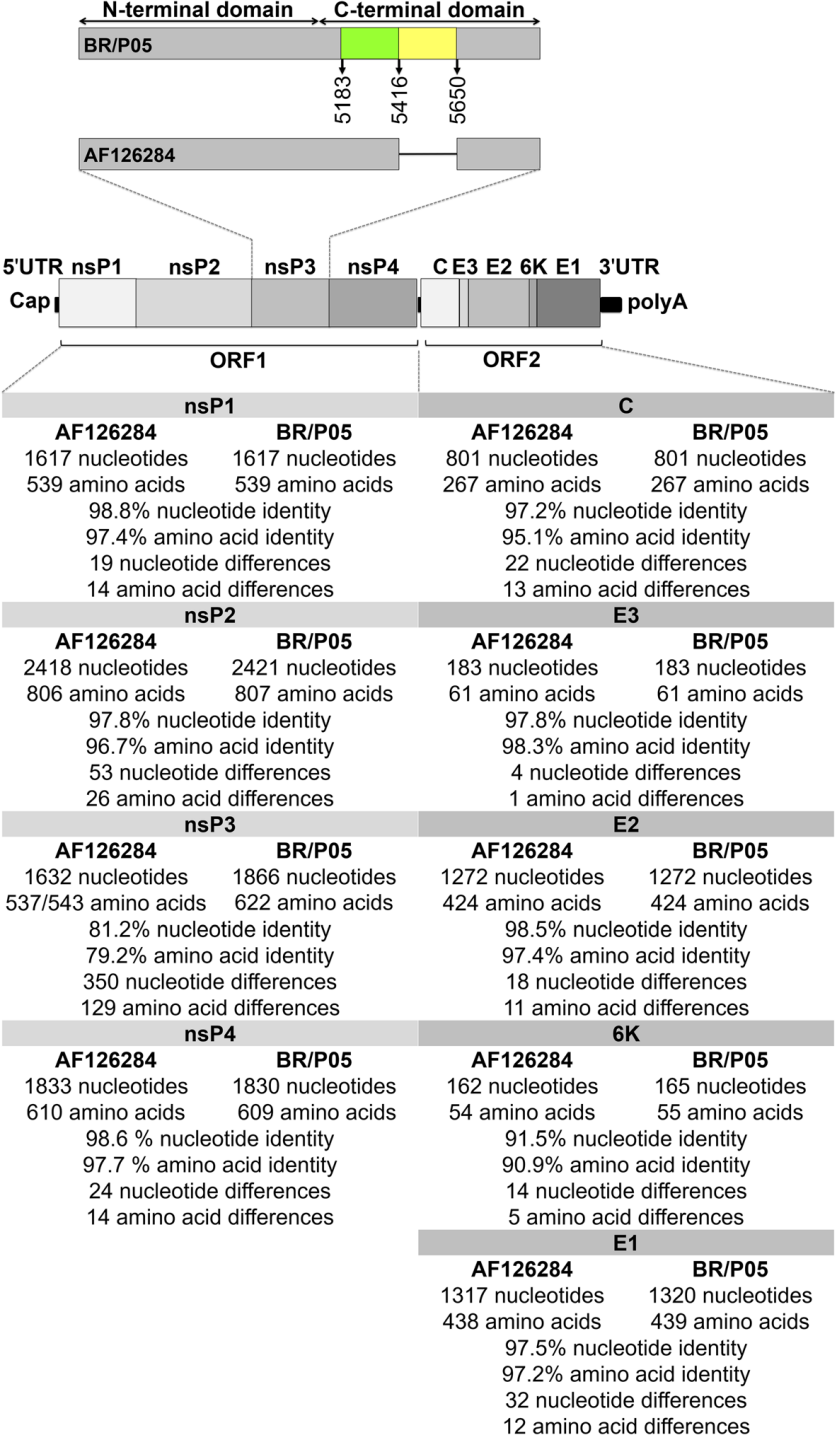
Comparison of the sequences of AF126284 and the new isolate of AURAV (BR/P05). A schematic representation of the *Alphavirus* genome is also shown. At the very top is an enhanced representation of the nsP3 gene that highlights the 234-nucleotide duplication that has been identified in BR/P05. The green and yellow boxes represent the duplicated sequence, and the black line in AF126284 represents the absence of the duplicated sequence in this genome

**Fig. 3:**
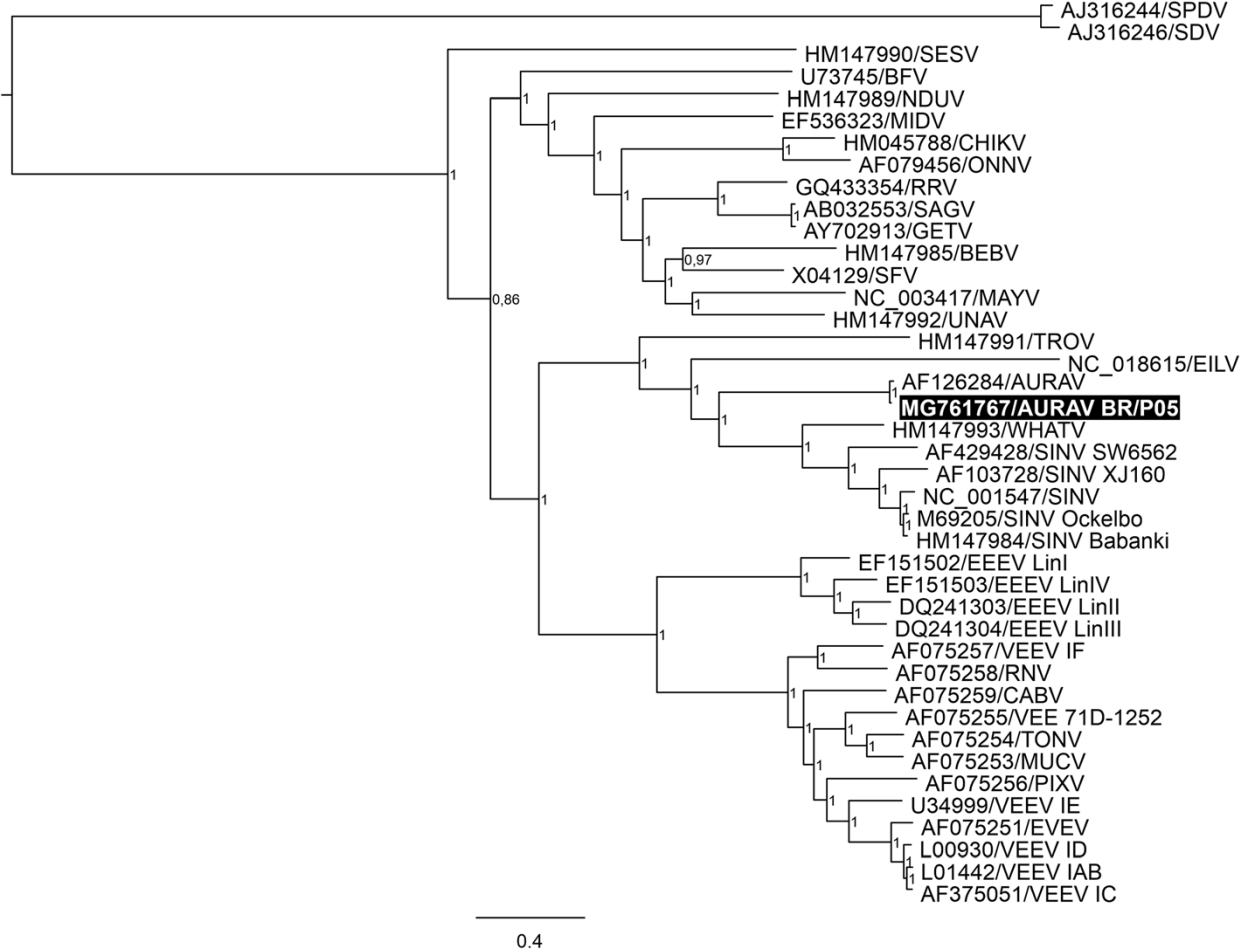
Phylogenetic analysis based on the alignment of the nucleotide sequences of the alphavirus concatenated ORFs. Segments of the nsP3 and C were excluded for not presenting reliable alignments. The tree was inferred using the MrBayes (v.3.2.6) software and is based on the general time reversible model with gamma-distributed rate variation and a proportion of invariable sites (GTR+I+G). The numbers shown to the right of the nodes represent posterior probabilities. Representatives from all species of alphaviruses have been included, except for the WEEV complex. The tree was midpoint rooted, and the sequence of the new isolate is highlighted in the black box (MG761767/AURAV BR/P05). Strains were labeled according to GenBank accession number/abbreviation, and the bar indicates nucleotide substitutions per site. Further details on the dataset used for phylogenetic analysis can be accessed in Additional file 1: Table S3

Once the virus had been identified, we proceeded with its full genetic characterization through genome sequencing. For that purpose, specific primers (Additional file 1: Table S2) were designed taking into account the genome sequence available in GenBank (AF126284.1) and the results obtained through the sequencing of the non-specifically amplified cloned RT-PCR fragments during the identification of this new isolate. The genome amplification strategy consisted of RT-PCR fragments covering the whole genome that possessed a minimal overlap required for full genome assembly. A comparison of the sequence of this new isolate (GenBank: MG761767) with the one previously described shows that they share significant nucleotide identity (95.4%) and deduced-amino acidic sequence (ORF1: 92.9% and ORF2: 96.6%). Detailed homology information for each gene is shown in Fig. 2. The specific polymorphic nucleotide and amino acid residues are shown in Additional file 1: Alignment, and its genetic relationship with other alphaviruses is depicted in Fig. 3. The most striking difference is seen in the sequence of the variable region of the nsP3 gene, which shows a 234-nucleotide duplication (highlighted in light yellow and light green in Additional file 1: Alignment and Fig. 2).

As mentioned, BR/P05 was first detected in the fifth passage of a DENV clinical sample in cell culture. To carry out the biological characterization experiments, different dilutions of the BR/P05 supernatant were incubated with the anti-flavivirus monoclonal antibody (mAb) 4G2, and this mixture was used to infect C6/36 cells. The goal was to neutralize any DENV particle that could still be present in the BR/P05 sample and hence have a homogeneous preparation of AURAV. The supernatant of the infection corresponding to the highest dilution of BR/P05 incubated with the anti-flavivirus antibody was used to prepare an AURAV stock for the cell culture experiments (BR/P07) (Additional file 1: Figure S1). It is important to emphasize that we could not detect any DENV in BR/P05 neither through one-step RT-PCR (Additional file 1: Figure S2a) nor through indirect immunofluorescence using mAb 4G2 (Additional file 1: Figure S2b).

**Fig. 4:**
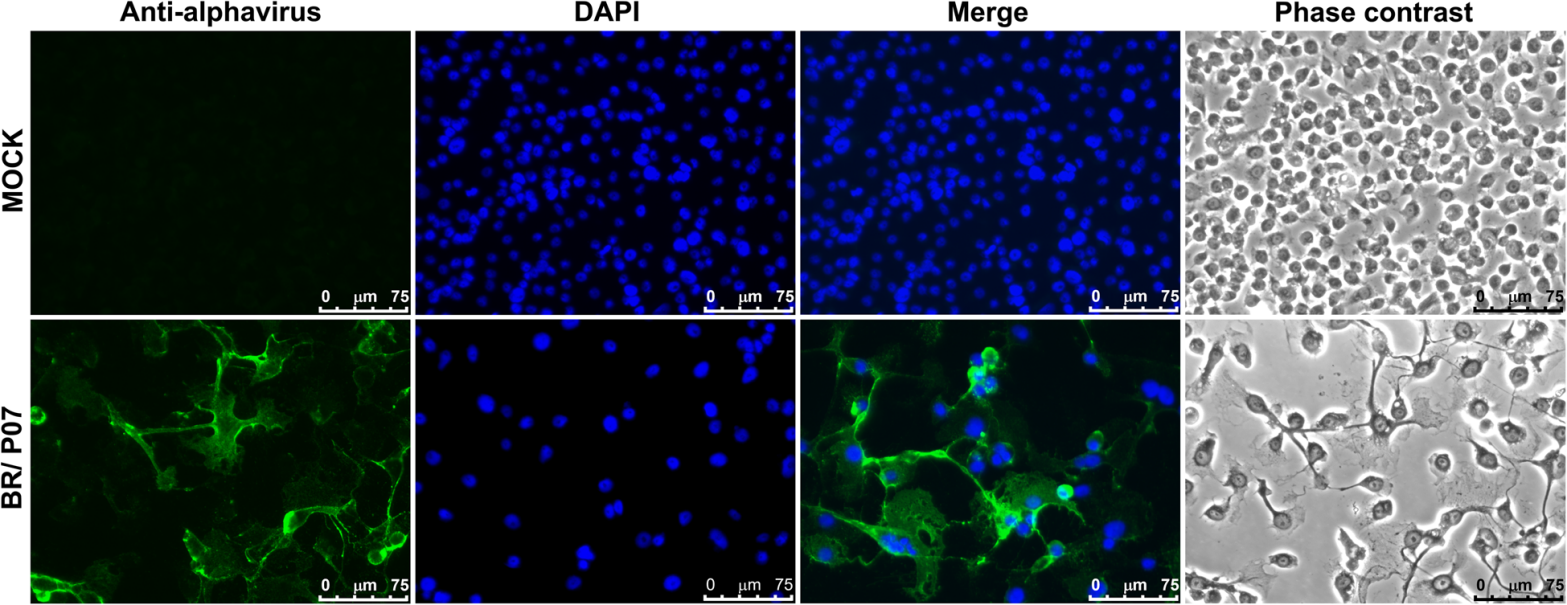
Indirect immunofluorescence assays of C6/36 cells infected or not (mock) with 1 MOI BR/P07. At 3 days post-infection, the cell monolayer was fixed and permeabilized with methanol:acetone (1:1) at -20 °C and then incubated with anti-alphavirus monoclonal antibody (mAb) clone 1A4B-6 followed by goat anti-mouse IgG (H+L) Alexa Fluor 488 conjugate. Pictures were taken using a Leica DMI 6000B inverted microscope attached to a Leica DFC365 FX camera, and the images were visualized and processed using the Leica Application Suite Advanced Fluorescence 3.1.0 software

AURAV stock titration was carried out in C6/36 cells (Additional file 1: Figure S3) through a plaque assay once this cell line exhibits a cytopathic effect (CPE) (Fig. 4 and Additional file 1: Figure S4). Infection could be confirmed through an indirect immunofluorescence assay (IFA) using anti-alphavirus mAb 1A4B-6 [13] (Fig. 4). The *Aedes pseudoscutellaris* (AP-61) mosquito cell line has also been shown to be permissive to viral infection (Additional file 1: Figure S5). Our attempts to titrate the present AURAV isolate in BHK-21 cells, either by plaque or focus immunodetection assays, failed, suggesting an insect-host specificity. We have also tried to detect infection in Vero and BHK-21 cells through IFA. Only when using very high MOIs (40 and 80 MOI for BHK-21 and 80 MOI for Vero) was it possible to visualize a few scattered positive cells (Additional file 1: Figure S6).

## Discussion

Rümenapf et al. [14] had previously shown that BHK-21 clone 15, Vero, primary chicken embryo fibroblasts (CEF) and C6/36 cells were permissive to AURAV infection. In their work, AURAV was titrated in BHK-21 cells through plaque assay [14]. On the other hand, Lascano et al. [12] carried out a study on the morphogenesis of Aura virus particles in CEF and the brains of newborn mice infected through intracerebral inoculation. Newborn mice intracerebral inoculation was also used for virus titration in this later case [12]. In addition to our results, no report of the CPE in mosquito cell lines after infection with AURAV has been found in the scientific literature. Notwithstanding, Garmashova et al. [15] have shown that nsP2 is associated with CPE development in the SINV model. In accordance with this finding, following nsP3, the gene that presented the highest number of non-synonymous mutations in BR/P05 was nsP2 (Fig. 2 and Additional file 1: Alignment).

The new AURAV isolate reported in this manuscript was first identified in the fifth passage in cell culture of a DENV-3 isolate. This sample was not isolated in our laboratory and was initially received as a cell culture supernatant (then named as P01). We have tested all previous passages (P01-P04) through RT-PCR for DENV and AURAV. Amplification was positive for both targets in all of these passages (Additional file 1: Figure S7). Mock infected C6/36 cells used as control, showed no amplification. This result indicates that the cell line used in our laboratory was not contaminated. Unfortunately, there is no information on the passage history of this sample previous to its arrival in our laboratory. As this virus is considered non-pathogenic to humans we can only hypothesize that the identification of AURAV in this sample was a result of cell culture contamination previous to its arrival in our laboratory; however, this is very difficult to track at this point.

Therefore it can be hypothesized that the genetic and biological differences observed in the present work are the result of a long period of interaction of this virus with the same cell substrate (possibly C6/36 cells), which resulted in a high adaptation to it. In favor of this theory, Weaver et al. [16] reported the substitution of the opal stop codon present in the C-terminus of the nsP3 gene by an arginine or cysteine after Eastern equine encephalitis virus (EEEV) adaptation to C6/36 cells. Furthermore, EEEV adaptation to C6/36 also resulted in a fitness loss for BHK-21 viral infection [16]. In AURAV BR/P05, the substitution of the opal stop codon with an arginine was also observed.

According to Bolling et al. [6] the only insect-specific alphavirus is Eilat virus (EILV). A recent study by Nasar et al. [17] investigated the host restriction of EILV. Results of this study suggest that EILV structural proteins do not mediate efficient attachment and entry into mammalian cells. Furthermore, EILV non-structural proteins are unable to sustain continued viral replication in these cells. Thus, EILV host-restriction was considered to depend on multiple genes. As observed for the AURAV new isolate (BR/P05), EILV can also be titrated through plaque assay in insect cells [18] suggesting this may be an insect-specific alphavirus feature. As previously reported by Nasar et al. [18] our analysis shows a close phylogenetic relationship between AURAV and EILV (Fig. 3). In spite of this, they constitute different species making it difficult to identify potential insect-specific amino acid motifs.

The role of non-structural protein 3 (nsP3) of alphavirus is still not fully elucidated. Studies using nsP3 mutants showed it to be required in the viral RNA synthesis [19]. It has also been shown to co-localize with other non-structural proteins to sites of viral RNA replication [20]. These data are also supported by co-immunoprecipitation experiments [20]. Although the N-terminal portion of the protein is conserved among different alphaviruses, its C-terminal portion is not [2]. Three domains have been identified in nsP3: the macro domain, the alphavirus unique domain (AUD) and the hypervariable domain (HVD) [19]. The macro domain is located in the N-terminal portion and is evolutionarily conserved across different Phylos [19]. The AUD is also a conserved element, albeit only among alphaviruses, and is located downstream of the macro domain in the central part of nsP3 [19]. On the other hand, the HVD, which is located in the C-terminal portion of nsP3, tolerates significant changes in sequence [19]. Nevertheless, the detection of conserved elements among the isolates of the same alphavirus species points to the evolutionarily advantageous characteristic of these sequences [19]. HVD sequences have also been shown to have an impact on the formation of distinct virus-specific protein complexes [21].

The scientific literature indicates that the segment duplication observed in the variable region of the nsP3 gene could play a role in the adaptation of AURAV to different hosts. Foy et al. [22] demonstrated that the phosphorylation of the HVD of nsP3 is more critical for the growth of Venezuelan equine encephalitis virus in insect cells (C7/10) than in vertebrate cell lines (BHK-21 and NIH 3T3). They have also shown that the permissiveness to different cell lines is selectively affected by the substitution of the nsP3 HVD by a heterologous protein-encoding sequence [22]. In addition, Neuvonen et al. [23] have identified SH3-binding motifs in the C-terminal portion of SFV, SINV and Chikungunya virus (CHIKV) nsP3. These SH3-binding motifs specifically interact with the host cell proteins amphiphysin 1 and amphiphysin 2, and this interaction has been shown to play a role in viral replication. The 234-nucleotide duplication observed in AURAV BR/P05 also results in the duplication of an SH3-binding motif (PVPPPR). As discussed by Neuvonen et al. [23], the insect amphiphysin gene is very similar to the mammalian genes and encodes a homologous SH3-binding motif whose interaction with SFV, SINV and CHIKV nsP3 has also been observed. The duplication also resulted in the presence of the DILVQAEVH motif in triplicate (underlined in red in Additional file 1: Alignment), whose significance is unknown. In addition, a difference in the hydrophobicity plot can be detected in the region of duplication, which presents non-synonymous substitutions (Additional file 1: Figure S8). This difference may influence nsP3 interaction with membranes and/or other hydrophobic residues of other molecules [24].

Speculating about the origin of the observed duplication, it has been noticed that when the sequence of AURAV AF126284 is aligned against itself using the BLAST 2 sequences algorithm, the expected results of 100% identity were observed. In addition, in the genomic region where the duplication has been identified in the new isolate, there is also a segment (5238–5417, in green) which presents high identity (82%) with another neighboring segment (5415–5594, in blue) (Additional file 1: Figure S9a). This could have resulted in homologous recombination [25] or replication error, which may have originated this duplication. During the synthesis of the negative strand, when the replication complex reaches position 5238, the two strands of the replicative intermediate may temporarily detach, or the replication complex may switch strands. Then, the recently copied upstream segment (5238–5417, in green) in the negative strand may hybridize to the neighboring downstream segment (5415–5594, in blue) in the positive strand and resume the synthesis of the negative strand, resulting in the duplication of the upstream segment (dashed green line, see Additional file 1: Figure S9b for further details). However, as this genomic segment, which presents partial identity, has a smaller size (181 nucleotides) than the observed duplication (234 nucleotides), other additional events would have been necessary to account for all the observed differences.

## Conclusions

In summary, we report the finding of a new isolate of AURAV. It is difficult to track its origin, but its genetic and biological characteristics are suggestive of viral adaptation to the cell substrate. To better understand the impact of the observed genetic differences on the biological phenotype, an infectious cDNA clone is needed. In addition to contributing to unraveling some of the questions raised in this manuscript (i.e. What is the role of the nsP3 segment duplication in the virus adaptation to the insect cell line? Are the nsP2 mutations related to CPE development?), it can become a valuable molecular tool as already described for other alphaviruses [26, 27].

## Methods

### Isolate identification

#### Transmission electron microscopy

For transmission electron microscopy, C6/36 cells were infected or not (mock) with BR/P05 for 48 h and fixed with 2.5% glutaraldehyde in 0.1 M sodium cacodylate buffer for 1 h. Cells were washed twice with 0.1 M cacodylate buffer, pH 7.2, and subsequently fixed in 1% OsO_4_, 0.8% KFe (CN)_6_ and 5 mM CaCl_2_ diluted in 0.1 M cacodylate buffer for 1 h. After fixation, the cells were washed, dehydrated in increasing concentrations of acetone and embedded in Poly/Bed 812 resin for 72 h at 60 °C. Ultrathin sections were stained for 30 min with uranyl acetate and for 2 min with lead citrate before analysis in a JEOL JEM-1400 transmission electron microscope (JEOL, Tokyo, Japan) at 80 kV [28]. For average size estimation, 152 virions were manually measured based on the size scale for the mean calculation.

#### Non-specific RT-PCR nucleic acid amplification

The supernatant (10 ml) of a three-day post-infection insect cell culture was centrifuged at 3220× *g* for 30 min at 4 °C to pull-down any cell debris. The supernatant of this centrifugation was then filtered through a 0.22 μm sterile filter [29]. The filtered supernatant (10 ml) was precipitated with 1.4 g of polyethylene glycol 8000 and 0.47 g of NaCl and incubated overnight at 4 °C under gentle agitation. Then, it was centrifuged at 3200× *g* for 30 min at 4 °C. The supernatant was discarded, the pellet was resuspended with 0.5 ml of DPBS containing Ca^++^ and Mg^++^ (Lonza, Walkersville, MD, USA) [30] and incubated with 100 U of Turbo DNAse I (Ambion, Austin, TX, USA) at 37 °C for 2 h. The viral particle lysis was carried out through incubation with 10% (v/v) 10% SDS and 1% (v/v) 14.3 M 2-mercaptoethanol (Sigma-Aldrich, St. Louis, MO, USA) at 72 °C for 3 min. Total nucleic acids were extracted through the addition of an equal volume of phenol:chlorophorm:isoamyl alcohol (25:24:1), mixing and centrifugation at 20238× *g* for 2 min at room temperature (RT). The aqueous phase was then mixed with an equal volume of chloprophorm:isoamyl alcohol (24:1) and centrifuged at 20238× *g* for 2 min at RT. The aqueous phase was mixed with 2.5 volumes of ethanol and 0.8 M LiCl (Ambion) and incubated at -20 °C for approximately 64 h. Subsequently, this solution was centrifuged at 20817× *g* for 15 min at 4 °C, the supernatant was discarded and 0.3 ml of 70% ethanol was added to the pellet. Finally, this mixture was vortexed and centrifuged at 20817× *g* for 7 min at 4 °C, the supernatant discarded, and the pellet dried at RT and resuspended with 50 μl of H_2_O. All reagents cited in this section were nuclease free. A non-infected cell culture (mock) supernatant was subjected to the same procedure as a negative control.

The total nucleic acid extracted as described above was used as a template in reverse transcription, using the ImProm-II Reverse Transcriptase (Promega, Madison, WI, USA) with random primers (Invitrogen, Carlsbad, CA, USA), followed by PCR or directly used as a template in the PCR reaction. Reverse transcription was carried out according to the manufacturer’s instructions. The PCR reaction was performed in 20 mM Tris-HCl (pH 8.4), 50 mM KCl, 1.5 mM MgCl_2_, 200 μM dNTPs, 0.06 U/μl Taq DNA polymerase (IBMP, Curitiba, Brazil) and 1.2 μM DOP Primer (Additional file 1: Table S2), as previously described by Nanda et al. [11]. The following cycling conditions were applied: one cycle of 95 °C for 5 min, 5 cycles of 94 °C for 1 min, 30 °C for 1.5 min, ramping to 72 °C at 0.2 °C/s, and 72 °C for 3 min, followed by 35 cycles of 94 °C for 1 min, 55 °C for 1 min, and 72 °C for 2 min, with the addition of 14 s/cycle to the extension step [11]. The amplified DNA was purified using the High Pure PCR Product Purification Kit (Roche, Mannheim, Germany) following the protocol for the purification of PCR products in solution after amplification, cloned in the pGEM-T-easy vector (Promega) and used to transform *Escherichia coli* Top10F’ cells. Plasmid DNA was purified from twenty selected white colonies through miniprep using the Wizard Plus SV Minipreps DNA Purification System (Promega), and the presence of an insert was confirmed through NotI (New England Biolabs, Ipswich, MA, USA) digestion. The concentration of the purified plasmid DNAs was measured using a Nanodrop ND-1000 (Thermo Fisher Scientific, Wilmington, DE, USA), and then sent to Macrogen (Seoul, Korea), where they were sequenced using an Applied Biosystems 3730xl DNA Analyzer (Applied Biosystems, Foster City, IA, USA).

### Genetic characterization

RNA was extracted from the supernatant of BR/P05 using the QIAamp Viral RNA Mini Kit (Qiagen, Valencia, CA, USA) according to the manufacturer’s instructions. This RNA was amplified through reverse transcription using the ImProm-II Reverse Transcriptase and random primers (Invitrogen) followed by PCR using the Qiagen LongRange PCR System (Qiagen) according to the manufacturer’s instructions. Specific primers (Additional file 1: Table S2) were designed taking into account only the full-length genome sequence available in GenBank (NC_003900.1 or AF126284.1) and the results obtained through the sequencing of the non-specifically cloned and amplified RT-PCR fragments during the identification of this new isolate. The RT-PCR amplified fragments were purified using either the High Pure PCR Product Purification Kit or the QIAquick Gel Extraction Kit (Qiagen). The concentration of the purified DNA fragments was measured using a Nanodrop ND-1000, and then sent to Macrogen, where they were sequenced using an Applied Biosystems 3730xl DNA Analyzer.

To sequence the 5' and 3' ends of the AURAV that were identified in BR/P05, RNA extracted from the supernatant of the infected cell cultures was first decapped through incubation with tobacco acid pyrophosphatase (Epicentre, Madison, WI, USA) at 37 °C for 1 h. The decapped RNA was purified by phenol extraction as described in the “Nonspecific RT-PCR nucleic acid amplification” section, but instead of 0.8 M of LiCl, 10% (v/v) 3 M sodium acetate, pH 5.3, was used in the precipitation step. This decapped RNA was ligated through incubation with T4 RNA ligase (New England Biolabs, Ipswich, MA, USA) at 37 °C for 30 min followed by 16 h at 16 °C. The ligated product was purified by phenol extraction as described above and used as the template for an RT-PCR reaction with the AURAV23F and AURAV2R primers. The PCR product was purified with a High Pure PCR Product Purification Kit and either directly sequenced or inserted into the pGEM-T-easy vector for nucleotide sequencing.

The nucleotide sequences were assembled using the phred (version 0.020425.c), phrap (version 1.080812) and consed (version 17.0) software packages [31–34], and whenever needed, pairwise alignment was carried out using the Basic Local Alignment Search Tool (BLAST) [35]. The complete genome consensus sequence was submitted to the GenBank database under the accession number MG761767.

The dataset used in the phylogenetic analysis (Additional file 1: Table S3) was based on the dataset used by Nasar et al. [18]; however, we have excluded WEEV and WEEV-like and concatenated the two ORFs as done by Forrester et al. [36] when undertaking full-genome analysis. The sequences were aligned using the muscle algorithm [37] as implemented in MEGA (version 7.0.14), and the segments of the nsP3 and C that did not present reliable alignments, were excluded. A consensus tree was inferred using MrBayes (version 3.2.6 ×86). MrBayes analyses were carried under the general time reversible model with gamma-distributed rate variation and a proportion of invariable sites (GTR+I+G) using three hot chains and one cold chain and was run for 2 million generations with a 25% burn-in.

For the production of the Additional file 1: Alignment and Fig. 2, the alignment in the nsP3 genomic region was manually edited to clearly represent the duplication.

### Cell culture, viral stock and biological characterization

C6/36 (ATCC, CRL-1660) cells were maintained in Leibovitz’s L-15 medium (Gibco, Grand Island, NY, USA) supplemented with 5% fetal bovine serum (FBS; Gibco), 0.26% tryptose and 25 μg/ml of gentamicin (Gibco) at 28 °C. AP61 cells were maintained in Leibovitz’s L-15 medium supplemented with 10% FBS, 0.56% tryptose and 25 μg/ml of gentamicin at 28 °C. Vero (ATCC, CCL-81) and BHK-21 (ATCC, CCL10) cells were maintained in DMEM-F12 (Gibco) supplemented with 10% FBS, 100 U/ml of penicillin and 100 μg/ml of streptomycin (Sigma-Aldrich). All cells used in this work tested negative for mycoplasma contamination.

The RNA extracted from BR/P05 tested negative for dengue in a one-step RT-PCR protocol used for dengue virus serotyping [38]. However, to be sure that the supernatant that was going to be used in the biological characterization experiments was free from dengue virus serotype 3, different dilutions (10^-1^–10^-6^) of the supernatant of BR/P05 were incubated at 37 °C with anti-flavivirus monoclonal antibody (4G2) for 2 h. This mixture was incubated with C6/36 cells (3.5 × 10^5^ cells/well, seeded the day before in a 6-well plate) for 1 h at 28 °C. Then, the inoculum was discarded, the cell monolayer was washed once with a sterile PBS solution and 3 ml/well of medium added. Two days post-infection, the supernatants were collected, aliquoted and stored at -80 °C. The supernatant of the infection that was carried out with the least amount of virus was titrated and used to infect C6/36 cells at a multiplicity of infection (MOI) of 0.01 to produce the viral stock (BR/P07) for the biological characterization experiments (Additional file 1: Figure S1).

When needed, titration was carried out in C6/36 monolayers as follows. Twenty-four-well plates were seeded the day before with 1 × 10^5^ cells/well and infected with tenfold dilutions (in duplicate) of viral supernatants. Dilutions were made in the medium without FBS supplementation, and incubation was carried out at 28 °C for 1 h. After the incubation period, the inoculum was discarded, and the cells were overlaid with 500 μl of a 1:1 mixture of CMC 3,2% and Leibovitz’s L-15 medium supplemented with 10% FBS, 0.52% tryptose and 50 μg/ml of gentamicin. Plates were then sealed with tape and incubated for 7 days at 28 °C. At this point, the overlay was discarded, and cell monolayers were washed thrice with PBS, fixed with 3% paraformaldehyde in PBS at RT for 20 min and stained with a solution of 0.8% crystal violet (w/v), 0.5% NaCl (w/v) and 10% formamide (v/v) in ethanol. Plaques were counted in duplicate wells, and the mean was calculated. This value was divided by the volume of inoculum and multiplied by the dilution factor to obtain the result in PFU/ml.

The permissiveness of AP61, BHK-21 and Vero cells was tested. For this purpose, 48-well plates were seeded the day before with either 1 × 10^4^ cells/well (Vero and BHK-21) or 5 × 10^4^ cells/well (AP61). Cells were then infected (duplicate wells) with MOIs of 1, 10 and 40 in the case of AP-61 or 10, 40 and 80 in the case of Vero and BHK-21. Infection conditions were incubation at 28 °C for 1 h for AP61 and at 37 °C for 1 h for Vero and BHK-21 cells. After this incubation period, the cell monolayer was washed twice with a sterile PBS solution, and 500 μl/well of medium was added. Three days post-infection, the supernatants were collected, aliquoted, and stored at -80 °C, and the cells were fixed and permeabilized with a mixture of methanol:acetone (1:1) at -20 °C for at least 1 h. This time point was chosen based on the results of Rümenapf et al. [14]. Afterward, the cells were incubated at 37 °C for 1 h with anti-alphavirus monoclonal antibody 1A4B-6 (EMD Millipore, Temecula, CA, USA) diluted 1:300, washed three times with PBS, incubated at 37 °C for 1 h with goat anti-mouse IgG (H+L) cross-adsorbed secondary antibody, Alexa fluor 488 (Life Technologies, Eugene, OR, USA) diluted 1:100 and washed again three times with PBS. Finally, the cells were overlaid with 100 μl/well of PBS containing 10% glycerol and observed in a Leica DMI 6000B inverted microscope (Leica, Mannheim, Germany). Pictures were taken using this equipment, which is attached to a Leica DFC365 FX camera, and the images were visualized and processed using the Leica Application Suite Advanced Fluorescence 3.1.0 software.

## Additional file

Additional file 1: **Alignment.** Nucleotide and amino acid alignment of AF126284 and BR/P05. **Figure S1.** Workflow depicting the protocol used for DENV neutralization and production of AURAV viral stock (BR/P07) from BR/P05. **Figure S2.** DENV detection in BR/P05. **Figure S3.** Result of BR/P07 titration by plaque assay in C6/36 cells. **Figure S4.** Brightfield images of C6/36 cells infected or not (mock) with 0.1 MOI BR/P07 at 3 days post-infection. **Figure S5.** IFA of AP-61 cells infected or not (mock) with 1 MOI BR/P07. **Figure S6.** IFA of Vero and BHK-21 cells infected or not (mock) with 80 MOI BR/P07. **Figure S7.** RT-PCR testing of P01-P04 for **a** AURAV and **b** DENV. **Figure S8.** nsP3 hydrophobicity plot of AF126284 and BR/P05. **Figure S9.** Mechanism of duplication hypothesis. **Table S1.** BLAST analyses of DOP-PCR sequencing results. **Table S2.** Primers used in RT-PCR and sequencing. **Table S3.** Dataset used in phylogenetic analysis. **Supplementary sequence data**. (PDF 2561 kb)

## Abbreviations

AUD: Alphavirus unique domain
AURAV: Aura virus
CHIKV: Chikungunya virus
CPE: Cytopathic effect
DENV: Dengue virus
DOP-PCR: Degenerate-oligonucleotide primed polymerase chain reaction
EEEV: Eastern equine encephalitis virus
EILV: Eilat virus
FBS: Fetal bovine serum
HVD: Hypervariable domain
IFA: Indirect immunofluorescence assay
mAb: Monoclonal antibody
MOI: Multiplicity of infection
PFU: Plaque forming units
RT: Room temperature
RT-PCR: Reverse transcription associated with polymerase chain reaction
SFV: Semliki Forest virus
SINV: Sindbis virus
TEM: Transmission electron microscopy
WEEV: Western equine encephalitis virus
