# Supplementary material for: A new Aura virus isolate in Brazil shows segment duplication in the variable region of the nsP3 gene
Source: Parasit Vectors. 2018 May 29;11:321. doi: 10.1186/s13071-018-2907-4 (PMC5975265; doi:10.1186/s13071-018-2907-4)
Supplement: Supplementary file 1 — Alignment. Nucleotide and amino acid alignment of AF126284 and BR/P05. Figure S1. Workflow depicting the protocol used for DENV neutralization and production of AURAV viral stock (BR/P07) from BR/P05. Figure S2. DENV detection in BR/P05. Figure S3. Result of BR/P07 titration by plaque assay in C6/36 cells. Figure S4. Brightfield images of C6/36 cells infected or not (mock) with 0.1 MOI BR/P07 at 3 days post-infection. Figure S5. IFA of AP-61 cells infected or not (mock) with 1 MOI BR/P07. Figure S6. IFA of Vero and BHK-21 cells infected or not (mock) with 80 MOI BR/P07. Figure S7. RT-PCR testing of P01-P04 for a AURAV and b DENV. Figure S8. nsP3 hydrophobicity plot of AF126284 and BR/P05. Figure S9. Mechanism of duplication hypothesis. Table S1. BLAST analyses of DOP-PCR sequencing results. Table S2. Primers used in RT-PCR and sequencing. Table S3. Dataset used in phylogenetic analysis. Supplementary sequence data. (PDF 2561 kb) [file 13071_2018_2907_MOESM1_ESM.pdf]

```
5'NCR
                                    10                  20                  30                  40
AF126284   A T A G C G G A C G G A C T A G T A C T T G T A C T A C A G A A T T A A C T G C
BR/P05     . . . . T . . . . . . . . . . . . . . . . . . . . . . . . . . . . . . . . . . .

                                    50                  60                  70      nsP1        80
AF126284   C G T G T G C C G C C C G C T A A A C T A G C C C C A A T C A T C G A A A A T G
BR/P05     . . . . . . . . . . . . . . . . . . . . . . . . . . . . . . . . . . . . . . . .

                                    90                 100                 110                 120
AF126284   G A G A A A C C G A C A G T G C A C G T T G A C G T A G A C C C C C A A A G T C
BR/P05     . . . . . . . . . . . . . . . . . . . . . . . . . . . . . . . . . . . . . . . .

                                   130                 140                 150                 160
AF126284   C G T T T G T G C T A C A A C T G C A G A A G A G T T T C C C A C A A T T C G A
BR/P05     . . . . . . . . . . . . . . . . . . . . . . . . . . . . . . . . . . . . . . . .

                                   170                 180                 190                 200
AF126284   G A T T G T G G C T C A G C A G G T C A C T C C G A A T G A C C A T G C T A A T
BR/P05     . . . . . . . . . . . G . . . . . . . . . . . . . . . . . . . . . . . . . . . .

                                   210                 220                 230                 240
AF126284   G C C A G A G C T T T T T C G C A T C T G G C T A G T A A A C T G A T C G A A C
BR/P05     . . . . . . . . . . . . . . . . . . . . . . . . . . . . . . . . . . . . . . . .

                                   250                 260                 270                 280
AF126284   A T G A G A T C C C C A C C T C A G T T A C G A T C T T G G A C A T A G G A A G
BR/P05     . . . . . . . . . . . . . . . . . . . . . . . . . . . . . . . . . . . . . . . .

                                   290                 300                 310                 320
AF126284   C G C A C C A G C T C G T A G A A T G T A T T C C G A G C A T A A G T A T C A C
BR/P05     . . . . . . . . . . . . . . . . . . . . . . . . . . . . . . . . . . . . . . . .

                                   330                 340                 350                 360
AF126284   T G T G T G T G C C C C A T G C G T A G T C C T G A A G A C C C G G A C C G T C
BR/P05     . . . . . . . . . . . . . . . . . . . . . . . . . . . . . . . . . . . . . . . .

                                   370                 380                 390                 400
AF126284   T T A T G A A T T A C G C A T C C C G A C T C G C A G A C A A A G C A G G G G A
BR/P05     . . . . . . . . . . . . . . . . . . . . . . . . . . . . . . . . . . . . . . . .

                                   410                 420                 430                 440
AF126284   A A T T A C C A A C A A G A G G C T G C A T G A T A A A C T T G C A G A C C T C
BR/P05     . . . . . . . . . . . . . . . . . . . . . . . . . . . . . . . . . . . . . . . .

                                   450                 460                 470                 480
AF126284   A A G T C G G T C C T C G A G T C G C C G G A T G C T G A A A C T G G T A C C A
BR/P05     . . . . . . . . . . . . . . . . . . . . . . . . . . . . . . . . . . . . . . . .

                                   490                 500                 510                 520
AF126284   T T T G T T T C C A C A A T G A C G T A A T A T G C C G T A C G A C A G C G G A
BR/P05     . . . . . . . . . . . . . . . . . . . . . C . . . . . . . . . . . . . . . . . .
```

```
                 530       540       550       560
          ....|....|....|....|....|....|....|....|
AF126284  GGTATCAGTTATGCAAAATGTGTATATCAATGCACCTTCG
BR/P05    ................G...........G...........

                 570       580       590       600
          ....|....|....|....|....|....|....|....|
AF126284  ACCATTTACCATCAGGCCCTAAAGGGAGTCAGAAAACTGT
BR/P05    ........................................

                 610       620       630       640
          ....|....|....|....|....|....|....|....|
AF126284  ATTGGATCGGGTTCGATACAACGCAGTTTATGTTCTCCTC
BR/P05    ........................................

                 650       660       670       680
          ....|....|....|....|....|....|....|....|
AF126284  GATGGCAGGGTCGTATCCGTCCTACAATACTAATTGGGCC
BR/P05    ........................................

                 690       700       710       720
          ....|....|....|....|....|....|....|....|
AF126284  GATGAAAGGGTGCTGGAAGCGCGTAATATAGGCCTATGTA
BR/P05    ....................A...................

                 730       740       750       760
          ....|....|....|....|....|....|....|....|
AF126284  GCACGAAGCTGAGAGAGGGTACGATGGGCAAACTGTCTAC
BR/P05    ............C..........................T

                 770       780       790       800
          ....|....|....|....|....|....|....|....|
AF126284  CTTCCGGAAAAAGGCCTTGAAACCTGGAACTAACGTGTAC
BR/P05    .C.............................G........

                 810       820       830       840
          ....|....|....|....|....|....|....|....|
AF126284  TTCTCTGTCGGTTCGACACTCTACCCTGAGAATAGAGCGG
BR/P05    ........................................

                 850       860       870       880
          ....|....|....|....|....|....|....|....|
AF126284  ACCTGCAGAGTTGGCACCTACCATCTGTGTTCCACTTGAA
BR/P05    ........................................

                 890       900       910       920
          ....|....|....|....|....|....|....|....|
AF126284  AGGTAAACAATCCTTTACGTGCCGCTGTGATACGGCGGTT
BR/P05    ........................................

                 930       940       950       960
          ....|....|....|....|....|....|....|....|
AF126284  AACTGCGAAGGATACGTAGTCAAGAAGATCACCATCAGCC
BR/P05    ........................................

                 970       980       990      1000
          ....|....|....|....|....|....|....|....|
AF126284  CCGGGATCACGGGGCGTGTCAATCGGTACACTGTGACTAA
BR/P05    .......................G.C...G..........

                1010      1020      1030      1040
          ....|....|....|....|....|....|....|....|
AF126284  CAACAGCGAGGGATTCTTGCTGTGTAAGATCACAGATACG
BR/P05    ........................................
```

```
                1050      1060      1070      1080
AF126284  GTCAAAGGGGAGCGTGTATCGTTCCCTGTCTGTACGTATA
BR/P05    ........................................

                1090      1100      1110      1120
AF126284  TTCCACCTTCAATCTGTGACCAAATGACAGGTATATTGGC
BR/P05    ........................................

                1130      1140      1150      1160
AF126284  CACTGATATCCAACCCGAAGACGCGCAAAAGTTGCTGGTA
BR/P05    ........................................

                1170      1180      1190      1200
AF126284  GGACTGAACCAACGCATAGTCGTGAACGGAAAAACTAATA
BR/P05    ........................................

                1210      1220      1230      1240
AF126284  GAAACACCAACACGATGCAGAACTATCTCCTGCCCGCGGT
BR/P05    ............................G...........

                1250      1260      1270      1280
AF126284  GGCTACAGGTCTGAGTAAATGGGCCAAAGAAAGAAAGGCA
BR/P05    ........................................

                1290      1300      1310      1320
AF126284  GACTGCAGTGACGAGAAACCATTGAATGTGAGAGAACGCA
BR/P05    ...GC.GA..........................G.....

                1330      1340      1350      1360
AF126284  AACTAGCTTTCGGTTGCCTATGGGCTTTCAAGACCAAGAA
BR/P05    ........................................

                1370      1380      1390      1400
AF126284  GATCCATTCTTTTTACCGCCCGCCAGGCACGCAGACTATA
BR/P05    ........................................

                1410      1420      1430      1440
AF126284  GTAAAAGTCGCAGCGGAATTCAGTGCGTTCCCTATGTCCT
BR/P05    ........................................

                1450      1460      1470      1480
AF126284  CGGTGTGGACTACGTCACTGCCAATGTCACTGAGACAGAA
BR/P05    ........................................

                1490      1500      1510      1520
AF126284  AGTTAAACTGCTTCTTGTAAAGAAAACCAATAAACCGGTA
BR/P05    ........................................

                1530      1540      1550      1560
AF126284  GTCACTATTACTGACACTGCGGTAAAAAACGCACAAGAGG
BR/P05    ...................A....................
```

```
                  1570      1580      1590      1600
AF126284   CATATAACGAAGCCGTCGAGACAGCAGAAGCGGAGGAGAA
BR/P05     ........................................

                  1610      1620      1630      1640
AF126284   AGCGAAGGCCTTACCTCCGCTGAAGCCGACGGCACCCCCT
BR/P05     ........................................

                  1650      1660      1670      1680
AF126284   GTAGCGGAGGACGTCAAATGCGAGGTCACCGACCTGGTAG
BR/P05     ........................................

                  1690 nsP2 →    1700      1710      1720
AF126284   ACGATGCGGGAGCGGCCCTGGTCGAGACGCCCCGGGGAAA
BR/P05     ........................................

                  1730      1740      1750      1760
AF126284   GATAAAAATTATCCCACAGGAAGGGGACGTGCGTATTGGT
BR/P05     ........................................

                  1770      1780      1790      1800
AF126284   TCCTACACAGTCATTTCTCCAGCGGCAGTCCTTAGAAATC
BR/P05     ........................................

                  1810      1820      1830      1840
AF126284   AACAACTGGAGCCAATCCACGAGTTAGCAGAGCAGGTGAA
BR/P05     ........................................

                  1850      1860      1870      1880
AF126284   AATTATCACGCACGGTGGCCGAACAGGCAGGTATTCCGTC
BR/P05     ........................................

                  1890      1900      1910      1920
AF126284   GAACCTTACGATGCTAAGGTTCTCCTGCCAACAGGATGCC
BR/P05     .....................................C.G

                  1930      1940      1950      1960
AF126284   CCATGTCCTGGCAACATTTCGCGGCCTTGAGCGAAAGCGC
BR/P05     ........................................

                  1970      1980      1990      2000
AF126284   TACGTTAGTCTACAATGAGAGAGAGTTCCTGAACCGGAAA
BR/P05     A.....................................G.

                  2010      2020      2030      2040
AF126284   CTCCATCACATCGCTACGAAGGGTGCGGCAAAAAACACTG
BR/P05     ........................................

                  2050      2060      2070      2080
AF126284   AGGAAGAACAATACAAAGTATGCAAAGCTAAAGACACGGA
BR/P05     ........................................
```

```
                              2090      2100      2110      2120
AF126284   TCATGAGTACGTATACGACGTAGATGCCAGAAAATGCGTA
BR/P05     ........................................

                              2130      2140      2150      2160
AF126284   AAAAGAGAGCATGCACAAGGGCTAGTACTAGTTGGGGAAC
BR/P05     ........................................

                              2170      2180      2190      2200
AF126284   TAACTAATCCGCCTTACCACGAGCTGGCATACGAAGGATT
BR/P05     ........................................

                              2210      2220      2230      2240
AF126284   ACGTACACGACCCGCTGCCCCTTACCATATCGAAACACTG
BR/P05     ........................................

                              2250      2260      2270      2280
AF126284   GGGGTCATTGGAACACCGGGGTCAGGTAAGTCGGCCATCA
BR/P05     ........................................

                              2290      2300      2310      2320
AF126284   TAAAATCTACGGTAACACTAAAAGACCTCGTAACTAGCGG
BR/P05     ........................................

                              2330      2340      2350      2360
AF126284   TAAGAAAGAAAATTGCAAAGAAATAGAGAATGACGTCCAG
BR/P05     ............................G...........

                              2370      2380      2390      2400
AF126284   AAAATGCGGGGAATGACTATAGCTACGAGAACGGTAGACT
BR/P05     ........................................

                              2410      2420      2430      2440
AF126284   CGGTACTTCTTAATGGATGGAAGAAAGCAGTAGACGTCCT
BR/P05     ........................................

                              2450      2460      2470      2480
AF126284   ATATGTGGATGAAGCGTTTGCATGTCATGCAGGCACCTTA
BR/P05     ........................................

                              2490      2500      2510      2520
AF126284   ATGGCATTGATTGCCATTGTCAAACCGAGACGTAAAGTAG
BR/P05     ........................................

                              2530      2540      2550      2560
AF126284   TACTGTGCGGCGACCCGAAGCAGTGGCCCTTCTTTAATTT
BR/P05     ..........T..............CGG............

                              2570      2580      2590      2600
AF126284   AATGCAACTGAAGGTAAACTTCAACAACCCCGAGCGAGAC
BR/P05     ........................................
```

```
                2610      2620      2630      2640
          ....|....|....|....|....|....|....|....|
AF126284  CTGTGTACTTCCACCCATTATAAATATATCTCTCGCAGGT
BR/P05    ........................................

                2650      2660      2670      2680
          ....|....|....|....|....|....|....|....|
AF126284  GCACCCAACCTGTTACAGCCATAGTGTCTACATTACACTA
BR/P05    ........................................

                2690      2700      2710      2720
          ....|....|....|....|....|....|....|....|
AF126284  TGACGGAAAGATGAGGACTACGAATCCCTGCAAAAGGGCT
BR/P05    ........................................

                2730      2740      2750      2760
          ....|....|....|....|....|....|....|....|
AF126284  ATCGAAATAGACGTAAACGGATCGACTAAGCCCAAGAAAG
BR/P05    ........................................

                2770      2780      2790      2800
          ....|....|....|....|....|....|....|....|
AF126284  GAGACATAGTGTTGACGTGTTTCCGTGGGTGGGTTAAGCA
BR/P05    ...................C....................

                2810      2820      2830      2840
          ....|....|....|....|....|....|....|....|
AF126284  GGGGCAAATCGATTACCCCGGACCCGGAGGTCATGACCGT
BR/P05    .CTC...................A..AG.TCATGAC.GC.

                2850      2860      2870      2880
          ....|....|....|....|....|....|....|....|
AF126284  GCAGCTTCTCAAGGGCTAACCAGAAGGGGCGTTTATGCGG
BR/P05    .......................G.A..............

                2890      2900      2910      2920
          ....|....|....|....|....|....|....|....|
AF126284  TCAGACAGAAAGTAAATGAAAACCCACTATATGCAGAGAA
BR/P05    ........................................

                2930      2940      2950      2960
          ....|....|....|....|....|....|....|....|
AF126284  GTCAGAACACGTTAACGTGTTACTTACTAGGACGGAAGAT
BR/P05    ........................................

                2970      2980      2990      3000
          ....|....|....|....|....|....|....|....|
AF126284  CGCATAGTGTGGAAGACACTGCAAGGGGATCCTTGGATTA
BR/P05    ................................C.......

                3010      3020      3030      3040
          ....|....|....|....|....|....|....|....|
AF126284  AGTACCTCACTAACGTTCCAAAAGGGAACTTTACAGCCAC
BR/P05    ........................................

                3050      3060      3070      3080
          ....|....|....|....|....|....|....|....|
AF126284  TTTAGAAGAATGGCAGGCGGAACACGAGGACATTATGAAG
BR/P05    ........................................

                3090      3100      3110      3120
          ....|....|....|....|....|....|....|....|
AF126284  GCCATTAATTCTACATCCACAGTATCTGACCCTTTCGCCA
BR/P05    .....C..................................
```

```
              3130      3140      3150      3160
AF126284  GCAAAGTGAATACATGCTGGGCTAAAGCTATTATACCCAT
BR/P05    ........................................

              3170      3180      3190      3200
AF126284  CCTAAGAACGGCAGGGATAGAACTTACATTCGAGCAGTGG
BR/P05    ........................................

              3210      3220      3230      3240
AF126284  GAAGATCTATTCCCGCAATTTCGTAATGACCAACCTTACT
BR/P05    ........................................

              3250      3260      3270      3280
AF126284  CCGTGATGTATGCCCTAGATGTGATATGTACCAAGATGTT
BR/P05    ...C...T......T........G................

              3290      3300      3310      3320
AF126284  CGGCATGGATCTGAGCAGTGGGATCTTCTCTCGTCCTGAG
BR/P05    ........................................

              3330      3340      3350      3360
AF126284  ATACCTCTAACGTTCCATCCCGCGGACGTCGGCCGAGTGA
BR/P05    ...........................A............

              3370      3380      3390      3400
AF126284  GAGCTCACTGGGATAACTCCCCAGGAGGGCAGAAGTTTGG
BR/P05    ........................................

              3410      3420      3430      3440
AF126284  GTATAACAAGGCGGTAATCCCAACT---TGCAAGAAATAC
BR/P05    ...................G.C.ACTTGGCG.........

              3450      3460      3470      3480
AF126284  CCAGTGTACTTAAGAGCAGGAAAAGGGGACCAAATACTCC
BR/P05    ........................................

              3490      3500      3510      3520
AF126284  CCATATATGGCAGAGTTTCAGTCCCATCGGCACGGAACAA
BR/P05    ..G.....................................

              3530      3540      3550      3560
AF126284  TTTAGTTCCCTTAAACAGAAATCTACCACACTCGCTAACT
BR/P05    ........................................

              3570      3580      3590      3600
AF126284  GCAAGCCTGCAGAAAAAAGAAGCAGCTCCCTTGCACAAGT
BR/P05    ........................................

              3610      3620      3630      3640
AF126284  TCCTTAACCAACTACCAGGACACAGTATGCTGCTGGTCTC
BR/P05    ........................................
```

```
                 3650      3660      3670      3680
AF126284  TAAGGAAACATGCTATTGCGTGTCCAAGCGAATCACATGG
BR/P05    ........................................

                 3690      3700      3710      3720
AF126284  GTCGCTCCGCTGGGAGTCAGAGGAGCTGACCACAACCATG
BR/P05    ........................................

                 3730      3740      3750      3760
AF126284  ACCTGCATTTCGGGTTCCCACCACTGTCCAGATACGACCT
BR/P05    ........................................

                 3770      3780      3790      3800
AF126284  TGTGGTGGTTAATATGGGACAACCGTACAGGTTCCATCAC
BR/P05    .................ACAC...................

                 3810      3820      3830      3840
AF126284  TACCAGCAGTGCGAGGAGCATGCCGGCCTCATGAGGACGT
BR/P05    ........................................

                 3850      3860      3870      3880
AF126284  TGGCCCGGTCAGCACTCAACTGCCTAAAACCAGGAGGAAC
BR/P05    ...............................T..T.....

                 3890      3900      3910      3920
AF126284  ATTAGCCCTGAAAGCATATGGTTTCGCCGACTCCAATAGT
BR/P05    ........................................

                 3930      3940      3950      3960
AF126284  GAGGACGTTGTTCTGTCTTTAGCGAGGAAATTCGTGCGGG
BR/P05    .................C......................

                 3970      3980      3990      4000
AF126284  CATCCGCAGTGAGACCATCGTGTACACAGTTTAACACAGA
BR/P05    T.......................................

                 4010      4020      4030      4040
AF126284  GATGTTCTTTGTATTTAGGCAGCTGGACAACGATCGTGAG
BR/P05    ........................................

                 4050      4060      4070      4080
AF126284  CGCCAATTCACTCAGCATCACTTGAATTTAGCAGTATCCA
BR/P05    ........................................

                                       nsP3 →
                 4090      4100      4110      4120
AF126284  ATATATTCGACAATTATAAAGACGGATCCGGAGCAGCTCC
BR/P05    ........................................

                 4130      4140      4150      4160
AF126284  TTCTTATCGCGTTAAGAGAATGAATATCGCAGACTGCACA
BR/P05    ........................................
```

```
                 4170      4180      4190      4200
AF126284  G A A G A A G C A G T G G T G A A C G C A G C T A A C G C G C G G G G A A A A C
BR/P05    . . . . . . . . . . . . . . . . . . . . . . . . . . . . . . G C . . . . . . . .

                 4210      4220      4230      4240
AF126284  C T G G G G A C G G A G T A T G C A G A G C T A T C T T C A A A A A G T G G C C
BR/P05    . . . . . . . . . . . . . . . . . . . . . . . . . . . . . . . . . . . . . . . .

                 4250      4260      4270      4280
AF126284  G A A G T C A T T T G A G A A C G C T A C C A C T G A A G T G G A A A C C G C G
BR/P05    . . . . . . . . . . . . . . . . . . . . . . . . . . . . . . . . G . . . . . . .

                 4290      4300      4310      4320
AF126284  G T C A T G A A A C C A T G C C A C A A C A A G G T T G T T A T A C A T G C A G
BR/P05    . . . . . . . . . . . . . . . . . . . . . . . . . . . . . . . . . . . . . . . .

                 4330      4340      4350      4360
AF126284  T G G G T C C T G A T T T T A G A A A G T A C A C G T T G G A G G A A G C G A C
BR/P05    . . . . . . . . A . . . . . . . . . . . . . . . . . . . . . . . . . . . . . . .

                 4370      4380      4390      4400
AF126284  G A A G C T A C T G C A G A A C G C A T A C C A T G A T G T G G C A A A G A T A
BR/P05    . . . . . . . . . . . . . . . . . . . . . . . . . . . . . . . . . . . . . . . .

                 4410      4420      4430      4440
AF126284  G T G A A C G A G A A A G G C A T C T C C T C G G T A G C T A T A C C G C T G C
BR/P05    . . . . . . . . . . . . . . . . . . . . . . . . . . . . . . . . . . . . . . . .

                 4450      4460      4470      4480
AF126284  T C T C A A C A G G T A T C T A T G C T G C C G G A G C T G A T C G C C T G G A
BR/P05    . . . . . . . . A . . . . . . . . . . . . . . . . . . . . . . . . . . . . . . .

                 4490      4500      4510      4520
AF126284  T C T C T C G C T G A G A T G T C T T T T C A C C G C G C T G G A T C G T A C G
BR/P05    . . . . . . . . . . . . . . . . . . . . . T . . . . . . . . . . . . . . . . . .

                 4530      4540      4550      4560
AF126284  G A T G C G G A T G T C A C A A T A T A T T G C C T A G A T A A G A A G T G G G
BR/P05    . . . . . . . . . . . . . . . . . . . . . . . . . . . . . . . . . . . . . . . .

                 4570      4580      4590      4600
AF126284  A G C A A C G C A T A G C A G A T G C T A T T A G G A T G C G A G A A C A A G T
BR/P05    . . . . . . . . . . . . . . . . . . . . . . . . . . . . . . . . . . . . . C . .

                 4610      4620      4630      4640
AF126284  A A C T G A A T T A A A A G A T C C G G A C A T A G A G A T A G A T G A A G G A
BR/P05    . . . . . . . . . . . . . . . . . . . . . . . . . . . . . . . . . . . . . . . .

                 4650      4660      4670      4680
AF126284  T T A A C C C G G G T A C A C C C A G A T A G C T G C C T C A A G G A T C A C A
BR/P05    . . . . . . . . . . . . . . . . . . . . . . . . . . . . . . . . . . . . . . . .
```

```
                                    4690      4700      4710      4720
AF126284   TAGGCTACAGTACCCAGTATGGGAAATTGTACTCATACTT
BR/P05     ..............AGTATG....................

                                    4730      4740      4750      4760
AF126284   TGAAGGTACTAAATTCCACCAAACCGCAAAAGACATAGCC
BR/P05     ....................G...................

                                    4770      4780      4790      4800
AF126284   GAGATTCGTGCGCTGTTTCCTGATGTACAAGCCGCTAACG
BR/P05     ........................................

                                    4810      4820      4830      4840
AF126284   AACAAATCTGCCTGTACACTTTAGGCGAACCGATGGAGTC
BR/P05     ........................................

                                    4850      4860      4870      4880
AF126284   CATACGCGAAAAGTGCCCAGTCGAAGACTCCCCGGCATCA
BR/P05     ........................................

                                    4890      4900      4910      4920
AF126284   GCACCTCCTAAGACAATACCTTGCCTATGTATGTATGCTA
BR/P05     ........................................

                                    4930      4940      4950      4960
AF126284   TGACAGCCGAACGTATTTGCCGCGTACGCAGTAACTCCGT
BR/P05     ......................GC................

                                    4970      4980      4990      5000
AF126284   AACGAACATAACGGTGTGCTCATCCTTTCCGTTACCCAAG
BR/P05     ........................................

                                    5010      5020      5030      5040
AF126284   TACCGAATAAAGAACGTACAAAAGATACAGTGCACGAAAG
BR/P05     ........................G...............

                                    5050      5060      5070      5080
AF126284   TCGTGCTATTTAACCCGGATGTACCGCCCTACATTCCCGC
BR/P05     .T......................................

                                    5090      5100      5110      5120
AF126284   GAGAGTATACATTAACAAGGACGAGCCTCCTGTTACTCCC
BR/P05     ........................................

                                    5130      5140      5150      5160
AF126284   CATACCGACAGCCCGCCGGACACTTGCTCTTCGAGGCTAT
BR/P05     ........................................

                                    5170      5180      5190      5200
AF126284   CGCTGACGCCCACGCTCTCTAACGCAGAATCGGATATCGT
BR/P05     ........................................
```

```
                                  5210                5220                5230                5240
AF126284   ATCTCTAACGTTTTCGGAAATCGATAGCGAGCTGTCGTCA
BR/P05     ...............A........................

                                  5250                5260                5270                5280
AF126284   CTAAACGAGCCTGCGAGGCACGTAATGATATCCTCGTTCA
BR/P05     ...............GA.GCACGT.ATGATAT.CTCG.TC

                                  5290                5300                5310                5320
AF126284   AGCTGAGGTACACTGCGATCCAGGCTTTACCTCAGAAGCT
BR/P05     .AGCTGA.GTACACTGCGAT.CA.GC..TA.CTCAG.AGC

                                  5330                5340                5350                5360
AF126284   GAGTTGGATGAGAGAGGATCGCACACCGAGACAGCCGCCT
BR/P05     TGAG.T.GATGAGAGA.GATCG..................

                                  5370                5380                5390                5400
AF126284   CCTGTACCGCCACCACGACCAAAACGAGCCGCGAAATTAT
BR/P05     ........................................

                                  5410                5420                5430                5440
AF126284   CCCGACTAGCAAACCAG-----------------------
BR/P05     ................CGCAGAATCGGATATCGTATCTCT

                                  5450                5460                5470                5480
AF126284   ----------------------------------------
BR/P05     AACGTTTTCAGAAATCGATAGCGAGCTGTCGTCACTAAAC

                                  5490                5500                5510                5520
AF126284   ----------------------------------------
BR/P05     GAGCCTGCGGAGGCACGTAATGATATCCTCGTTCAAGCTG

                                  5530                5540                5550                5560
AF126284   ----------------------------------------
BR/P05     AGGTACACTGCGATCCAGGCTTTACCTCAGAAGCTGAGTT

                                  5570                5580                5590                5600
AF126284   ----------------------------------------
BR/P05     GGATGAGAGAGGATCGACACCGAGACAGCCGCCTCCTGTA

                                  5610                5620                5630                5640
AF126284   ----------------------------------------
BR/P05     CCGCCACCACGACCAAAACGAGCCGCGAAATTATCCCGAC

                                  5650                5660                5670                5680
AF126284   -----------CTAAACGAGCTACGGAGGCATGCGACGAT
BR/P05     TAGCAAACCAG..........CTAC.GA.GCATGCGACGA

                                  5690                5700                5710                5720
AF126284   ATCCTCCGTTCAAGCTGAGGTACACTACAATTCAGGTTTT
BR/P05     TAT.CT..................................
```

```
                   5730      5740      5750      5760
AF126284  ACCCCAGAAGCCGAATTGAATGAGAGGATCGATACTGA
BR/P05    ........................G...............

                   5770      5780      5790      5800
AF126284  GGAAGCCGCCTCCCGTACCGCCACTACGACCAAAACAAAC
BR/P05    ........................................

                   5810      5820      5830      5840
AF126284  TACGAACTTATCTCGACTCGCAAACCAACTATCTATGCCG
BR/P05    ........................................

                   5850      5860      5870      5880
AF126284  ATAACATTCGGAGATTTTGCCGAAGGAGAGCTAGACAGGC
BR/P05    ........................................

                   5890      5900      5910      5920
AF126284  TGCTAACGCCATCACCGACCCCTACGTTTGGGGACTTCTC
BR/P05    ........................................

                   5930      5940      5950      5960
AF126284  TCAGGAAGAAATGGATAGATTCTTCGGAAACAGACAATAT
BR/P05    ........................................

                                 nsP4 →
                   5970                5990      6000
AF126284  TGACTAACCGGGGTAGGTGGGTACATATTCTCTTCTGATA
BR/P05    C.....................T.................

                   6010      6020      6030      6040
AF126284  CAGGCCCGGGGCATCTGCAACAAAAATCAGTTATTCAAAA
BR/P05    ..................................G.....

                   6050      6060      6070      6080
AF126284  CAGCACCACCGAGATACTAATAGAACGAAGTAGACTAGAA
BR/P05    ........................................

                   6090      6100      6110      6120
AF126284  AAAATTCATGCCCCTGTACTGGACCTACAGAAAGAAGAGA
BR/P05    ........................................

                   6130      6140      6150      6160
AF126284  TGCTGAAGTGTAGGTACCAGATGTCACCCACCGTAGCCAA
BR/P05    ........................................

                   6170      6180      6190      6200
AF126284  CAAGAGCAGGTACCAGTCACGGAAAGTAGAGAACATGAAA
BR/P05    ........................................

                   6210      6220      6230      6240
AF126284  GCAGTGACGACAGGGAGGTTGTTAGATGGTCTGAAAATGT
BR/P05    .............T..................A.......
```

```
                   6250      6260      6270      6280
AF126284  ACGTCACCCCGGACGTTGAAGCAGAATGCTACAAGTACAC
BR/P05    ........................................

                   6290      6300      6310      6320
AF126284  GTATCCAAAACCGATGTATTCTGCCAGCGTGCCTGACCGC
BR/P05    ........................................

                   6330      6340      6350      6360
AF126284  TTCGTATCACCTGAAGTGGCAGTAGCGGTCTGCAACAACT
BR/P05    ........................................

                   6370      6380      6390      6400
AF126284  TCTTCCATGAGAATTATCCTACGGTAGCCTCTTACCAGAT
BR/P05    ....T...........C.......................

                   6410      6420      6430      6440
AF126284  AACTGACGAGTACGATGCCTACTTAGACATGGTCGAGGGG
BR/P05    ...............................C....C...

                   6450      6460      6470      6480
AF126284  TCTGTATCGTGTTTAGATACTGCTACATTCTGCCCAGCGA
BR/P05    ........................................

                   6490      6500      6510      6520
AF126284  AACTGAGGAGCTTCCCAAAGACACACTCTTACCTGGAACC
BR/P05    .....---................................

                   6530      6540      6550      6560
AF126284  TACGTTGCGGAGTGCGGTTCCATCGGCGTTTCAGAACACC
BR/P05    ..............................C.........

                   6570      6580      6590      6600
AF126284  CTGCAAAACGTATTATCAGCAGCTACTAAAAGGAACTGCA
BR/P05    ........................................

                   6610      6620      6630      6640
AF126284  ATGTAACACAAATGAGAGAACTGCCTGTGCTAGATTCCGC
BR/P05    ........................................

                   6650      6660      6670      6680
AF126284  CGTGTTCAATGTGGAGTGCTTTAAAAAGTATGCATGTAAT
BR/P05    ....C...................................

                   6690      6700      6710      6720
AF126284  ACAGACTACTGGGAAGAATTTAAAGAGAAACCAATAAGAA
BR/P05    ........................................

                   6730      6740      6750      6760
AF126284  TAACAACAGAGTGTGTCACCTCATACGTGGCCCGATTAAA
BR/P05    ........................................
```

```
                  6770      6780      6790      6800
AF126284  AGGACCGGAAGCTGCAGCACTATTCGCAAAGACACATCAA
BR/P05    .......A.G............................G.

                  6810      6820      6830      6840
AF126284  CTGGTCCCGCTACAGGAGGTCCCTATGGATAGGTTTGTAA
BR/P05    ........................................

                  6850      6860      6870      6880
AF126284  TGGACATGAAAAGAGATGTAAAGGTGACACCCGGCACTAA
BR/P05    ........................................

                  6890      6900      6910      6920
AF126284  ACATACCGAAGAGCGTCCCAAGGTGCAAGTTATTCAAGCA
BR/P05    ........................A...............

                  6930      6940      6950      6960
AF126284  GCAGAACCACTGGCCACGGCTTACTTGTGCGGTATACACC
BR/P05    ........................................

                  6970      6980      6990      7000
AF126284  GGGAGCTAGTACGGAGGCTTACAGCAGTACTGTTACCTAA
BR/P05    ........................................

                  7010      7020      7030      7040
AF126284  CATCCATACACTATTCGACATGTCTGCCGAGGATTTCGAC
BR/P05    ........................................

                  7050      7060      7070      7080
AF126284  GCGATCATAGCAGCAAATTTTTCTTACGTGCACCCGGTGC
BR/P05    ............................GTG.........

                  7090      7100      7110      7120
AF126284  TAGAAACAGATATAGGCTCTTTCGACAAGAGCCAGGATGA
BR/P05    ...............CG.......................

                  7130      7140      7150      7160
AF126284  TTCATTAGCCCTGACTGCACTGATGATTCTGGAAGACTTA
BR/P05    ........................................

                  7170      7180      7190      7200
AF126284  GGAGTGGATGACCGCCTGATGGACCTCATTGAATGTGCCT
BR/P05    ........................................

                  7210      7220      7230      7240
AF126284  TCGGGGAGATCACAAGCGTCCACCTACCGACAGCAACCAC
BR/P05    .......................................G

                  7250      7260      7270      7280
AF126284  GTTTAAATTTGGTGCTATGATGAAGTCCGGAATGTTTTTA
BR/P05    ........................................
```

```
                            7290      7300      7310      7320
                   ....|....|....|....|....|....|....|....|
AF126284           ACACTGTTTGTTAACACTGTGTTGAATGTAGTTATTGCTA
BR/P05             ........................................

                            7330      7340      7350      7360
                   ....|....|....|....|....|....|....|....|
AF126284           GCCGAGTTCTAGAACAACGGTTGAGGGACTCCAAATGCGC
BR/P05             ........................................

                            7370      7380      7390      7400
                   ....|....|....|....|....|....|....|....|
AF126284           AGCTTTCATAGGGGACGACAACATTATACATGGTGTCGTT
BR/P05             ........................................

                            7410      7420      7430      7440
                   ....|....|....|....|....|....|....|....|
AF126284           TCGGACAAGATAATGGCCGACAGATGCGCCACTTGGATGA
BR/P05             ........................................

                            7450      7460      7470      7480
                   ....|....|....|....|....|....|....|....|
AF126284           ATATGGAAGTGAAAATCATAGACGCGGTCATTGGTATCAA
BR/P05             .......................A................

                            7490      7500      7510      7520
                   ....|....|....|....|....|....|....|....|
AF126284           GGCCCCTTACTTTTGCGGCGGATTTATTCTAGAAGACCAA
BR/P05             ........................................

                            7530      7540      7550      7560
                   ....|....|....|....|....|....|....|....|
AF126284           GTGACGCACACAGCGTGCCGGGTCTCTGATCCGCTTAAGA
BR/P05             ........................................

                            7570      7580      7590      7600
                   ....|....|....|....|....|....|....|....|
AF126284           GACTGTTCAAACTAGGCAAGCCTCTACCAGTAGATGATGA
BR/P05             ........................................

                            7610      7620      7630      7640
                   ....|....|....|....|....|....|....|....|
AF126284           GCAGGATCACGACAGGCGTAGAGCGCTGGAAGATGAGACA
BR/P05             ........................................

                            7650      7660      7670      7680
                   ....|....|....|....|....|....|....|....|
AF126284           AGGGCGTGGTTCAGAGTGGGGATCCAAGGAGAACTTTTGA
BR/P05             ........................................

                            7690      7700      7710      7720
                   ....|....|....|....|....|....|....|....|
AF126284           AGGCCGTCGAGTCACGCTATGAGGTACAAGAGGTTCAGCC
BR/P05             ........................................

                            7730      7740      7750      7760
                   ....|....|....|....|....|....|....|....|
AF126284           AGTGCTGTTAGCCCTCGCTACGTTCTCGCGTAGCGATAAA
BR/P05             ........................................

                            7770      7780      7790      7800
                   ....|....|....|....|....|....|....|....|
AF126284           GCATTTAAAGCACTACGCGGGAGCCCAAGACACCTCTACG
BR/P05             ........................................
```

```
                                   NCR ->
                              7810                7820                7830                7840
AF126284   G T G G T C C T A A A T A G A C G G T G T A G C A C A G T A G A T C G T C T A A
BR/P05     . . . . . . . . . . . . . . . . . . . . . . . . . . . . . . . . . . . . . . . .

                                                           C ->
                              7850                7860                7870                7880
AF126284   A T T A T T T C A C A T C T T T A C G T T A C T A T G A A C T C T G T C T T T T
BR/P05     . . . . . . . . . . . . . . . . . . . . . . . . . . . . . . . . . . . . . . . .

                              7890                7900                7910                7920
AF126284   A C A A T C C G T T T G G C C G A G G T G C C T A C G C T C A A C C T C C A A T
BR/P05     . . . . . . . . . . . . . T . . . . . . . . . . . . . . . . . . . . . . . . . .

                              7930                7940                7950                7960
AF126284   A G C A T G G A G G C C A A G A C G T A G G G C T G C A C C T G C G C C T C G A
BR/P05     . . . . . . . . . . . . . . . . . . . . . . C G . . . . . . . . . . . . . . . .

                              7970                7980                7990                8000
AF126284   C C A T C C G G G T T G A C T A C C C A G A T C C A A C A G C T C A C T A G G G
BR/P05     . . . . . . . . . . . . . . . . . . . . . . . . . . . . . . . . . . . . . . . .

                              8010                8020                8030                8040
AF126284   C T G T T A G A G C T T T G G T G C T G G A C A A T G C T A C A C G T C G C C A
BR/P05     . . . . . G . . . . . . . . . . . . . . . . . . . . . . . . . . . . . . . . . .

                              8050                8060                8070                8080
AF126284   G C G C C C G G C T C C T C G C A C G C G C C C G A G G A A G C C G A A G A C T
BR/P05     . . . . . . . . . . . . . . . . . . . G C G . . . . . . . . . . . . . . . . . .

                              8090                8100                8110                8120
AF126284   C A A A A A C C T A A G C C G A A G A A G C A A A A C C A G A A A C C A C C A C
BR/P05     . . . . . . . . . . . . . . . . . . . . . . . . . . . . . . . . . . . . . . . .

                              8130                8140                8150                8160
AF126284   A A C A G C A G A A G A A A G G G A A A A A T C A G C C C C A A C A A C C G A A
BR/P05     . . . . . . . . . . . . . . . . . . . . . . . . . . . . . . . . . . . . . . . .

                              8170                8180                8190                8200
AF126284   G A A A C C G A A G C C C G G T A A A C G A C A G C G T A C C G C C C T G A A A
BR/P05     . . . . . . . . . . . . . . . C . . . . . . . . . . . . . . . . . . . . . . . .

                              8210                8220                8230                8240
AF126284   T T T G A A G C C G A C C G C A C A T T T G T C G G G A A G A A T G A A G A C G
BR/P05     . . . . . . . . . . . . . . . . . . . . . . C G . T . . . . . . . . . . . . . .

                              8250                8260                8270                8280
AF126284   G C A A G A T T A T G G G A T A C G C C G T T G C C A T G G A A G G G A A A G T
BR/P05     . . . . . . . . . . . . . . . . . . . . . . . . . . . . . . . . . . . . . . . .

                              8290                8300                8310                8320
AF126284   G A T A A A A C C A C T A C A T G T A A A A G G A A C C A T T G A C C A C C C G
BR/P05     . . . . . . . . . . . . . . . . . . . . . . . . . . . . . . . . . . . . . . . .
```

```
                                   8330                8340                8350                8360
AF126284   GCCCTAGCGAAACTTAAATTCACTAAATCTTCTTCTTACG
BR/P05     .................C......................

                                   8370                8380                8390                8400
AF126284   ACATGGAGTTTGCTAAACTACCGACCGAAATGAAAAGCGA
BR/P05     ...C....................................

                                   8410                8420                8430                8440
AF126284   CGCATTCGGGTATACAACGGAACACCCCGAAGTATTTTAC
BR/P05     .......A........................G.......

                                   8450                8460                8470                8480
AF126284   AACTGGCATCACGGAGCTGTCCAATTTTCCGGCGGAAGGT
BR/P05     ........................................

                                   8490                8500                8510                8520
AF126284   TCACCATCCCTACAGGAGTCGGAGGCCCCGGAGATAGCGG
BR/P05     ..................C......G..............

                                   8530                8540                8550                8560
AF126284   AAGGCCTATACTGGATAACTCCGGAAAAGTGGTAGCCATA
BR/P05     ........................................

                                   8570                8580                8590                8600
AF126284   GTCCTAGGAGGAGCTAATGAAGTGCCAGGAACGGCACTTT
BR/P05     ......................GTG.CA............

                                                        E3
                                   8650                8660                8670                8680
AF126284   CACCCACGAAGATACTGTAGAGTGGTCGCGGGCTATTACC
BR/P05     ...........................CGC..........

                                   8690                8700                8710                8720
AF126284   GCTATGTGCATCCTGCAGAACGTCACATTCCCATGTGACC
BR/P05     ........................................

                                   8730                8740                8750                8760
AF126284   GACCGCCAACTTGCTATAATCGTAATCCTGACTTGACCCT
BR/P05     ........................................

                                   8770                8780                8790                8800
AF126284   AACCATGTTGGAAACAAATGTCAATCACCCTTCGTACGAC
BR/P05     ........................................

                                   8810                8820                8830                8840
AF126284   GTTCTGCTGGACGCTGCTCTGAGGTGCCCCACGAGACGGC
BR/P05     ................................C.......
```

E2

```
                                     8850      8860      8870      8880
AF126284    ACGTCAGATCAACGCCCACCGATGACTTCACTCTCACAGC
BR/P05      ..........................C.............

                                     8890      8900      8910      8920
AF126284    ACCGTACCTCGGCTTGTGTCACAGATGTAAGACGATGGAA
BR/P05      .......................C................

                                     8930      8940      8950      8960
AF126284    CCATGCTACAGCCCTATAAAAATCGAAAAAGTGTGGGATG
BR/P05      ........................................

                                     8970      8980      8990      9000
AF126284    ATGCCGATGACGGAGTTCTCCGTATACAAGTAAGTGCCCA
BR/P05      ........................................

                                     9010      9020      9030      9040
AF126284    GTTAGGGTACAACAGGGCGGGCACTGCAGCTAGCGCCCGA
BR/P05      ........................................

                                     9050      9060      9070      9080
AF126284    CTCCGGTTCATGGGCGGAGGAGTGCCTCCGGAAATCCAGG
BR/P05      ........................................

                                     9090      9100      9110      9120
AF126284    AGGGAGCAATTGCAGATTTTAAGGTCTTCACGTCCAAACC
BR/P05      ..................................T.....

                                     9130      9140      9150      9160
AF126284    ATGTTTACACCTATCACATAAAGGATACTTTGTCATTGTC
BR/P05      ........................................

                                     9170      9180      9190      9200
AF126284    AAGTGCCCTCCTGGTGATAGTATTACAACATCATTGAAAG
BR/P05      ........................................

                                     9210      9220      9230      9240
AF126284    TGCATGGCTCGGATCAAACCTGCACAATTCCAATGCGAGT
BR/P05      .A......................................

                                     9250      9260      9270      9280
AF126284    AGGTTACAAGTTCGTAGGCAGGGAAAAATATACTCTGCCA
BR/P05      ....................A...................

                                     9290      9300      9310      9320
AF126284    CCAATGCATGGGACACAAATACCTTGCCTTACCTACGAAA
BR/P05      ........................................

                                     9330      9340      9350      9360
AF126284    GGACACGAGAGAAAAGTGCAGGATACGTGACCATGCATCG
BR/P05      ......C.................................
```

```
                           9370                9380                9390                9400
          . . . . | . . . . | . . . . | . . . . | . . . . | . . . . | . . . . | . . . . |
AF126284  T C C C G G A C A A C A A T C C A T A A C C A T G C T G A T G G A A G A G A G C
BR/P05    . . . . . . . . . . . . . . . . . . . . . . . . . . . . . . . . . . . . . . . .

                           9410                9420                9430                9440
          . . . . | . . . . | . . . . | . . . . | . . . . | . . . . | . . . . | . . . . |
AF126284  G G A G G G G A G G T G T A C G T A C A A C C G A C C A G T G G G C G A A A C G
BR/P05    . . . . . . . . . . . . . . . . . . . . . . . . . . . . . . . . . . . . . . . .

                           9450                9460                9470                9480
          . . . . | . . . . | . . . . | . . . . | . . . . | . . . . | . . . . | . . . . |
AF126284  T C A C C T A C G A G T G T A A A T G C G G A G A C T T T A A A A C T G G G A C
BR/P05    . . . . . . . . . . . . . . . . . . . . . . . . . . . . . . . . . . . . . . . .

                           9490                9500                9510                9520
          . . . . | . . . . | . . . . | . . . . | . . . . | . . . . | . . . . | . . . . |
AF126284  T G T C A C T G C G C G C A C T A A A A T A G A C G G C T G T A C A G A A A G G
BR/P05    . . . . . . . . . . . . . . . . . . G . . . . . . . . . . . . . . . . . . . . .

                           9530                9540                9550                9560
          . . . . | . . . . | . . . . | . . . . | . . . . | . . . . | . . . . | . . . . |
AF126284  A A A C A A T G C A T T G C G A T T T C T G C C G A C C A C G T C A A A T G G G
BR/P05    . . . . . . . . . . . . . . . T A . . T C . . . . . . . . . . . . . . . . . . .

                           9570                9580                9590                9600
          . . . . | . . . . | . . . . | . . . . | . . . . | . . . . | . . . . | . . . . |
AF126284  T G T T T A A C T C C C C T G A C T T G A T C A G G C A T A C C G A C C A C A C
BR/P05    . . . . . . . . . . . . . . . . . . . . . . . . . . . . . . . . . . . . . . . .

                           9610                9620                9630                9640
          . . . . | . . . . | . . . . | . . . . | . . . . | . . . . | . . . . | . . . . |
AF126284  A G C C C A A G G G A A G T T G C A T A T A C C A T T C C C G C T A C A G C A G
BR/P05    . . . . . . . . . . . . . . . . . . . . . . . . . . . . . . . . . . . . . . . .

                           9650                9660                9670                9680
          . . . . | . . . . | . . . . | . . . . | . . . . | . . . . | . . . . | . . . . |
AF126284  G C T C A A T G T A C A G T A C C A C T G G C G C A C C T T C C A G G C G T T A
BR/P05    . . . . . . . . . . . . . . . . . . . . . . . . . . . . . . . . . C . . . . . .

                           9690                9700                9710                9720
          . . . . | . . . . | . . . . | . . . . | . . . . | . . . . | . . . . | . . . . |
AF126284  A G C A T G C T T A T C G C A G T A T G T C T C T G A C A C T G C A C G C T G A
BR/P05    . . T . . . . . . . . . . . . . . . . . . . . . . . . . . . . . . . . . . . . .

                           9730                9740                9750                9760
          . . . . | . . . . | . . . . | . . . . | . . . . | . . . . | . . . . | . . . . |
AF126284  G C A T C C T A C A T T G C T T A C T A C C C G C C A T C T T G G A G A A A A T
BR/P05    . . . . . . . . . . . . . . . . . . . . . . . . . . . . . . . . . . . . . . . .

                           9770                9780                9790                9800
          . . . . | . . . . | . . . . | . . . . | . . . . | . . . . | . . . . | . . . . |
AF126284  C C T C A G C C C A C T G C A G A A T G G A T T G T C G G G A G T G T A A C T C
BR/P05    . . . . . . . . . . . . . . . . . . . . . . . . . . . . . . . . . . . . . . . .

                           9810                9820                9830                9840
          . . . . | . . . . | . . . . | . . . . | . . . . | . . . . | . . . . | . . . . |
AF126284  G A A A C T T C T C C A T A A C C A T A C A A G G G T T C G A G T A T A C T T G
BR/P05    . . . . . . . . . . . . . . . . . . . . . . . . . . . . . . . . . . . . . . . .

                           9850                9860                9870                9880
          . . . . | . . . . | . . . . | . . . . | . . . . | . . . . | . . . . | . . . . |
AF126284  G G G A A A T C A G A A A C C G G T C C G A G T G T A C G C G C A G G A A T C G
BR/P05    . . . . . . . . G . . G . . . . . . . . . . . . . . . . . . . . . . . . . . . .
```

```
                  9890      9900      9910      9920
         ....|....|....|....|....|....|....|....|
AF126284 GCACCTGGCAATCCTCATGGCTGGCCACATGAAATCGTAC
BR/P05   ...................................T....

                  9930      9940      9950      9960
         ....|....|....|....|....|....|....|....|
AF126284 GCCATTACTACCACCTCTATCCCTTCTACACCGTTACAGT
BR/P05   ........................................

                  9970      9980      9990     10000
         ....|....|....|....|....|....|....|....|
AF126284 GCTGAGCGGCATGGGACTGGCCATATGCGCTGGCTTAGTG
BR/P05   ........................................

                 10010     10020     10030     10040
         ....|....|....|....|....|....|....|....|
AF126284 ATCAGTATTTTATGCTGCTGCAAAGCAAGAAGGGATTGCC
BR/P05   ........................................

                 10050     10060     10070     10080
         ....|....|....|....|....|....|....|....|
AF126284 TAACACCTTACCAACTGGCCCCGAACGCTACCGTACCATT
BR/P05   ........................................

                 10090     10100     10110     10120
         ....|....|....|....|....|....|....|....|
AF126284 TCTGGTAACATTGTGTTGCTGTTTCCAACGGACTTCAGCG
BR/P05   ..............................AG........

      6K →
                 10130     10140     10150     10160
         ....|....|....|....|....|....|....|....|
AF126284 GATGAATTTACCGATACCATGGGGTACCTATGGCAACACA
BR/P05   ........................................

                 10170     10180     10190     10200
         ....|....|....|....|....|....|....|....|
AF126284 GTCAAACAATGTTCTGGATACAATTGGTCATACCTTTAGC
BR/P05   ........................................

                 10210     10220     10230     10240
         ....|....|....|....|....|....|....|....|
AF126284 AGCAGTGATAACTTTGGTTAGATGTTGCTCCTGCTGTCTA
BR/P05   ........................................

                 10250     10260     10270     10280
         ....|....|....|....|....|....|....|....|
AF126284 CCTTTTTTATTGGTTGCCAGTCCT---CCTAACAAAGCGG
BR/P05   .................AG.CGTCCTC.TA.CA..G....

           E1 →
                 10290     10300     10310     10320
         ....|....|....|....|....|....|....|....|
AF126284 ACGCCTACGAACATACGATCACTGTCCCAAATGCGCCGTT
BR/P05   ........................................

                 10330     10340     10350     10360
         ....|....|....|....|....|....|....|....|
AF126284 GAACTCGTATAAAGCACTAGTGGAACGGCCTGGGTATGCC
BR/P05   ........................................

                 10370     10380     10390     10400
         ....|....|....|....|....|....|....|....|
AF126284 CCCTTGAATCTTGAAGTCATGGTCATGAACACCCAGATCA
BR/P05   ........................................
```

```
                      10410     10420     10430     10440
          ....|....|....|....|....|....|....|....|
AF126284  TACCATCGGTTAAACGTGAATACATTACCTGCAGGTACCA
BR/P05    ........................................

                      10450     10460     10470     10480
          ....|....|....|....|....|....|....|....|
AF126284  CACCGTTGTTCCTTCACCGCAGATTAAATGTTGCGGAACT
BR/P05    ........................................

                      10490     10500     10510     10520
          ....|....|....|....|....|....|....|....|
AF126284  GTCGAATGCCCGAAAGGTGAAAAAGCAGACTATACCTGCA
BR/P05    ................................C.......

                      10530     10540     10550     10560
          ....|....|....|....|....|....|....|....|
AF126284  AGGTGTTCACTGGTGTGTACCCATTTCTGTGGGGAGGAGC
BR/P05    ........................................

                      10570     10580     10590     10600
          ....|....|....|....|....|....|....|....|
AF126284  ACAGTGTTTTTGCGACTCCGAAAACAGTCAGCTTAGCGAC
BR/P05    .......................................A

                      10610     10620     10630     10640
          ....|....|....|....|....|....|....|....|
AF126284  AAGTACGTCGAACTGTCAACAGATTGCGCCACAGACCATG
BR/P05    GCA.....................................

                      10650     10660     10670     10680
          ....|....|....|....|....|....|....|....|
AF126284  CCGAGGCGGTCAGAGTACACACGGCTTCGGTGAAATCACA
BR/P05    ........................................

                      10690     10700     10710     10720
          ....|....|....|....|....|....|....|....|
AF126284  GCTCCGAATAACCTACGGGAACTCCACAGCACAAGTAGAC
BR/P05    ........................................

                      10730     10740     10750     10760
          ....|....|....|....|....|....|....|....|
AF126284  GTATTTGTCAACGGTGTGACTCCAGCCAGGAGCAAAGACA
BR/P05    ........................................

                      10770     10780     10790     10800
          ....|....|....|....|....|....|....|....|
AF126284  TGAAATTGATAGCCGGCCCATTATCTACTACATTTTCCCC
BR/P05    ........................................

                      10810     10820     10830     10840
          ....|....|....|....|....|....|....|....|
AF126284  GTTTGATAATAAGGTCATTATATATCATGGGAAAGTCTAT
BR/P05    ........................................

                      10850     10860     10870     10880
          ....|....|....|....|....|....|....|....|
AF126284  AACTATGACTTCCCGGAATTTGGGGCCGGAACACCTGGAG
BR/P05    .......................C................

                      10890     10900     10910     10920
          ....|....|....|....|....|....|....|....|
AF126284  CTTTCGGAGATGTCCAAGCGTCATCCACCACCGGATCAGA
BR/P05    ........................................
```

```
                            10930     10940     10950     10960
         ....|....|....|....|....|....|....|....|
AF126284 TCTATTAGCAAACACAGCAATTCATTTGCAGAGGCCGGAA
BR/P05   ....CAT.................................

                            10970     10980     10990     11000
         ....|....|....|....|....|....|....|....|
AF126284 GCCAGAAACATACACGTCCCGTACACCCAAGCTCCAAGCG
BR/P05   ........................................

                            11010     11020     11030     11040
         ....|....|....|....|....|....|....|....|
AF126284 GGTTCGAATTCTGGAAGAATAACAGCGGTCAGCCTTTATC
BR/P05   ........................................

                            11050     11060     11070     11080
         ....|....|....|....|....|....|....|....|
AF126284 TGACACTGCCCCTTTCGGATGCAAAGTCAATGTCAACCCG
BR/P05   ..............................A.........

                            11090     11100     11110     11120
         ....|....|....|....|....|....|....|....|
AF126284 CTACGTGCAGACAAGTGTGCCGTGGGATCACTCCCGATAT
BR/P05   ........................................

                            11130     11140     11150     11160
         ....|....|....|....|....|....|....|....|
AF126284 CCGTGGATATACCGGACGCTGCATTTACACGCGTATCCGA
BR/P05   ........................................

                            11170     11180     11190     11200
         ....|....|....|....|....|....|....|....|
AF126284 G---CCCCTGCCATCACTGCTTAAGTGCACCGTTACTAGT
BR/P05   .GCC..TGCCATCAGTGCA.....................

                            11210     11220     11230     11240
         ....|....|....|....|....|....|....|....|
AF126284 TGCACATACTCTACAGACTATGGCGGAGTGCTCGTGTTGA
BR/P05   ..T...................A.................

                            11250     11260     11270     11280
         ....|....|....|....|....|....|....|....|
AF126284 CATACGAGTCGGATCGCGCGGGGCAATGCGCTGTACACTC
BR/P05   ........................................

                            11290     11300     11310     11320
         ....|....|....|....|....|....|....|....|
AF126284 GCATTCATCAACAGCGGTACTGCGAGACCCATCGGTATAC
BR/P05   ........................................

                            11330     11340     11350     11360
         ....|....|....|....|....|....|....|....|
AF126284 GTCGAGCAAAAAGGGGAGACTACACTTAAATTTAGTACGC
BR/P05   ...............C.......................G

                            11370     11380     11390     11400
         ....|....|....|....|....|....|....|....|
AF126284 GTTCCTTGCAGGCAGACTTCGAGGTATCGATGTGCGGAAC
BR/P05   C.......................................

                            11410     11420     11430     11440
         ....|....|....|....|....|....|....|....|
AF126284 GAGAACCACTTGCCATGCCCAATGTCAACCACCAACGGAA
BR/P05   ........................................
```

```
                   11450     11460     11470     11480
AF126284  CACGTAATGAACAGACCCCAGAAGTCGACTCCAGACTTCT
BR/P05    ........................................

                   11490     11500     11510     11520
AF126284  CCTCAGCGATATCCAAAACATCATGGAACTGGATTACAGC
BR/P05    ........................................

                   11530     11540     11550     11560
AF126284  GCTTATGGGGGGAATTTCCAGTATAGCTGCTATAGCCGCA
BR/P05    ..............C.........................

                   11570     11580     11590     11600
AF126284  ATTGTGCTGGTCATAGCATTAGTATTTACAGCACAACACA
BR/P05    ........................................

     3'NCR
                   11610     11620     11630     11640
AF126284  GATGAATACAAACTTATTACATTACGTATGTATTCGATGT
BR/P05    ........................................

                   11650     11660     11670     11680
AF126284  TATATGTATAACAAT-GAACGTAAACTCGATGTACTTCCG
BR/P05    ..............CA..C.....................

                   11690     11700     11710     11720
AF126284  AGGATGTG-GGTGCATAATGCCATACAGCGGTCCACTTAT
BR/P05    ........T...............................

                   11730     11740     11750     11760
AF126284  CATGATATAGTTTTATAGTTACCACCGGTGAAACTCGATG
BR/P05    ...C......-.............................

                   11770     11780     11790     11800
AF126284  TATTTCCGAGGAAGTGTGGTGCATAATGCCACACACCGGT
BR/P05    ........................................

                   11810     11820     11830     11840
AF126284  TGAATTTAATAGTTTTAGTCAAGACTGAGAAAACTCGATG
BR/P05    ........................................

                   11850     11860     11870     11880
AF126284  TACTTCCGAGGA--TGTGGTGCATAATGTCACACATCAGT
BR/P05    ............AG....A......T..............

                   11890     11900     11910     11920
AF126284  CTTATCATATTAGTTCAAA-GGCAATATTACCCCTGAATA
BR/P05    ...................G....................

                   11930     11940     11950     11960
AF126284  GTAACAAAACTAGAAAATCGTCAACCACCA-CGGATACCG
BR/P05    ........................T.....C.........
```

```
                                  11970               11980               11990               12000
              . . . . | . . . . . | . . . . . | . . . . . | . . . . . | . . . . . | . . . . . | . . . . . |
AF126284      G G A T C G G T G T C C T A C T A C G G T A G T T G A A A A T A A C T T T A T A
BR/P05        . . . . . . . . . . . . . . . . . . . . . . . . . . . . . . . . . . . . . . . .

                                  12010               12020               12030               12040
              . . . . | . . . . . | . . . . . | . . . . . | . . . . . | . . . . . | . . . . . | . . . . . |
AF126284      G A A T T T T A A A A T T T T C T T T A T T A A A A T C T T T T G T T T T T C T
BR/P05        . . . . . . . . . . . . . . . . . . . . . . . . . . . . . . . . . . . . . . . .

                                  12050               12060               12070
              . . . . | . . . . . | . . . . . | . . . . . | . . . . . | . . . . . | . . .
AF126284      T T T A T T A T T T C A A A A T T T T G T T T T T A A T A T T T C
BR/P05        . . . . . . . . . . . . . . . . . . . . . . . . . . . . . . . . .
```

ORF1

nsP1

```
                  10        20        30        40
AF126284  MEKPTVHVDVDPQSPFVLQLQKSFPQFEIVAQQVTPNDHA
BR/P05    ...............................R........

                  50        60        70        80
AF126284  NARAFSHLASKLIEHEIPTSVTILDIGSAPARRMYSEHKY
BR/P05    ........................................

                  90       100       110       120
AF126284  HCVCPMRSPEDPDRLMNYASRLADKAGEITNKRLHDKLAD
BR/P05    ........................................

                 130       140       150       160
AF126284  LKSVLESPDAETGTICFHNDVICRTTAEVSVMQNVYINAP
BR/P05    .....................T...........D...D..

                 170       180       190       200
AF126284  STIYHQALKGVRKLYWIGFDTTQFMFSSMAGSYPSYNTNW
BR/P05    ........................................

                 210       220       230       240
AF126284  ADERVLEARNIGLCSTKLREGTMGKLSTFRKKALKPGTNV
BR/P05    ..................T........IL.........D.

                 250       260       270       280
AF126284  YFSVGSTLYPENRADLQSWHLPSVFHLKGKQSFTCRCDTA
BR/P05    ........................................

                 290       300       310       320
AF126284  VNCEGYVVKKITISPGITGRVNRYTVTNNSEGFLLCKITD
BR/P05    ......................G.A...............

                 330       340       350       360
AF126284  TVKGERVSFPVCTYIPPSICDQMTGILATDIQPEDAQKLL
BR/P05    ........................................

                 370       380       390       400
AF126284  VGLNQRIVVNGKTNRNTNTMQNYLLPAVATGLSKWAKERK
BR/P05    ........................................

                 410       420       430       440
AF126284  ADCSDEKPLNVRERKLAFGCLWAFKTKKIHSFYRPPGTQT
BR/P05    ..AD........G...........................

                 450       460       470       480
AF126284  IVKVAAEFSAFPMSSVWTTSLPMSLRQKVKLLLVKKTNKP
BR/P05    ........................................

                 490       500       510       520
AF126284  VVTITDTAVKNAQEAYNEAVETAEAEEKAKALPPLKPTAP
BR/P05    .......E................................
```

nsP2 →

```
                              530                 540                 550                 560
AF126284   P V A E D V K C E V T D L V D D A G A A L V E T P R G K I K I I P Q E G D V R I
BR/P05     . . . . . . . . . . . . . . . . . . . . . . . . . . . . . . . . . . . . . . . .

                              570                 580                 590                 600
AF126284   G S Y T V I S P A A V L R N Q Q L E P I H E L A E Q V K I I T H G G R T G R Y S
BR/P05     . . . . . . . . . . . . . . . . . . . . . . . . . . . . . . . . . . . . . . . .

                              610                 620                 630                 640
AF126284   V E P Y D A K V L L P T G C P M S W Q H F A A L S E S A T L V Y N E R E F L N R
BR/P05     . . . . . . . . . . . . . S A . . . . . . . . . . . . . . . . . . . . . . . . .

                              650                 660                 670                 680
AF126284   K L H H I A T K G A A K N T E E E Q Y K V C K A K D T D H E Y V Y D V D A R K C
BR/P05     R . . . . . . . . . . . . . . . . . . . . . . . . . . . . . . . . . . . . . . .

                              690                 700                 710                 720
AF126284   V K R E H A Q G L V L V G E L T N P P Y H E L A Y E G L R T R P A A P Y H I E T
BR/P05     . . . . . . . . . . . . . . . . . . . . . . . . . . . . . . . . . . . . . . . .

                              730                 740                 750                 760
AF126284   L G V I G T P G S G K S A I I K S T V T L K D L V T S G K K E N C K E I E N D V
BR/P05     . . . . . . . . . . . . . . . . . . . . . . . . . . . . . . . . . . . . . D . .

                              770                 780                 790                 800
AF126284   Q K M R G M T I A T R T V D S V L L N G W K K A V D V L Y V D E A F A C H A G T
BR/P05     . . . . . . . . . . . . . . . . . . . . . . . . . . . . . . . . . . . . . . . .

                              810                 820                 830                 840
AF126284   L M A L I A I V K P R R K V V L C G D P K Q W P F F N L M Q L K V N F N N P E R
BR/P05     . . . . . . . . . . . . . . . . . . . . . . C G . . . . . . . . . . . . . . . .

                              850                 860                 870                 880
AF126284   D L C T S T H Y K Y I S R R C T Q P V T A I V S T L H Y D G K M R T T N P C K R
BR/P05     . . . . . . . . . . . . . . . . . . . . . . . . . . . . . . . . . . . . . . . .

                              890                 900                 910                 920
AF126284   A I E I D V N G S T K P K K G D I V L T C F R G W V K Q G Q I D Y P G P G G H D
BR/P05     . . . . . . . . . . . . . . . . . . . . . . . . . . . . L . . . . . . H E V M T

                              930                 940                 950                 960
AF126284   R A A S Q G L T R R G V Y A V R Q K V N E N P L Y A E K S E H V N V L L T R T E
BR/P05     A . . . . . . . . K . . . . . . . . . . . . . . . . . . . . . . . . . . . . . .

                              970                 980                 990                1000
AF126284   D R I V W K T L Q G D P W I K Y L T N V P K G N F T A T L E E W Q A E H E D I M
BR/P05     . . . . . . . . . . . . . . . . . . . . . . . . . . . . . . . . . . . . . . . .

                             1010                1020                1030                1040
AF126284   K A I N S T S T V S D P F A S K V N T C W A K A I I P I L R T A G I E L T F E Q
BR/P05     . . . . . . . . . . . . . . . . . . . . . . . . . . . . . . . . . . . . . . . .
```

```
                    1050      1060      1070      1080
AF126284   WEDLFPQFRNDQPYSVMYALDVICTKMFGMDLSSGIFSRP
BR/P05     ...............AI.....V.................

                    1090      1100      1110      1120
AF126284   EIPLTFHPADVGRVRAHWDNSPGGQKFGYNKAVIPT-CKK
BR/P05     ..........I.......................ANLA..

                    1130      1140      1150      1160
AF126284   YPVYLRAGKGDQILPIYGRVSVPSARNNLVPLNRNLPHSL
BR/P05     ...............V........................

                    1170      1180      1190      1200
AF126284   TASLQKKEAAPLHKFLNQLPGHSMLLVSKETCYCVSKRIT
BR/P05     ........................................

                    1210      1220      1230      1240
AF126284   WVAPLGVRGADHNHDLHFGFPPLSRYDLVVVNMGQPYRFH
BR/P05     .................................DT.....

                    1250      1260      1270      1280
AF126284   HYQQCEEHAGLMRTLARSALNCLKPGGTLALKAYGFADSN
BR/P05     ........................................

                    1290      1300      1310      1320
AF126284   SEDVVLSLARKFVRASAVRPSCTQFNTEMFFVFRQLDNDR
BR/P05     ..............V.........................

                    1330      1340 nsP3 →    1350      1360
AF126284   ERQFTQHHLNLAVSNIFDNYKDGSGAAPSYRVKRMNIADC
BR/P05     ........................................

                    1370      1380      1390      1400
AF126284   TEEAVVNAANARGKPGDGVCRAIFKKWPKSFENATTEVET
BR/P05     ...........A..........................G.

                    1410      1420      1430      1440
AF126284   AVMKPCHNKVVIHAVGPDFRKYTLEEATKLLQNAYHDVAK
BR/P05     .................N......................

                    1450      1460      1470      1480
AF126284   IVNEKGISSVAIPLLSTGIYAAGADRLDLSLRCLFTALDR
BR/P05     .................S......................

                    1490      1500      1510      1520
AF126284   TDADVTIYCLDKKWEQRIADAIRMREQVTELKDPDIEIDE
BR/P05     ..........................H.............

                    1530      1540      1550      1560
AF126284   GLTRVHPDSCLKDHIGYSTQYGKLYSYFEGTKFHQTAKDI
BR/P05     ...................SM.............R.....
```

```
                                 1570                1580                1590                1600
AF126284   A E I R A L F P D V Q A A N E Q I C L Y T L G E P M E S I R E K C P V E D S P A
BR/P05     . . . . . . . . . . . . . . . . . . . . . . . . . . . . . . . . . . . . . . . .

                                 1610                1620                1630                1640
AF126284   S A P P K T I P C L C M Y A M T A E R I C R V R S N S V T N I T V C S S F P L P
BR/P05     . . . . . . . . . . . . . . . . . . . . . . L . . . . . . . . . . . . . . . . .

                                 1650                1660                1670                1680
AF126284   K Y R I K N V Q K I Q C T K V V L F N P D V P P Y I P A R V Y I N K D E P P V T
BR/P05     . . . . . . . . . V . . . . . . . . . . . . . . . . . . . . . . . . . . . . . .

                                 1690                1700                1710                1720
AF126284   P H T D S P P D T C S S R L S L T P T L S N A E S D I V S L T F S E I D S E L S
BR/P05     . . . . . . . . . . . . . . . . . . . . . . . . . . . . . . . . . . . . . . . .

                                 1730                1740                1750                1760
AF126284   S L N E P A R H V M I S S F K L R Y T A I Q A L P Q K L S W M R E D R T P R Q P
BR/P05     . . . . . . E A R N D I L V Q A E V H C D P G F T S E A E L D E R G S . . . . .

                                 1770                1780                1790                1800
AF126284   P P V P P P R P K R A A K L S R L A N Q - - - - - - - - - - - - - - - - - - - -
BR/P05     . . . . . . . . . . . . . . . . . . . H A E S D I V S L T F S E I D S E L S S L

                                 1810                1820                1830                1840
AF126284   - - - - - - - - - - - - - - - - - - - - - - - - - - - - - - - - - - - - - - - -
BR/P05     N E P A E A R N D I L V Q A E V H C D P G F T S E A E L D E R G S T P R Q P P P

                                 1850                1860                1870                1880
AF126284   - - - - - - - - - - - - - - - - - - L N E L R R H A T I S S V Q A E V H Y N S G
BR/P05     V P P P R P K R A A K L S R L A N Q . . . P T E A C D D I L . . . . . . . . . .

                                 1890                1900                1910                1920
AF126284   F T P E A E L N E R G S I L R K P P P V P P L R P K Q T T N L S R L A N Q L S M
BR/P05     . . . . . . . . . G . . . . . . . . . . . . . . . . . . . . . . . . . . . . . .

                                 1930                1940                1950                1960
AF126284   P I T F G D F A E G E L D R L L T P S P T P T F G D F S Q E E M D R F F G N R Q
BR/P05     . . . . . . . . . . . . . . . . . . . . . . . . . . . . . . . . . . . . . . . .

                    nsP4 →
                                 1970                1980                1990                2000
AF126284   Y * L T G V G G Y I F S S D T G P G H L Q Q K S V I Q N S T T E I L I E R S R L
BR/P05     . R . . . . . . F . . . . . . . . . . . . . . . . M . . . . . . . . . . . . . .

                                 2010                2020                2030                2040
AF126284   E K I H A P V L D L Q K E E M L K C R Y Q M S P T V A N K S R Y Q S R K V E N M
BR/P05     . . . . . . . . . . . . . . . . . . . . . . . . . . . . . . . . . . . . . . . .

                                 2050                2060                2070                2080
AF126284   K A V T T G R L L D G L K M Y V T P D V E A E C Y K Y T Y P K P M Y S A S V P D
BR/P05     . . . . . V . . . . . . . . . . . . . . . . . . . . . . . . . . . . . . . . . .
```

```
                                 2090                2100                2110                2120
AF126284  R F V S P E V A V A V C N N F F H E N Y P T V A S Y Q I T D E Y D A Y L D M V E
BR/P05    . . . . . . . . . . . . . . . . . . . . . . . . . . . . . . . . . . . . . . L D

                                 2130                2140                2150                2160
AF126284  G S V S C L D T A T F C P A K L R S F P K T H S Y L E P T L R S A V P S A F Q N
BR/P05    . . . . . . . . . . . . . . . . - . . . . . . . . . . . . . . . . . . . . . . .

                                 2170                2180                2190                2200
AF126284  T L Q N V L S A A T K R N C N V T Q M R E L P V L D S A V F N V E C F K K Y A C
BR/P05    . . . . . . . . . . . . . . . . . . . . . . . . . . . . . L . . . . . . . . . .

                                 2210                2220                2230                2240
AF126284  N T D Y W E E F K E K P I R I T T E C V T S Y V A R L K G P E A A A L F A K T H
BR/P05    . . . . . . . . . . . . . . . . . . . . . . . . . . . . . . K . . . . . . . . .

                                 2250                2260                2270                2280
AF126284  Q L V P L Q E V P M D R F V M D M K R D V K V T P G T K H T E E R P K V Q V I Q
BR/P05    R . . . . . . . . . . . . . . . . . . . . . . . . . . . . . . . . . . . . . . .

                                 2290                2300                2310                2320
AF126284  A A E P L A T A Y L C G I H R E L V R R L T A V L L P N I H T L F D M S A E D F
BR/P05    . . . . . . . . . . . . . . . . . . . . . . . . . . . . . . . . . . . . . . . .

                                 2330                2340                2350                2360
AF126284  D A I I A A N F S Y V H P V L E T D I G S F D K S Q D D S L A L T A L M I L E D
BR/P05    . . . . . . . . . . G D . . . . . . . A . . . . . . . . . . . . . . . . . . . .

                                 2370                2380                2390                2400
AF126284  L G V D D R L M D L I E C A F G E I T S V H L P T A T T F K F G A M M K S G M F
BR/P05    . . . . . . . . . . . . . . . . . . . . . . . . . . . R . . . . . . . . . . . .

                                 2410                2420                2430                2440
AF126284  L T L F V N T V L N V V I A S R V L E Q R L R D S K C A A F I G D D N I I H G V
BR/P05    . . . . . . . . . . . . . . . . . . . . . . . . . . . . . . . . . . . . . . . .

                                 2450                2460                2470                2480
AF126284  V S D K I M A D R C A T W M N M E V K I I D A V I G I K A P Y F C G G F I L E D
BR/P05    . . . . . . . . . . . . . . . . . . . . . . T . . . . . . . . . . . . . . . . .

                                 2490                2500                2510                2520
AF126284  Q V T H T A C R V S D P L K R L F K L G K P L P V D D E Q D H D R R R A L E D E
BR/P05    . . . . . . . . . . . . . . . . . . . . . . . . . . . . . . . . . . . . . . . .

                                 2530                2540                2550                2560
AF126284  T R A W F R V G I Q G E L L K A V E S R Y E V Q E V Q P V L L A L A T F S R S D
BR/P05    . . . . . . . . . . . . . . . . . . . . . . . . . . . . . . . . . . . . . . . .

                                 2570
AF126284  K A F K A L R G S P R H L Y G G P K *
BR/P05    . . . . . . . . . . . . . . . . . . *
```

ORF2

```
C →
                   10        20        30        40
AF126284  MNSVFYNPFGRGAYAQPPIAWRPRRRAAPAPRPSGLTTQI
BR/P05    ..........................R.............

                   50        60        70        80
AF126284  QQLTRAVRALVLDNATRRQRPAPRTRPRKPKTQKPKPKKQ
BR/P05    .......G.................A..............

                   90       100       110       120
AF126284  NQKPPQQQKKGKNQPQQPKKPKPGKRQRTALKFEADRTFV
BR/P05    .......................................A

                  130       140       150       160
AF126284  GKNEDGKIMGYAVAMEGKVIKPLHVKGTIDHPALAKLKFT
BR/P05    V....................................N..

                  170       180       190       200
AF126284  KSSSYDMEFAKLPTEMKSDAFGYTTEHPEVFYNWHHGAVQ
BR/P05    ......T..............R.......G..........

                  210       220       230       240
AF126284  FSGGRFTIPTGVGGPGDSGRPILDNSGKVVAIVLGGANEV
BR/P05    ...........A...........................G

                  250       260   E3 →270       280
AF126284  PGTALSVVTWNKKGAAIKTTHEDTVEWSRAITAMCILQNV
BR/P05    AR..........................A...........

                  290       300       310       320
AF126284  TFPCDRPPTCYNRNPDLTLTMLETNVNHPSYDVLLDAALR
BR/P05    ........................................

              E2 →330       340       350       360
AF126284  CPTRRHVRSTPTDDFTLTAPYLGLCHRCKTMEPCYSPIKI
BR/P05    ..............L...........T.............

                  370       380       390       400
AF126284  EKVWDDADDGVLRIQVSAQLGYNRAGTAASARLRFMGGGV
BR/P05    ........................................

                  410       420       430       440
AF126284  PPEIQEGAIADFKVFTSKPCLHLSHKGYFVIVKCPPGDSI
BR/P05    ........................................

                  450       460       470       480
AF126284  TTSLKVHGSDQTCTIPMRVGYKFVGREKYTLPPMHGTQIP
BR/P05    .........................K..............

                  490       500       510       520
AF126284  CLTYERTREKSAGYVTMHRPGQQSITMLMEESGGEVYVQP
BR/P05    .......P................................
```

```
                 530       540       550       560
AF126284  TSGRNVTYECKCGDFKTGTVTARTKIDGCTERKQCIAISA
BR/P05    .....................................YF.

                 570       580       590       600
AF126284  DHVKWVFNSPDLIRHTDHTAQGKLHIPFPLQQAQCTVPLA
BR/P05    ........................................

                 610       620       630       640
AF126284  HLPGVKHAYRSMSLTLHAEHPTLLTTRHLGENPQPTAEWI
BR/P05    ...R..Y.................................

                 650       660       670       680
AF126284  VGSVTRNFSITIQGFEYTWGNQKPVRVYAQESAPGNPHGW
BR/P05    .....................RR.................

                 690       700       710       720
AF126284  PHEIVRHYYHLYPFYTVTVLSGMGLAICAGLVISILCCCK
BR/P05    ........................................

                                         6K ->
                 730       740       750       760
AF126284  ARRDCLTPYQLAPNATVPFLVTLCCCFQRTSADEFTDTMG
BR/P05    .............................A..........

                 770       780       790       800
AF126284  YLWQHSQTMFWIQLVIPLAAVITLVRCCSCCLPFLLVASP
BR/P05    ......................................GV

             E1 ->
                 810       820       830       840
AF126284  -PNKADAYEHTITVPNAPLNSYKALVERPGYAPLNLEVMV
BR/P05    LLT.....................................

                 850       860       870       880
AF126284  MNTQIIPSVKREYITCRYHTVVPSPQIKCCGTVECPKGEK
BR/P05    ........................................

                 890       900       910       920
AF126284  ADYTCKVFTGVYPFLWGGAQCFCDSENSQLSDKYVELSTD
BR/P05    ...............................EA.......

                 930       940       950       960
AF126284  CATDHAEAVRVHTASVKSQLRITYGNSTAQVDVFVNGVTP
BR/P05    ........................................

                 970       980       990      1000
AF126284  ARSKDMKLIAGPLSTTFSPFDNKVIIYHGKVYNYDFPEFG
BR/P05    ........................................

                1010      1020      1030      1040
AF126284  AGTPGAFGDVQASSTTGSDLLANTAIHLQRPEARNIHVPY
BR/P05    ....................H...................
```

```
                                   1050                1060                1070                1080
          . . . . | . . . . | . . . . | . . . . | . . . . | . . . . | . . . . | . . . . |
AF126284  T Q A P S G F E F W K N N S G Q P L S D T A P F G C K V N V N P L R A D K C A V
BR/P05    . . . . . . . . . . . . . . . . . . . . . . . . . . . . K . . . . . . . . . . .

                                   1090                1100                1110                1120
          . . . . | . . . . | . . . . | . . . . | . . . . | . . . . | . . . . | . . . . |
AF126284  G S L P I S V D I P D A A F T R V S E - P L P S L L K C T V T S C T Y S T D Y G
BR/P05    . . . . . . . . . . . . . . . . . . . A . A I . A . . . . . . . . . . . . . . D

                                   1130                1140                1150                1160
          . . . . | . . . . | . . . . | . . . . | . . . . | . . . . | . . . . | . . . . |
AF126284  G V L V L T Y E S D R A G Q C A V H S H S S T A V L R D P S V Y V E Q K G E T T
BR/P05    . . . . . . . . . . . . . . . . . . . . . . . . . . . . . . . . . . . . . Q . .

                                   1170                1180                1190                1200
          . . . . | . . . . | . . . . | . . . . | . . . . | . . . . | . . . . | . . . . |
AF126284  L K F S T R S L Q A D F E V S M C G T R T T C H A Q C Q P P T E H V M N R P Q K
BR/P05    . . . . . A . . . . . . . . . . . . . . . . . . . . . . . . . . . . . . . . . .

                                   1210                1220                1230                1240
          . . . . | . . . . | . . . . | . . . . | . . . . | . . . . | . . . . | . . . . |
AF126284  S T P D F S S A I S K T S W N W I T A L M G G I S S I A A I A A I V L V I A L V
BR/P05    . . . . . . . . . . . . . . . . . . . . . . . T . . . . . . . . . . . . . . . .


          . . . . | . .
AF126284  F T A Q H R *
BR/P05    . . . . . . *
```

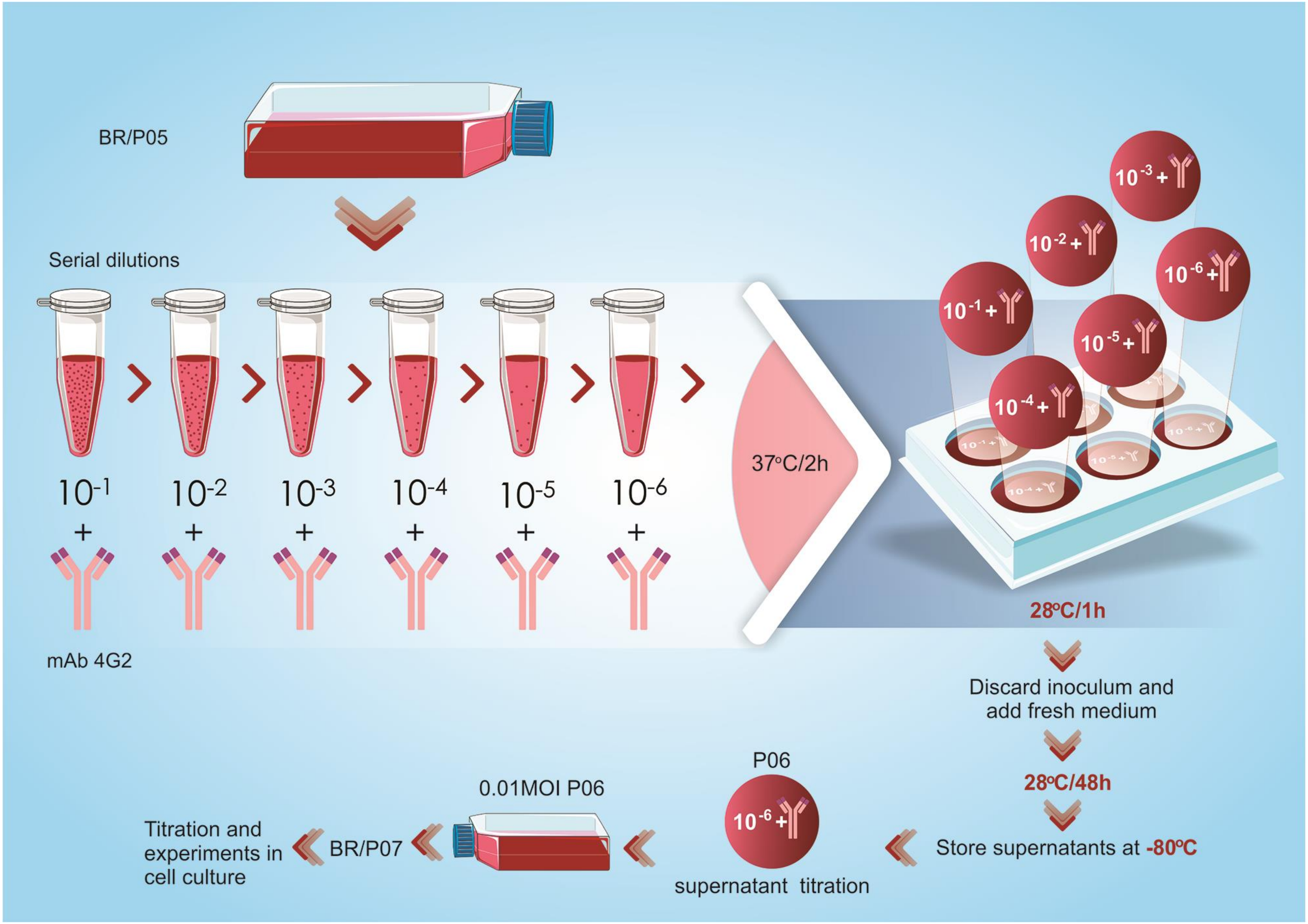

Figure S1. Workflow depicting the protocol used for DENV neutralization and production of the AURAV viral stock (BR/P07) from BR/P05. Different dilutions (10^{-1}-10^{-6}) of the supernatant of BR/P05 were incubated at 37 ºC with an anti-flavivirus monoclonal antibody (4G2) for 2 h. This mixture was incubated with C6/36 cells (3.5×10^5 cells/well, seeded the day before in a 6-well plate) for 1 h at 28 ºC. The inoculum was discarded, and the cell monolayer washed once with sterile PBS, after which 3 ml/well of medium added. The supernatants were collected at two days post-infection, aliquoted and stored at -80 ºC. The supernatant of the infection (P06) that was carried out with the least amount of virus (10^{-6}) was titrated and used to infect C6/36 cells at a multiplicity of infection (MOI) of 0.01 to produce the viral stock (BR/P07) for biological characterization experiments. Figure by Wagner Nagib de Souza Birbeire.

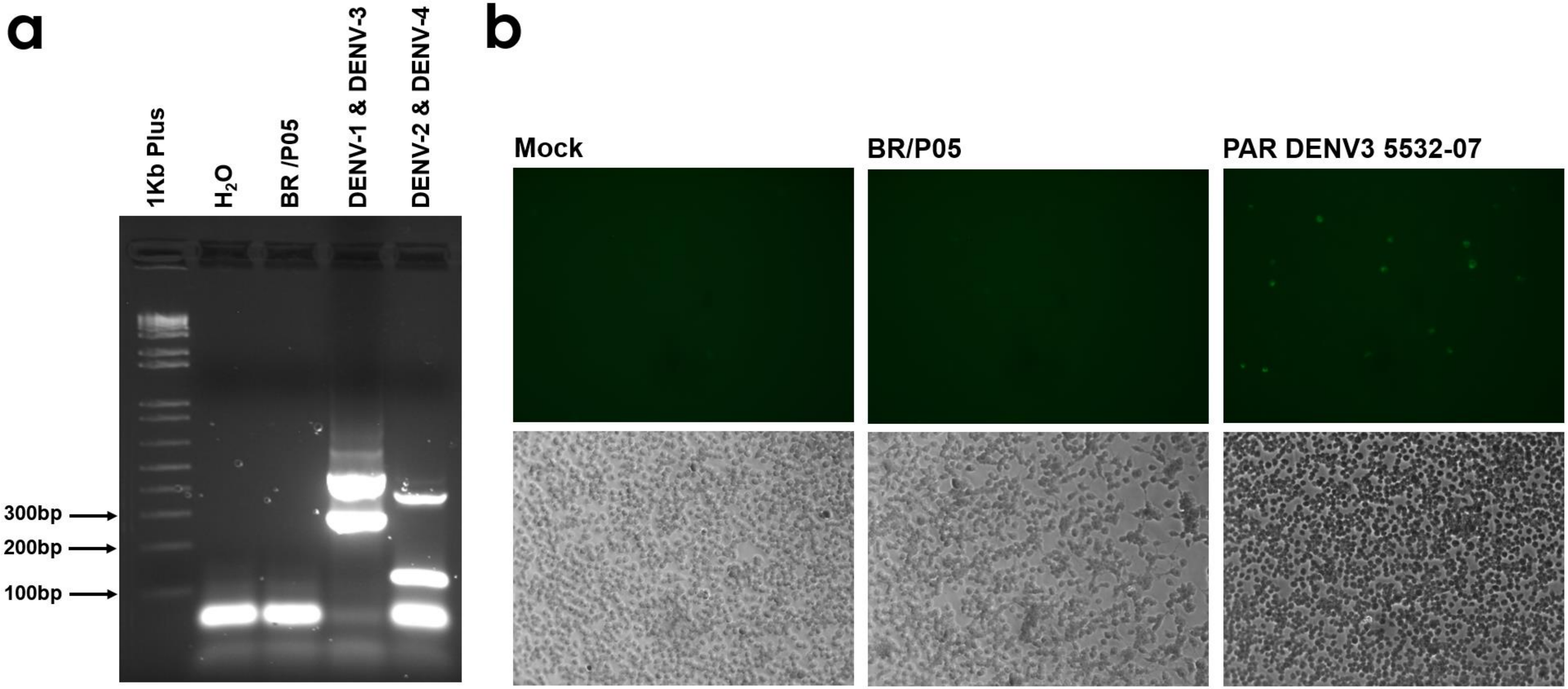

Figure S2. DENV detection in BR/P05. **a** One-step RT-PCR for DENV serotyping. RNA extracted from the supernatant of BR/P05 was used as the template. Water was used as negative control, and RNAs extracted from DENV-1, -2, -3 and 4 were used as positive controls. **b** IFA of C6/36 cells infected with the supernatant of BR/P05 at 2 days post-infection. Cells were fixed with 3% paraformaldehyde, permeabilized with 0.5% triton and incubated with an anti-flavivirus monoclonal antibody (4G2) followed by a goat anti-mouse IgG (H+L), Alexa Fluor 488 conjugate. Images were obtained at 200x magnification using a Nikon Eclipse TE300 inverted microscope attached to a CoolSNAP-ProCf camera and visualized and processed using Image-Pro Plus version 4.5.1.22 software.

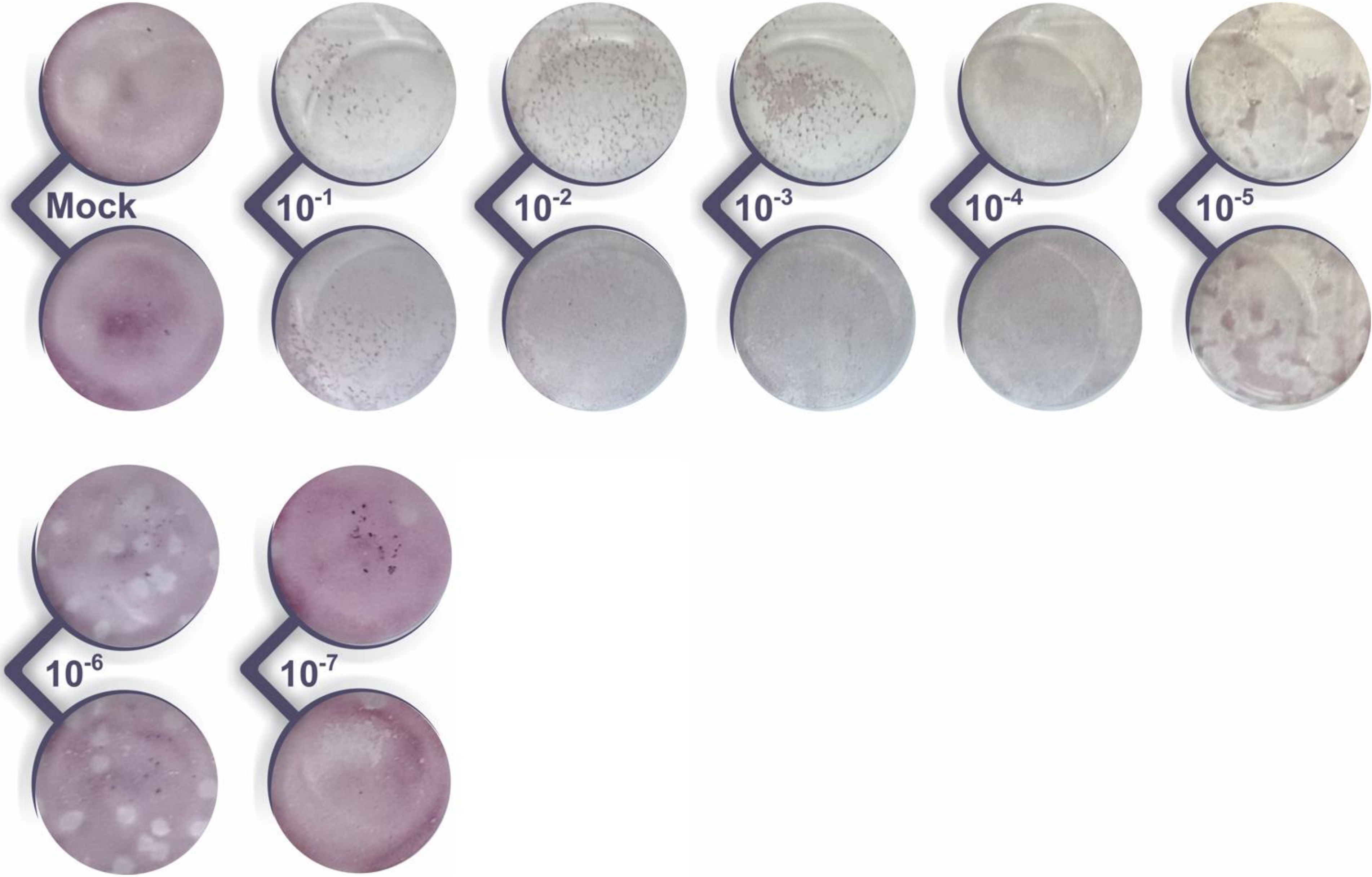

Figure S3. Result of BR/P07 titration by plaque assay in C6/36 cells.

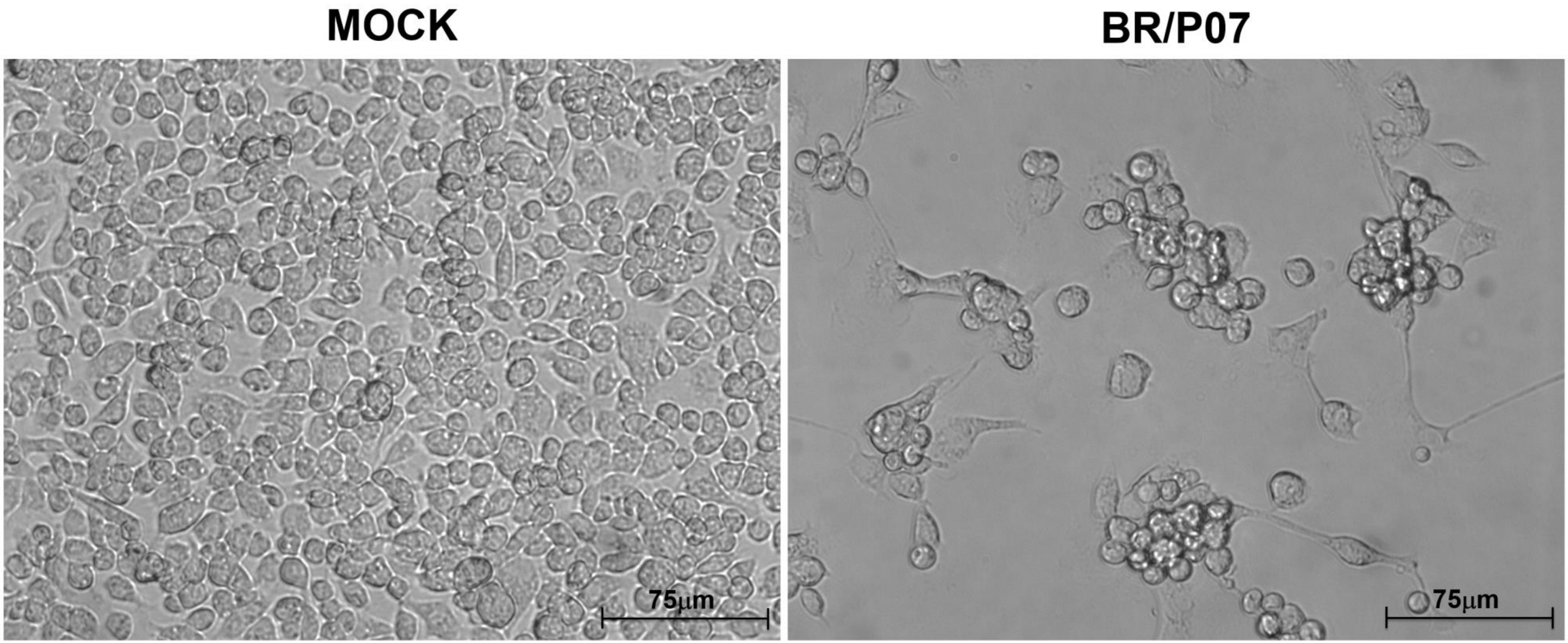

Figure S4. Brightfield images of C6/36 cells infected or not (mock) with 0.1 MOI of BR/P07 at 3 days post-infection. Images were obtained at 400x magnification using a Nikon Eclipse TE300 inverted microscope attached to a CoolSNAP-ProCf camera and visualized and processed using Image-Pro Plus version 4.5.1.22 software.

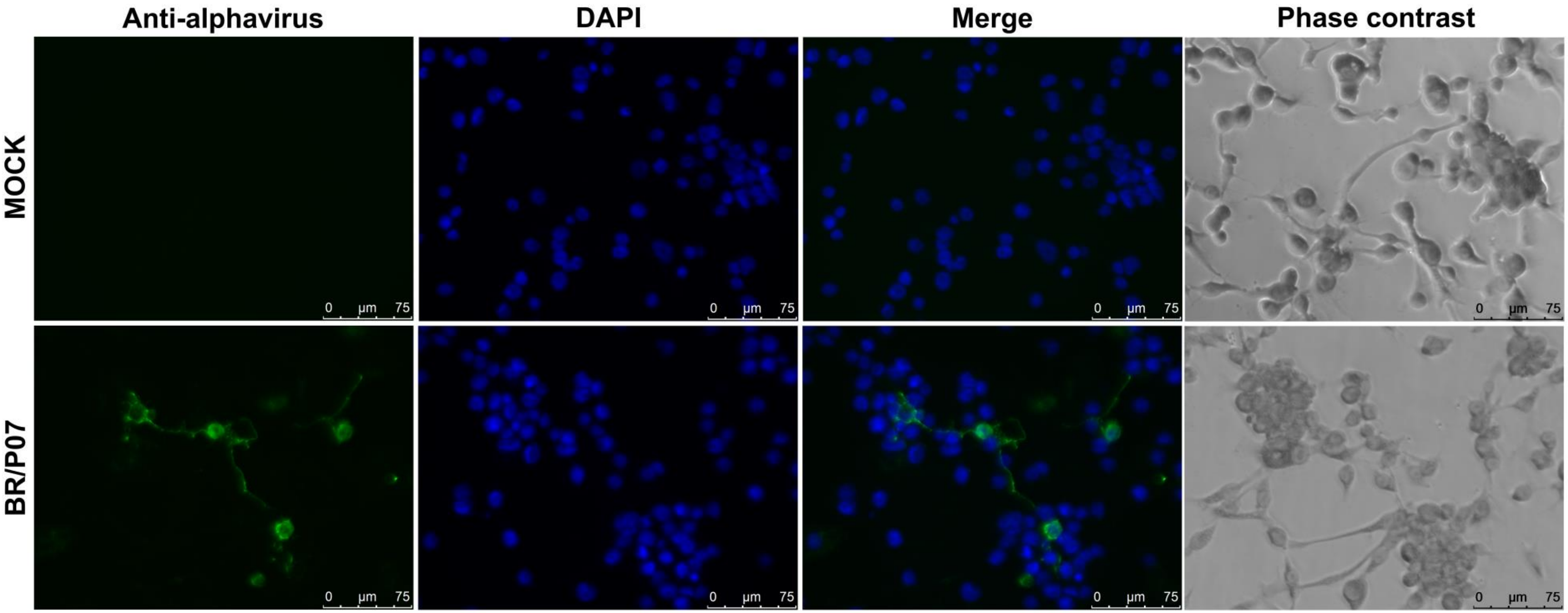

Figure S5. IFA of AP-61 cells infected or not (mock) with 1 MOI of BR/P07. At 3 days post-infection, the cell monolayer was fixed and permeabilized with methanol:acetone (1:1) at -20 ^{o}C and then incubated with an anti-alphavirus monoclonal antibody (mAb) clone 1A4B-6 followed by a goat anti-mouse IgG (H+L), Alexa Fluor 488 conjugate. Images were obtained using a Leica DMI 6000B inverted microscope attached to a Leica DFC365 FX camera and visualized and processed using Leica Application Suite Advanced Fluorescence 3.1.0 software.

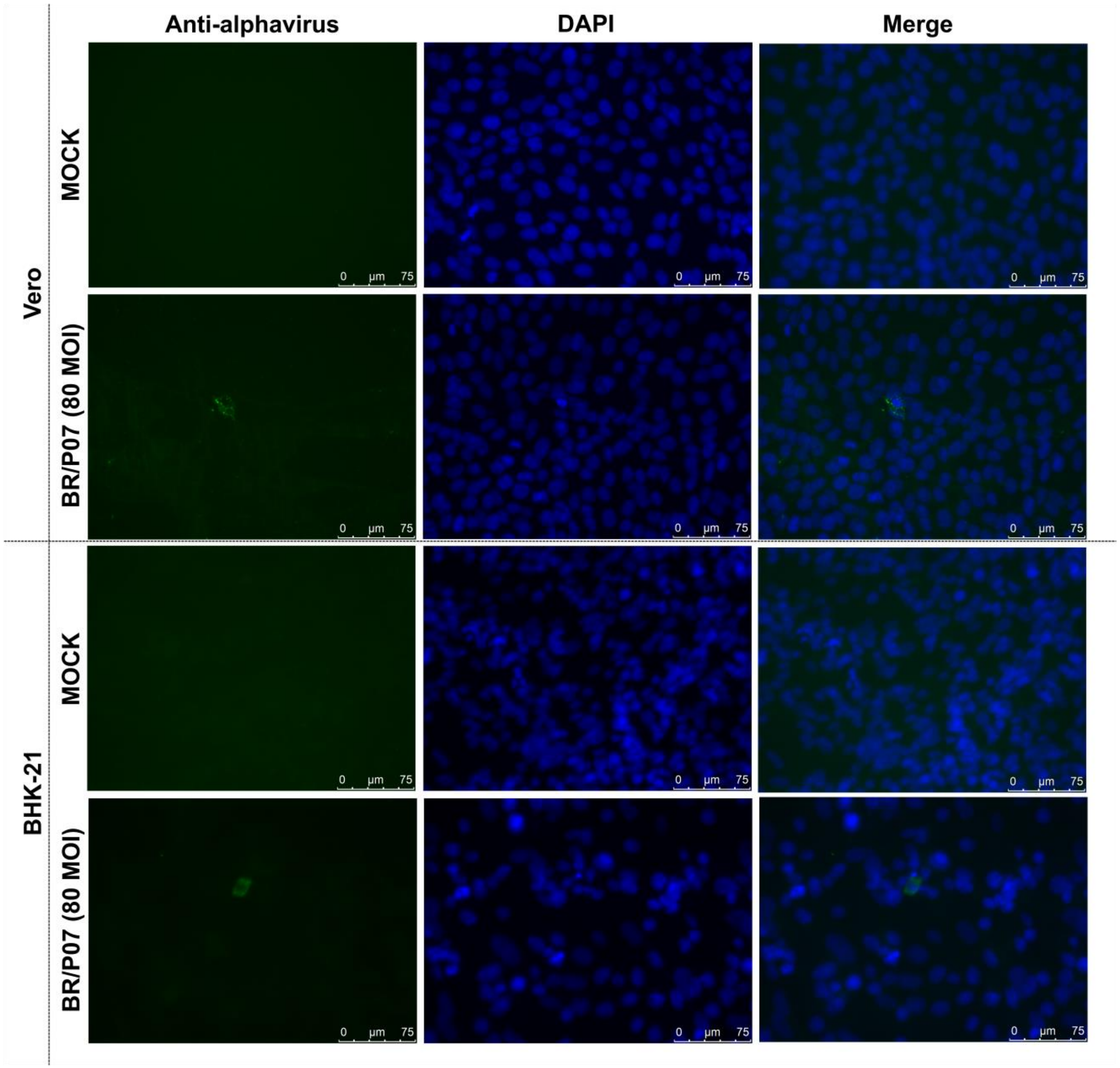

Figure S6. IFA of Vero and BHK-21 cells infected or not (mock) with 80 MOI of BR/P07. At 3 days post-infection, cell monolayers were fixed and permeabilized with methanol:acetone (1:1) at -20 ^{o}C and then incubated with an anti-alphavirus monoclonal antibody (mAb) clone 1A4B-6 followed by a goat anti-mouse IgG (H+L), Alexa Fluor 488 conjugate. Images were obtained using a Leica DMI 6000B inverted microscope attached to a Leica DFC365 FX camera and visualized and processed using Leica Application Suite Advanced Fluorescence 3.1.0 software.

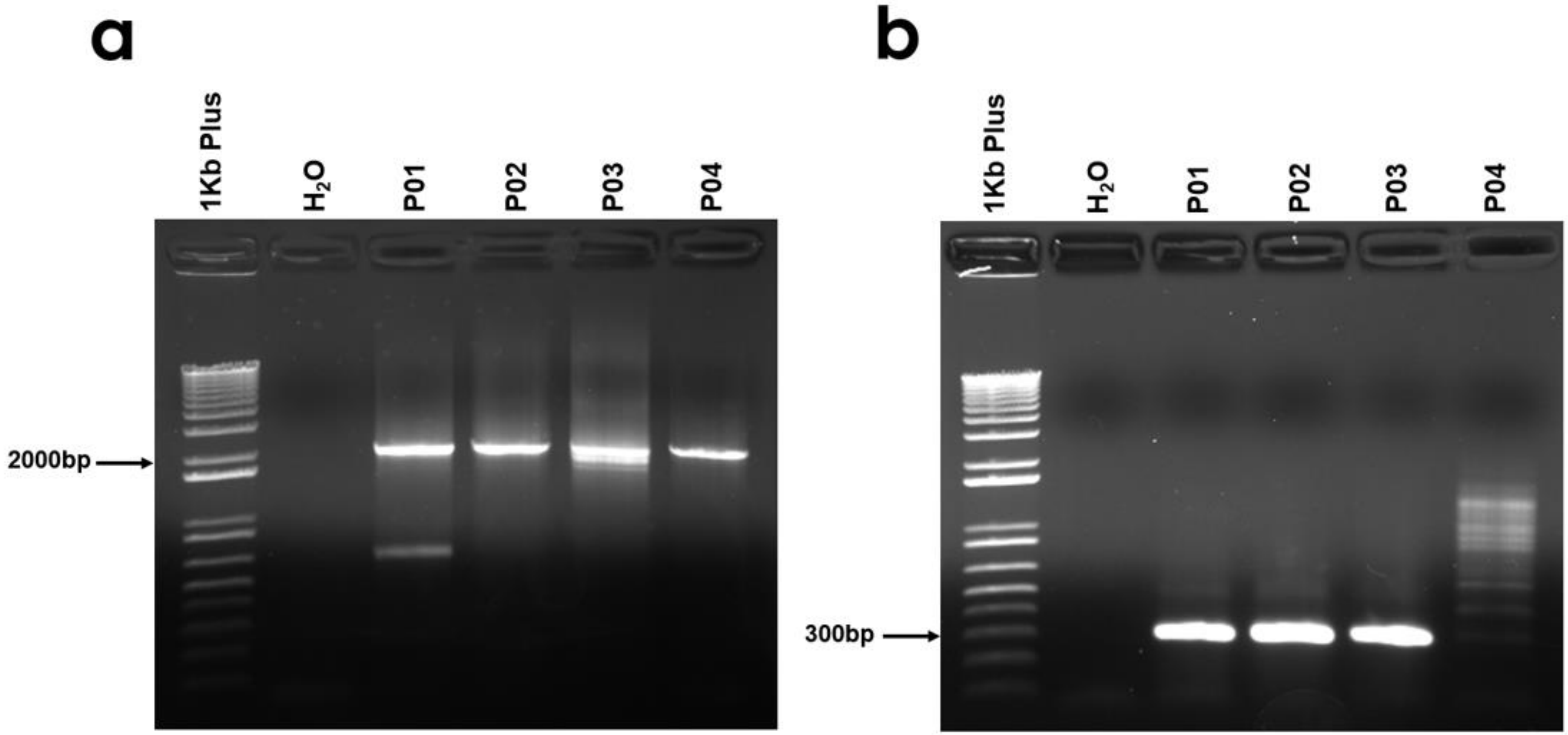

Figure S7. RT-PCR of P01-P04 for **a** AURAV and **b** DENV. RT-PCR was carried out following standard protocols. The primers used for AURAV testing were NsP3F and NsP3R (see Supplementary Table 2 for primer sequence). The primers used for DENV testing were D1 (5'-TCAATATGCTGAAACGCGCGAGAAACCG-3') and TS3 (5'-TAACATCATCATGAGACAGAGC-3'). The expected amplicon size for AURAV and DENV is 1995 bp and 290 bp, respectively.

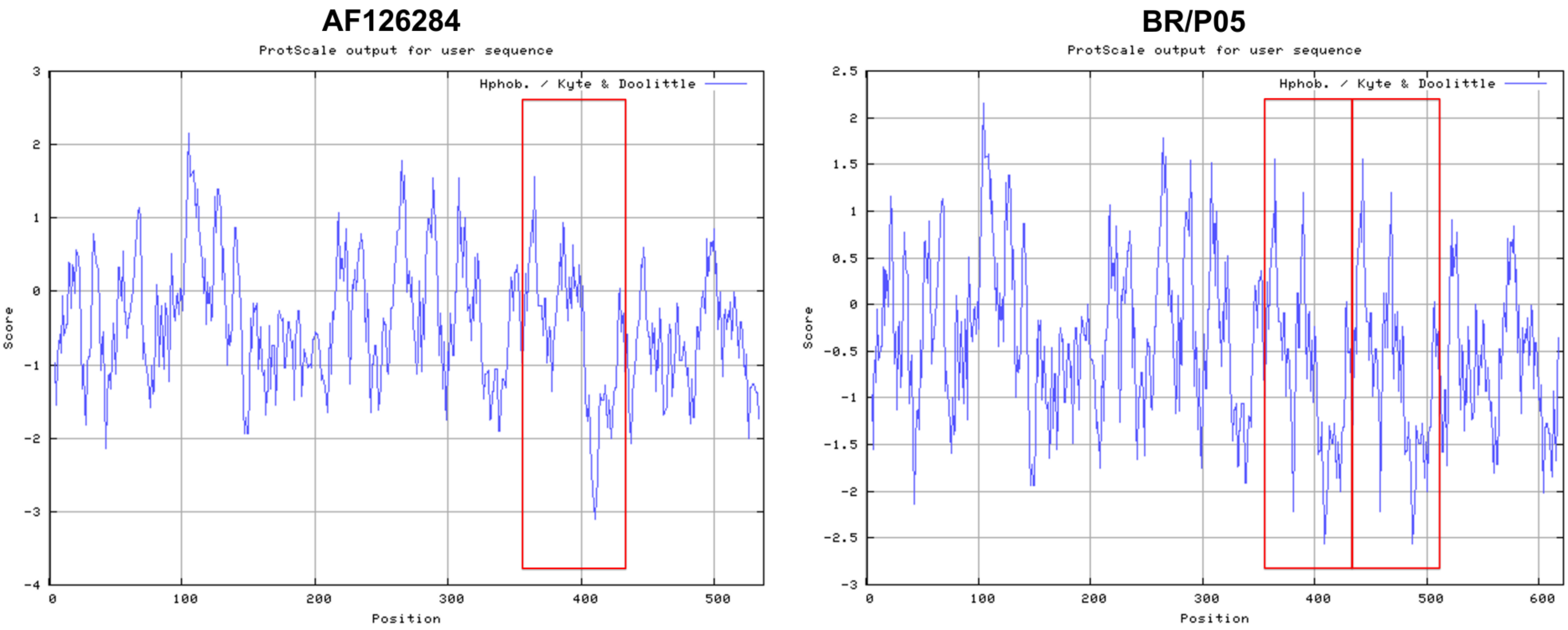

Figure S8. nsP3 hydrophobicity plot of AF126284 and BR/P05. The region corresponding to the duplication is highlighted by the red box. The Kyte & Doolittle hydrophobicity plot was drawn using the default settings, as available at the ProtScale website (http://web.expasy.org/protscale/).

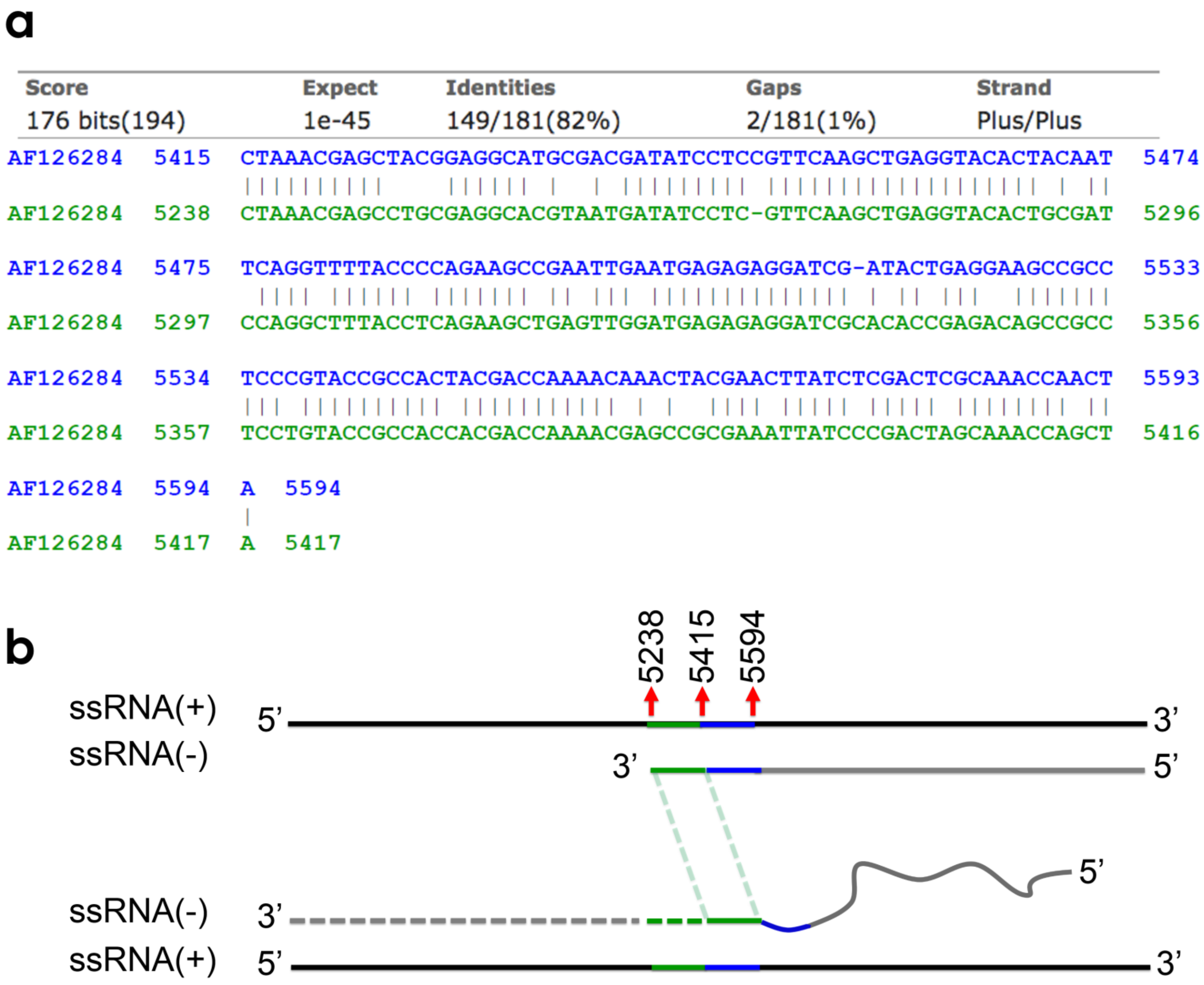

Figure S9. Mechanism of duplication hypothesis. **a** Partial results of BLAST 2 sequences search of AF126284 against itself. **b** Possible mechanism involved in the duplication. During the synthesis of the negative strand, one of these segments in the negative strand (represented in green) might hybridize with the neighboring segment in the positive strand (represented in blue), resulting in duplication of the segment represented in green (green dashed line).

Table S1

BLAST analyses of DOP-PCR sequencing results

**Clone**	**Region of homology with AF126284**	**Identity**	**E-value**
MP1	6413-6772	99%	8.10^{-176}
MP3	6413-6796<br>7826-7884	99%<br>100%	0.0<br>2.10^{-19}
MP5	8715-9366	99%	0.0
MP7	7463-7884	98%	0.0
MP9	7398-7885	98%	0.0
MP11[1]	-	-	-
MP13	7463-7884	98%	0.0
MP15	6413-6599<br>10326-10394	98%<br>94%	9.10^{-85}<br>1.10^{-19}
MP17	6413-7884	99%	0.0
MP19	7463-7884	98%	0.0
MP21	8715-9366	99%	0.0
MP23	7463-7884	98%	0.0
MP25	8715-9363	99%	0.0
MP27	7463-7885	97%	0.0
MP31	7463-7884	98%	0.0
MP33	4385-5118	98%	0.0
MP35	7409-7886	99%	0.0
MP37	7463-7884	98%	0.0

[1]Blast hits presented low coverage (<17%) and high E-values (>0.11)

Table S2

Primers used in RT-PCR and sequencing

Primer	Sequence (5’ → 3’)	Position (AF126284)
AURAV1F	ATAGCGGACGGACTAGTACTTGTACTAC	1-28
AURAV2R	TAGGGCCTGATGGTAAATGGTC	581-560
AURAV3F	GGTCAAAGGGGAGCGTGTATCGTTC	1040-1064
AURAV4R	ATAGTTCTGCATCGTGTTGGTGTTTCT	1226-1200
AURAV5F	TAGATGCCAGAAAATGCGTAAAAAGAG	2101-2127
AURAV6R	ATAGTCATTCCCCGCATTTTCTGG	2380-2357
AURAV7F	AATGACCAACCTTACTCCGTGATGT	3225-3249
AURAV8R	GTATTTGGTCCCCTTTTCCTGCTCTTA	3474-3448
AURAV9F	GTTGGAGGAAGCGACGAAGCTA	4343-4364
AURAV10R	ATCTGCTATGCGTTGCTCCCACTT	4574-4551
AURAV11F	AACGAGCTACGGAGGCATGCGACGATA	5418-5444
AURAV12R	CCAAACGTAGGGGTCGGTGATG	5674-5653
AURAV13F	GCAGCACTATTCGCAAAGACACATCGA	6537-6563
AURAV14R	GGTTCTGCTGCTTGAATAACTTGT	6691-6668
AURAV15F	CACTACGCGGGAGCCCAAGACACCT	7534-7558
AURAV16R	TAGTGAGCTGTTGGATCTGGGTAG	7760-7737
AURAV17F	GTTCTGCTGGACGCTGCTCTGA	8564-8585
AURAV18R	CGTCATCGGCATCATCCCACAC	8735-8714
AURAV19F	CACCTCTATCCCTTCTACACCGTTACA	9695-9721
AURAV20R	CATTGTTTGACTGTGTTGCCATAGGTA	9934-9908
AURAV21F	CCGGATCAGATCTATTAGCAAACACAGC	10671-10698
AURAV22R	GGCTCGGATACGCGTGTAAATGCAG	10923-10899
AURAV23F	GCTGCTATAGCCGCAATTGTGCTGG	11303-11327
AURAV24R	GAAATATTAAAAACAAAATTTTGAAATAAT	11824-11795
Nsp3F	GATCGTGAGCGCCAATTCAC	4029-4048
Nsp3R	TTTTTGTTGCAGATGCCCCG	5789-5770
DOP	CCGACTCGAGINNNNNNTGTGG	not applicable

Table S3

Dataset used in the phylogenetic analysis

GenBank Accession number	Abbreviation	Name
AJ316244	SPDV	Salmon pancreas disease virus complete genome, isolate F93125, genomic RNA
AJ316246/NC003433	SDV	Sleeping disease virus complete genome
HM147990	SESV	Southern elephant seal virus, complete genome
U73745	BFV	Barmah Forest virus strain BH2193, complete genome
EF536323	MIDV	Middelburg virus strain MIDV857 non-structural polyprotein and structural polyprotein genes, complete cds
HM147989	NDUV	Ndumu virus, complete genome
AB032553	SAGV	Sagiyama virus genomic RNA, complete genome
AY702913	GETV	Getah virus from South Korea, complete genome
GQ433354	RRV	Ross River virus strain QML 1, complete genome
HM147985	BEBV	Bebaru virus, complete genome
NC_003417	MAYV	Mayaro virus, complete genome
HM147992	UNAV	Una virus non structural polyprotein gene, partial cds; and structural polyprotein gene, complete cds
HM045788	CHIKV	Chikungunya virus strain PO731460, complete genome
AF079456	ONNV	O'nyong-nyong virus strain SG650, complete genome
L01442	VEEV IAB	Venezuelan equine encephalitis virus, complete genome
AF375051	VEEV IC	Venezuelan equine encephalitis virus nonstructural polyprotein and structural polyprotein genes, complete cds
L00930	VEEV ID	Venezuelan equine encephalitis virus strain 3880, complete genome
U34999	VEEV IE	Venezuelan equine encephalitis virus nonstructural and structural polyprotein genes, complete cds
AF075257	VEEV IF	Venezuelan equine encephalitis virus strain 78V-3531, complete genome
AF075251	EVEV	Venezuelan equine encephalitis virus strain Everglades Fe3-7c, complete genome
AF075254	TONV	Venezuelan equine encephalitis virus strain Tonate CaAn 410d, complete genome
AF075253	MUCV	Venezuelan equine encephalitis virus strain Mucambo BeAn 8, complete genome
AF075255	VEE 71D1252	Venezuelan equine encephalitis virus strain 71D-1252, complete genome
AF075256	PIXV	Venezuelan equine encephalitis virus strain Pixuna BeAr 35645, complete genome
AF075259	CABV	Venezuelan equine encephalitis virus strain Cabassou CaAr 508, complete genome
AF075258	RNV	Venezuelan equine encephalitis virus strain AG80-663, complete genome
EF151502	EEEV LinI	Eastern equine encephalitis virus strain FL93-939, complete genome
DQ241303	EEEV LinII	Eastern equine encephalitis virus strain PE-3.0815, complete genome
DQ241304	EEEV_LinIII	Eastern equine encephalitis virus strain PE-0.0155, complete genome
EF151503	EEEV_LinIV	Eastern equine encephalitis virus strain BeAr436087, complete genome

HM147991	TROV	Trocara virus non structural polyprotein gene, partial cds; and structural polyprotein gene, complete CDs
NC_018615	EILV	Eilat virus isolate EO329, complete genome
AF126284	AURAV	Aura virus polyprotein 1 and polyprotein 2 genes, complete CDs
NC001547	SINV	Sindbis virus, complete genome
M69205	SINV Ockelbo	Ockelbo virus strain Edsbyn, complete genome
HM147984	SINV Babanki	Babanki virus, complete genome
AF103728	SINV XJ160	Sindbis virus strain XJ-160, complete genome
AF429428	SINV SW6562	Sindbis virus isolate SW6562, complete genome
HM147993	WHATV	Whataroa virus, complete genome

Supplementary sequence data

In red: pGEM-T-easy sequence

>MP1
GCGTGGGCGGAATCGCATGCTCCGGGCCGCCTGGCGGCCGCGGGAATTCG
ATTCCGACTCGAGGGACTGGTGTGGAGTGCTTTAAAAAGTATGCATGTAA
TACAGACTACTGGGAAGAATTTAAAGAGAAACCAATAAGAATAACAACAG
AGTGTGTCACCTCATACGTGGCCCGATTAAAAGGACCGAAGGCTGCAGCA
CTATTCGCAAAGACACATCGACTGGTCCCGCTACAGGAGGTCCCTATGGA
TAGGTTTGTAATGGACATGAAAAGAGATGTAAAGGTGACACCCGGCACTA
AACATACCGAAGAGCGTCCCAAGGTACAAGTTATTCAAGCAGCAGAACCA
CTGGCCACGGCTTACTTGTGCGGTATACACCGGGAGCTAGTACGGAGGCT
TACAGCAGTACTGTTACCTAACATCCACACGCCATCCTCGAGTCGGAATC
ACTAGTGAATTCGCGGCCGCCTGCAGGTCGACCATATGGGAGAGCTCCCA
ACGCGTTGGATGCATAGCTTGAGTATTCTATAGTGTCACCTAAATAGCTT
GGCGTAATCATGGTCATAGCTGTTTCCTGTGTGAAATTGTTATCCGCTCA
CAATTCCACACAACATACGAGCCGGAAGCATAAAGTGTAAAGCCTGGGGT
GCCTAATGAGTGAGCTAACTCACATTAATTGCGTTGCGCTCACTGCCCGC
TTTCCAGTCGGGAAACCTGTCGTGCCAGCTGCATTAATGAATCGGCCAAC
GCGCGGGGAGAGGCGGTTTGCGTATTGGGCGCTCTTCCGCTTCCTCGCTC
ACTGACTCGCTGCGCTCGGTCGTTCGGCTGCGGCGAGCGGTATCAGCTCA
CTCAAAGGCGGTAATACGGTTATCCACAGAATCAGGGGATAACGCAGGAA
AGAACATGTGAGCAAAAGGCCAGCAAAAGGCCAGGAACCGTAAAAAGGCC
GCGTTGCTGGCGTTTTTCCATAGGCTCCGCCCCCCTGACGAGCATCACAA
AAATCGACGCTCAAGTCAGAGGTGGCGAAACCCGACAGGACTATAAAGAT
ACCAGGCGTTTCCCCCTGGAAGCTCCCTCGTGCGCTCTCCTGTTCCGACC
CTGCCGCTTACCGGATACCTGTCCGCCTTTCTCCCTTCGGGAAGCGTGGC
GCTTTCTCATAGCTCACGCTGTAGGGATCTCAGTTCCGGTGGAGGGCGTT
CCCTCCAAGCTGGGCTGGGGGGCCGAACCCCCCGTTCAGCCCGACCGGTG
GGCCTTATCCGGGAACTATCGGCTTGAGTCCAACCCGGGAAGAACAGATT
TATCGCCCTGGGGGCACCCACTGTTAAAAGGTTTTCCAAACCCAGGGAGG
TAGGCGGGGTTCACCAGTTTTTGAAGGGGGGCGTATAACGTCCCCCCAAA
AAAACAATTTGTTTTTGCTCCTCTGAACCATACCCTCCCAAAAAAAAGGA
GGTCTTTTCGGGAAAAAACCACCCCCCCGGGGGTGTTTTTTTTTTTTAAA
CAACAAAAAAGAAAAAAAAAAAAAAATAAAAATTTAATGTTGTCCCTT

>MP3
GGATGGGCCGAATCCATGCTCCGGCCGCCTGGCGGCCGCGGGAATTCGAT
TCCGACTCGAGGGGGGGTTGTGGAGTGCTTTAAAAAGTATGCATGTAATA
CAGACTACTGGGAAGAATTTAAAGAGAAACCAATAAGAATAACAACAGAG
TGTGTCACCTCATACGTGGCCCGATTAAAAGGACCGAAGGCTGCAGCACT
ATTCGCAAAGACACATCGACTGGTCCCGCTACAGGAGGTCCCTATGGATA
GGTTTGTAATGGACATGAAAAGAGATGTAAAGGTGACACCCGGCACTAAA
CATACCGAAGAGCGTCCCAAGGTACAAGTTATTCAAGCAGCAGAACCACT
GGCCACGGCTTACTTGTGCGGTATACACCGGGAGCTAGTACGGAGGCTTA
CAGCAGTACTGTTACCTAACATCCATACACTATTCGACATGTCTGCCGAG
GAAGCCGAAGACTCAAAAACCTAAGCCGAAGAAGCAAAACCAGAAACCAC
CACACCTCTGCCTCGAGTCGGAATCACTAGTGAATTCGCGGCCGCCTGCA
GGTCGACCATATGGGAGAGCTCCCAACGCGTTGGATGCATAGCTTGAGTA
TTCTATAGTGTCACCTAAATAGCTTGGCGTAATCATGGTCATAGCTGTTT
CCTGTGTGAAATTGTTATCCGCTCACAATTCCACACAACATACGAGCCGG
AAGCATAAAGTGTAAAGCCTGGGGTGCCTAATGAGTGAGCTAACTCACAT
TAATTGCGTTGCGCTCACTGCCCGCTTTCCAGTCGGGAAACCTGTCGTGC
CAGCTGCATTAATGAATCGGCCAACGCGCGGGGAGAGGCGGTTTGCGTAT
TGGGCGCTCTTCCGCTTCCTCGCTCACTGACTCGCTGCGCTCGGTCGTTC
GGCTGCGGCGAGCGGTATCAGCTCACTCAAAGGCGGTAATACGGTTATCC
ACAGAATCAGGGGATAACGCAGGAAAGAACATGTGAGCAAAAGGCCAGCA
AAAGGCCAGGAACCGTAAAAAGGCCGCGTTGCTGGCGTTTTTCCATAGGC
TCCGCCCCCCTGACGAGCATCACAAAAATCGACGCTCAAGTCAGAGGTGG

CGAAACCCGACAGGACTATAAAGATACCAGGCGTTTCCCCCTGGAAGCTC
CCTCGTGCGCTCTCCTGTTCCGACCCTGCCGCTTACCGGAAACCTGTCCG
CCTTTCTCCCTTCGGGAAGCGGGGGGCTTTTCCTAAGTCACGCTGGAGGG
TTTCCAGTTCCGGGGAAGGCGTTCGCTCCAACTGGGGCTGTTGGCCAAAC
CCCCCGTTAACCCGAACGGTGGCCCTTACCCGGAAAAATTTTTTTGGGCA
ACCCGGGAAAAACCAATATTCCCCTTGGGGGCCCCCCTGGAAAGGGTTAA
AAAAAGAGGGATGTGGGGGGGCCCCAATTTTTTTATGGGGGGGCCCCCCC
CCCCCCCAAAAA

>MP5
GGGTGGGCCACGTCCATGCTCCCGGCCGCCTGGCGGCCGCGGGAATTCGA
TTCCGACTCGAGGTAGCGCTGTGGGATGATGCCGATGACGGAGTTCTCCG
TATACAAGTAAGTGCCCAGTTAGGGTACAACAGGGCGGGCACTGCAGCTA
GCGCCCGACTCCGGTTCATGGGCGGAGGAGTGCCTCCGGAAATCCAGGAG
GGAGCAATTGCAGATTTTAAGGTCTTCACGTCTAAACCATGTTTACACCT
ATCACATAAAGGATACTTTGTCATTGTCAAGTGCCCTCCTGGTGATAGTA
TTACAACATCATTGAAAGTACATGGCTCGGATCAAACCTGCACAATTCCA
ATGCGAGTAGGTTACAAGTTCGTAGGCAAGGAAAAATATACTCTGCCACC
AATGCATGGGACACAAATACCTTGCCTTACCTACGAAAGGACACCAGAGA
AAAGTGCAGGATACGTGACCATGCATCGTCCCGGACAACAATCCATAACC
ATGCTGATGGAAGAGAGCGGAGGGGAGGTGTACGTACAACCGACCAGTGG
GCGAAACGTCACCTACGAGTGTAAATGCGGAGACTTTAAAACTGGGACTG
TCACTGCGCGCACTAAGATAGACGGCTGTACAGAAAGGAAACAATGCATT
GCGTATTTCGCCGACCACGTCAAATGGGTGTTTAACTCCCCTGACTTGAT
CAGGCATACCGACCACACAGCATCCTCGAGTCGGAATCACTAGTGAATTC
GCGGCCGCCTGCAGGTCGACCATATGGGAGAGCTCCCAACGCGTTGGATG
CATAGCTTGAGTATTCTATAGTGTCACCTAAATAGCTTGGCGTAATCATG
GTCATAGCTGTTTCCTGTGTGAAATTGTTATCCGCTCACAATTCCACACA
ACATACGAGCCGGAAGCATAAAGTGTAAAGCCTGGGGTGCCTAATGAGTG
AGCTAACTCACATTAATTGCGTTGCGCTCACTGCCCGCTTTCCAGTCGGG
AAACCTGTCGTGCCAGCTGCATTAATGAATCGGCCAACGCGCGGGGAGAG
GCGGTTTGCGTATTGGGCGCTCTTCCGCTTCCTCGCTCACTGACTCGCTG
CGCTCGGTCGTTCGGCTGCGGCGAGCGGTATCAGCTCACTCAAAGGCGGG
AATACGGTTATCCACAGAATCAGGGGATAACGCAGGAAAGAACATGGGAG
CAAAAGGCCCGCAAAAGGCCCGGAACCGTAAAAAGGCCCGTTGCTGGCGT
TTTTCTAAAGGTCCCGCCCCCTGACAAACTTCCAAAAATCCACCCTCAAT
CAAAAGTGGCGAAACCCCCAGGACTTAAAAATCCACGGGTTCCTCCGGAA
GACCCCCGGGGGCTCCTTGTTAACCCCGCCTTACAGAAAATGGCCCCTTT
CCTTTTTGGAAGAGAGGGTTCTTATTCACCACGAGGGTTCAATTTTTGTG
GGGGTGTCCACACCGGTGGGGGGAACCCCCACCCACCCACCGCCCCTCCA
ATTAATATTTATTTTTAAGATATATTCTCTAACCCCCCCCCCAAAAACAA
AAAACAAAAAAAGGGGGGGGTGTTGTTGTTGTGGGTT

>MP7
GGTAGGCCGACTCGCATGCTCTCGGCCGCCATGGCGGCCGCGGGAATTCG
ATTCCGACTCGAGGGGCTTCTGTGGTACAAGAGGTTCAGCCAGTGCTGTT
AGCCCTCGCTACGTTCTCGCGTAGCGATAAAGCATTTAAAGCACTACGCG
GGAGCCCAAGACACCTCTACGGTGGTCCTAAATAGACGGTGTAGCACAGT
AGATCGTCTAAATTATTTCACATCTTTACGTTACTATGAACTCTGTCTTT
TACAATCCGTTTGGTCGAGGTGCCTACGCTCAACCTCCAATAGCATGGAG
GCCAAGACGTAGGCGTGCACCTGCGCCTCGACCATCCGGGTTGACTACCC
AGATCCAACAGCTCACTAGGGCTGTTGGAGCTTTGGTGCTGGACAATGCT
ACACGTCGCCAGCGCCCGGCTCCTCGCACGGCGCCGAGGAAGCCGAAGAC
TCAAAAACCTAAGCCGAAGAAGCAAAACCAGAAACCACCACACCTAACCC
TCGAGTCGGAATCACTAGTGAATTCGCGGCCGCCTGCAGGTCGACCATAT
GGGAGAGCTCCCAACGCGTTGGATGCATAGCTTGAGTATTCTATAGTGTC
ACCTAAATAGCTTGGCGTAATCATGGTCATAGCTGTTTCCTGTGTGAAAT
TGTTATCCGCTCACAATTCCACACAACATACGAGCCGGAAGCATAAAGTG
TAAAGCCTGGGGTGCCTAATGAGTGAGCTAACTCACATTAATTGCGTTGC
GCTCACTGCCCGCTTTCCAGTCGGGAAACCTGTCGTGCCAGCTGCATTAA

TGAATCGGCCAACGCGCGGGGAGAGGCGGTTTGCGTATTGGGCGCTCTTC
CGCTTCCTCGCTCACTGACTCGCTGCGCTCGGTCGTTCGGCTGCGGCGAG
CGGTATCAGCTCACTCAAAGGCGGTAATACGGTTATCCACAGAATCAGGG
GATAACGCAGGAAAGAACATGTGAGCAAAAGGCCAGCAAAAGGCCAGGAA
CCGTAAAAAGGCCGCGTTGCTGGCGTTTTCCATAGGCTCCGCCCCCCTGA
CGAGCATCACAAAAATCGACGCTCAAGTCAGAGGTGGCGAAACCCGACAG
GACTATAAAGATACCAGGCGTTTCCCCCTGGAAGCTCCCTCGTGCGCTCT
CCTGTTCCGACCCTGCCGCTTACCGGATACCTGTCCGCCTTTCTCCCTTC
GGGAAACGTGGGGCTTTTCCAAAGTCCACCTGGAGGGTTTCCAATTCCGG
GGAAGGCGTTCCCTCCAACTTGGTTGTGTGGCAAAACCCCCGTTTAGCCC
AACGGTTGGCCTTATTCGGGAATATATTCTTGGGTCAACCCCGGGAAAAC
ACATTTTTTCCCTTGGGGAGCCCCTGGTACAGGATTAAGAAACAGGAATT
ATGGGGTGTCCCAAATTTTTAATGGGGGCTACCCGTCCCTCCAAAACAAA
TTTATTTTTTCCCTCTCCCCCGCTCCCTCAAAAAAAGATTATTTTTTTAC
ACCACCCCACACCGGGGGTTTTTTGTGGTTTTTCAA

>MP9
GTGGTGCGAGGCGCATGCTCCCGGCCGCCTGGCGGCCGCGGGAATTCGAT
TCCGACTCGAGGCAGTGGTGTGGTTCAGAGTGGGGATCCAAGGAGAACTT
TTGAAGGCCGTCGAGTCACGCTATGAGGTACAAGAGGTTCAGCCAGTGCT
GTTAGCCCTCGCTACGTTCTCGCGTAGCGATAAAGCATTTAAAGCACTAC
GCGGGAGCCCAAGACACCTCTACGGTGGTCCTAAATAGACGGTGTAGCAC
AGTAGATCGTCTAAATTATTTCACATCTTTACGTTACTATGAACTCTGTC
TTTTACAATCCGTTTGGTCGAGGTGCCTACGCTCAACCTCCAATAGCATG
GAGGCCAAGACGTAGGCGCGCACCTGCGCCTCGACCATCCGGGTTGACTA
CCCAGATCCAACAGCTCACTAGGGCTGTTGGAGCTTTGGTGCTGGACAAT
GCTACACGTCGCCAGCGCCCGGCTCCTCGCACGGCGCCGAGGAAGCCGAA
GACTCAAAAACCTAAGCCGAAGAAGCAAAACCAGAAACCACCACAAACCC
CCCTCGAGTCGGAATCACTAGTGAATTCGCGGCCGCCTGCAGGTCGACCA
TATGGGAGAGCTCCCAACGCGTTGGATGCATAGCTTGAGTATTCTATAGT
GTCACCTAAATAGCTTGGCGTAATCATGGTCATAGCTGTTTCCTGTGTGA
AATTGTTATCCGCTCACAATTCCACACAACATACGAGCCGGAAGCATAAA
GTGTAAAGCCTGGGGTGCCTAATGAGTGAGCTAACTCACATTAATTGCGT
TGCGCTCACTGCCCGCTTTCCAGTCGGGAAACCTGTCGTGCCAGCTGCAT
TAATGAATCGGCCAACGCGCGGGGAGAGGCGGTTTGCGTATTGGGCGCTC
TTCCGCTTCCTCGCTCACTGACTCGCTGCGCTCGGTCGTTCGGCTGCGGC
GAGCGGTATCAGCTCACTCAAAGGCGGTAATACGGTTATCCACAGAATCA
GGGGATAACGCAGGAAAGAACATGTGAGCAAAAGGCCAGCAAAAGGCCAG
GAACCGTAAAAAGGCCGCGTTGCTGGCGTTTTTCCATAGGCTCCGCCCCC
CTGACGAGCATCACAAAAATCGACGCTCAAGTCAGAGGTGGCGAAACCCG
ACAGGACTATAAAGAAACCAAGGCGTTTCCCCCTGGAAAGTCCCCCCGGG
GGCTTCCCTGTTCCAACCCTGCCGGTTACCGGAAAACTGGTCGCCTTTCT
CCCTTCGGGAGAGGGGGGCGTTTTCTAAACTCACCCCTGAGGGATTCAAA
TTCCGGGGGAGGGGGTTCCTTCCAACTTGGGGGTGGGGTGAAAACCCCCC
GTCATCCCGAACCGTGGCCTTTACTTGAGAAAATCTCTTTGTGTTACCCC
GGGAGAAAAAAATATCCCCCTTGCCAGGCCCCGGGCAAGGGTCAGAGAAA
AAGGGATTTAGGGGGGTGGGAAATTTTTTATGTGGGGGTACCCGCTCCTT
CAACACATTATAATAAACCTCCCTGCACCTCCTTCTCAAAAAAAGGTTAT
TGTGGGTTTACAAACCAAAAGGGAAAAGGGGGGGTTTGTTCTGGGGGCG
AAAAAAGAAAAAGGAAAGGCAAAAAAGGGGCGGAAAAAAAAAAACCCCCC
AAAAAAA

>MP11
GGATGGGCGGAGTCGCATGCTCCCGGCCGCCATGGCGGCCGCGGGAATTC
GATTCCGACTCGAGGTGCGCCTGTGGAAGAAATTGAGTGAGCAAGGGAAG
AAGCAAATTGCTCCTAAAAATGCTAATAATATGGAAGTGAAGTAGAAACC
AAAAGGGGAAGCTGCAAAAGTCCGGTTTTCGTTCGATTCTTTGCTGTTCC
GCAGTAGGCTGCTGAGGGCGGCGGTGCTATCGATCATAATAAATCGCAGC
AAGCCACCGCAGAGCGTATCATCAAGGTGCTACCTATGCCAGAGCCTACT
ACTGTACCAGCGAACCCAAAAGGATCCCTTCCAGGAGGAATAAAAAGATA

ACCACCCACACGTTTACCTCGAGTCGGAATCACTAGTGAATTCGCGGCCG
CCTGCAGGTCGACCATATGGGAGAGCTCCCAACGCGTTGGATGCATAGCT
TGAGTATTCTATAGTGTCACCTAAATAGCTTGGCGTAATCATGGTCATAG
CTGTTTCCTGTGTGAAATTGTTATCCGCTCACAATTCCACACAACATACG
AGCCGGAAGCATAAAGTGTAAAGCCTGGGGTGCCTAATGAGTGAGCTAAC
TCACATTAATTGCGTTGCGCTCACTGCCCGCTTTCCAGTCGGGAAACCTG
TCGTGCCAGCTGCATTAATGAATCGGCCAACGCGCGGGGAGAGGCGGTTT
GCGTATTGGGCGCTCTTCCGCTTCCTCGCTCACTGACTCGCTGCGCTCGG
TCGTTCGGCTGCGGCGAGCGGTATCAGCTCACTCAAAGGCGGTAATACGG
TTATCCACAGAATCAGGGGATAACGCAGGAAAGAACATGTGAGCAAAAGG
CCAGCAAAAGGCCAGGAACCGTAAAAAGGCCGCGTTGCTGGCGTTTTTCC
ATAGGCTCCGCCCCCCTGACGAGCATCACAAAAATCGACGCTCAAGTCAG
AGGTGGCGAAACCCGACAGGACTATAAAGATACCAGGCGTTTCCCCCTGG
AAGCTCCCTCGTGCGCTCTCCTGTTCCGACCCTGCCGCTTACCGGATACC
TGTCCGCCTTTCTCCCTTCGGGAAGCGTGGCGCTTTCTCATAGCTCACGC
TGTAGGTATCTCAGTTCGGGGGAGGTCGTTCGCTCCAAGCTGGGCTGTGT
GGACAAACCCCCCGTTTAGCCCGACCGGTGGGCCTTATCCGGGAACATTC
GCCTTGAGTCCAACCCGGTAAGACACGACTTATTCGCCCTGGGGACAGCC
CTTGGTAAAAGGTTAACAAAACCAGGGATGTAGGCGGGGGTCCAAAATTC
TTTAAAGGGGGGCGTACTACCGTACCTTAAAAAAAAAATTTTGTTATTGC
GCCTCTCTGACCCGTACCCTCCGAAAAAAATGGAGGCCTGTTCCGCAAAA
CACCAACCAACCCCGGCGGGTTGTGTTTGTGTGGGTTAACCCCCCAACAA
AAGACAAAAGAACAAATTTATTTTTCTTTGGGGGTCCTCTGGAGACACCC
CCCCGGGGGGGCCGGGCGGGAAGAAGAGCGACCAGGGGATAAAAAAATTA
ATAAAATATATAATATTAAATAAAAAAAAAAATAAAAAAAAAAAGACCAGA
AAAAAAAAAAAAAAAAAAAAAAAAACCCACCGCC

>MP13
GGGTGGCCGAGGTCCCATGCTCCCGGCCGCCATGGCGGCCGCGGGAATTC
GATTCCGACTCGAGGGGCTTCTGTGGTACAAGAGGTTCAGCCAGTGCTGT
TAGCCCTCGCTACGTTCTCGCGTAGCGATAAAGCATTTAAAGCACTACGC
GGGAGCCCAAGACACCTCTACGGTGGTCCTAAATAGACGGTGTAGCACAG
TAGATCGTCTAAATTATTTCACATCTTTACGTTACTATGAACTCTGTCTT
TTACAATCCGTTTGGTCGAGGTGCCTACGCTCAACCTCCAATAGCATGGA
GGCCAAGACGTAGGCGTGCACCTGCGCCTCGACCATCCGGGTTGACTACC
CAGATCCAACAGCTCACTAGGGCTGTTGGAGCTTTGGTGCTGGACAATGC
TACACGTCGCCAGCGCCCGGCTCCTCGCACGGCGCCGAGGAAGCCGAAGA
CTCAAAAACCTAAGCCGAAGAAGCAAAACCAGAAACCACCACACCTAACC
CTCGAGTCGGAATCACTAGTGAATTCGCGGCCGCCTGCAGGTCGACCATA
TGGGAGAGCTCCCAACGCGTTGGATGCATAGCTTGAGTATTCTATAGTGT
CACCTAAATAGCTTGGCGTAATCATGGTCATAGCTGTTTCCTGTGTGAAA
TTGTTATCCGCTCACAATTCCACACAACATACGAGCCGGAAGCATAAAGT
GTAAAGCCTGGGGTGCCTAATGAGTGAGCTAACTCACATTAATTGCGTTG
CGCTCACTGCCCGCTTTCCAGTCGGGAAACCTGTCGTGCCAGCTGCATTA
ATGAATCGGCCAACGCGCGGGGAGAGGCGGTTTGCGTATTGGGCGCTCTT
CCGCTTCCTCGCTCACTGACTCGCTGCGCTCGGTCGTTCGGCTGCGGCGA
GCGGTATCAGCTCACTCAAAGGCGGTAATACGGTTATCCACAGAATCAGG
GGATAACGCAGGAAAGAACATGTGAGCAAAAGGCCAGCAAAAGGCCAGGA
ACCGTAAAAAGGCCGCGTTGCTGGGGGTTTTTCCATAGGCTCCGCCCCCC
TGACGAGCATCACAAAAATCGACGCTCAAGTCAGAGGTGGCGAAACCCGA
CAGGACTATAAAGATACCAAGGCGTTTCCCCCTGGGAAGCTTCCCTCGGG
CGCTCTCCCTGTTCCGACCCTGGCCGCTTACCGGGAAACCTGGTCCGCCT
TTTTCCCCTTCGGGGAAACGGGGGGCCTTTTCCCA

>MP15
GGATAGCGACGTCGCATGCTCCCGGCCGCCTGGCGGCCGCGGGAATTCGA
TTCCGACTCGAGGTTAGACTGTGGCGCAATCTGTTGACAGTTCGACGTAT
GCTTCGCTAAGCTGACTGTTTTCGGAGTCGCAAAAACCTATCCATAGGGA
CCTCCTGTAGCGGGACCAGTCGATGTGTCTTTGCGAATAGTGCTGCAGCC
TTCGGTCCTTTTAATCGGGCCACGTATGAGGTGACACACTCTGTTGTTAT

TCTTATTGGTTTCTCTTTAAATTCTTCCCAGTAGTCTGTATTACATGCAT
ACTTTTTAAAGCACTCCACACGCTTACCTCGAGTCGGAATCACTAGTGAA
TTCGCGGCCGCCTGCAGGTCGACCATATGGGAGAGCTCCCAACGCGTTGG
ATGCATAGCTTGAGTATTCTATAGTGTCACCTAAATAGCTTGGCGTAATC
ATGGTCATAGCTGTTTCCTGTGTGAAATTGTTATCCGCTCACAATTCCAC
ACAACATACGAGCCGGAAGCATAAAGTGTAAAGCCTGGGGTGCCTAATGA
GTGAGCTAACTCACATTAATTGCGTTGCGCTCACTGCCCGCTTTCCAGTC
GGGAAACCTGTCGTGCCAGCTGCATTAATGAATCGGCCAACGCGCGGGGA
GAGGCGGTTTGCGTATTGGGCGCTCTTCCGCTTCCTCGCTCACTGACTCG
CTGCGCTCGGTCGTTCGGCTGCGGCGAGCGGTATCAGCTCACTCAAAGGC
GGTAATACGGTTATCCACAGAATCAGGGGATAACGCAGGAAAGAACATGT
GAGCAAAAGGCCAGCAAAAGGCCAGGAACCGTAAAAAGGCCGCGTTGCTG
GCGTTTTTCCATAGGCTCCGCCCCCCTGACGAGCATCACAAAAATCGACG
CTCAAGTCAGAGGTGGCGAAACCCGACAGGACTATAAAGATACCAGGCGT
TTCCCCCTGGAAGCTCCCTCGTGCGCTCTCCTGTTCCGACCCTGCCGCTT
ACCGGATACCTGTCCGCCTTTCTCCCTTCGGGAAGCGTGGCGCTTTCTCA
TAGCTCACGCTGTAGGTATCTCAGTTCGGTGTAGGTCGTTCGCTCCAAGC
TGGGCTGTGTGCACGAACCCCCCGTTCAGCCCGACCGCTGCGCCTTATCC
GGGAACTATCGTCTTGAGTCCAACCCGGTAAGACACGACTTATCCCCACT
GGGGGCAGCCACTGGGAACAGGATTACCAAAACAAGGTATTAAACGGGGC
GTCCAAAGTTCTTTAAAGGGGGGCCTACACCCCGCTCCCTTAAAAAACCA
AATTTTGGACCTCGGCTTTGTTGAACCCGTTACCTCCCGGAAAAAGATAG
GGGGGTCTTTCCCCGCGAAAACAACCCACGGACACGGGGGTTTTTGTGT
TGACACCGAAAAAACCCAACAACAAGAAAAAAAAAAAAATTAATTTTTCTC
TGGGTGGGCGGCGGGCGAACAAAACAAAAGAAATAGGTGGTTTTTAAGAG
ATTTAACCTTTGTTTTCCCCAAAATAATAAAAATTAAATAAAAAAATTAA
AAAATAAAAATGAAAAATGAAAAAAGAAGAAAAAGACCCAAACCCCCCCC
CCCCGCGGCCCCCCCCCCCCCCCCCCCCCCCTCCTTTCCCCCCCCCCC
CTCCCGGCCCCCGCCCCCCAAAGGTCCAAGGGAAAAAAAAAAAAAGAAGA
AACCACATTTAAGGAAATCTAGGGGAACAACGGGGGGAAAAAAAAAAATG
TTGGACGCCTAACACGGGAATTTCACGAAAAAATGTGGAAGTTCGGCGTA
AAAAAACCGGCCCCCCCC

>MP17_consensus (Assembled with: http://www.hpa-bioinformatics.org.uk/cgi-bin/assembly_tool/seq_assemble.cgi?no=2)
atggtcgacctgcaggcggccgcgaattcactagtgattccgactcgaggtgaaggtgtggtggtttctggttttgcttcTTCGGCTTAGGTTT
TTGAGTCTTCGGCTTCCTCGGCGCCGTGCGAGGAGCCGGGCGCTGGCGACGTGTAGCATTGT
CCAGCACCAAAGCTCCAACAGCCCTAGTGAGCTGTTGGATCTGGGTAGTCAACCCGGATGGC
CGAGGCGCAGGTGCACGCCTACGTCTTGGCCTCCATGCTATTGGAGGTTGAGCGTAGGCACC
TCGACCAAACGGATTGTAAAAGACAGAGTTCATAGTAACGTAAAGATGTGAAATAATTTAG
ACGATCTACTGTGCTACACCGTCTATTTAGGACCACCGTAGAGGTGTCTTGGGCTCCCGCGT
AGTGCTTTAAATGCTTTATCGCTACGCGAGAACGTAGCGAGGGCTAACAGCACTGGCTGAAC
CTCTTGTACCTCATAGCGTGACTCGACGGCCTTCAAAAGTTCTCCTTGGATCCCCACTCTGAA
CCACGCCCTTGTCTCATCTTCCAGCGCTCTACGCCTGTCGTGATCCTGCTCATCATCTACTGG
TAGAGGCTTGCCTAGTTTGAACAGTCTCTTAAGCGGATCAGAGACCCGGCACGCTGTGTGCG
TCACTTGGTCTTCTAGAATAAATCCGCCGCAAAAGTAAGGGGCCTTGATACCAATGACCGTG
TCTATGATTTTCACTTCCATATTCATCCAAGTGGCGCATCTGTCGGCCATTATCTTGTCCGAA
ACGACACCATGTATAATGTTGTCGTCCCCTATGAAAGCTGCGCATTTGGAGTCCCTCAACCG
TTGTTCTAGAACTCGGCTAGCAATAACTACATTCAACACAGTGTTAACAAACAGTGTTAAAA
ACATTCCGGACTTCATCATAGCACCAAATTTAAACCTGGTTGCTGTCGGTAGGTGGACGCTT
GTGATCTCCCCGAAGGCACATTCAATGAGGTCCATCAGGCGGTCATCCACTCCTAAGTCTTC
CAGAATCATCAGTGCAGTCAGGGCTAATGAATCATCCTGGCTCTTGTCGAAAGACGCTATAT
CTGTTTCTAGCACCGGGTCACCGTAAGAAAAATTTGCTGCTATGATCGCGTCGAAATCCTCG
GCAGACATGTCGAATAGTGTATGGATGTTAGGTAACAGTACTGCTGTAAGCCTCCGTACTAG
CTCCCGGTGTATACCGCACAAGTAAGCCGTGGCCAGTGGTTCTGCTGCTTGAATAACTTGTA
CCTTGGGACGCTCTTCGGTATGTTTAGTGCCGGGTGTCACCTTTACATCTCTTTTCATGTCCAT
TACAAACCTATCCATAGGGACCTCCTGTAGCGGGACCAGTCGATGTGTCTTTGCGAATAGTG
CTGCAGCCTTCGGTCCTTTTAATCGGGCCACGTATGAGGTGACACACTCTGTTGTTATTCTTA

TTGGTTTCTCTTTAAATTCTTCCcagtagtctgtattacatgcatactttttaaagcactccacaccactacctcgagtcggaatcgaatt
cccgcggccgcc

>MP19
GGATGGCCGACTTCGCATGCTCCCGGCCGCCATGGCGGCCGCGGGAATTC
GATTCCGACTCGAGGGGCTTCTGTGGTACAAGAGGTTCAGCCAGTGCTGT
TAGCCCTCGCTACGTTCTCGCGTAGCGATAAAGCATTTAAAGCACTACGC
GGGAGCCCAAGACACCTCTACGGTGGTCCTAAATAGACGGTGTAGCACAG
TAGATCGTCTAAATTATTTCACATCTTTACGTTACTATGAACTCTGTCTT
TTACAATCCGTTTGGTCGAGGTGCCTACGCTCAACCTCCAATAGCATGGA
GGCCAAGACGTAGGCGTGCACCTGCGCCTCGACCATCCGGGTTGACTACC
CAGATCCAACAGCTCACTAGGGCTGTTGGAGCTTTGGTGCTGGACAATGC
TACACGTCGCCAGCGCCCGGCTCCTCGCACGGCGCCGAGGAAGCCGAAGA
CTCAAAAACCTAAGCCGAAGAAGCAAAACCAGAAACCACCACACCTAACC
CTCGAGTCGGAATCACTAGTGAATTCGCGGCCGCCTGCAGTCGACCATAT
GGGAGAGCTCCCAACGCGTTGGATGCATAGCTTGAGTATTCTATAGTGTC
ACCTAAATAGCTTGGCGTAATCATGGTCATAGCTGTTTCCTGTGTGAAAT
TGTTATCCGCTCACAATTCCACACAACATACGAGCCGGAAGCATAAAGTG
GTAAGCCTGGGGTGCCTAATGAGTGAGCTTACTCACATTAATTGCGTTGC
GCTCACTGCCCGCTTTCCAGTCGGGAAACCTGTCGTGCCAGCTGCATTAA
TGAATCGGCCCACGCGCGGGGAGAGGCGGTTGCGTATTGGGCGCTCTTC
CGCTTTCCTCGCTCCTGACTCGCTGCGCTCGGTCGTCGGCTGCGGCAAGC
GGATCAGCTCACTCAAGGCGGTATACGGTATCCCAGAATCGGGGAAACGC
AGGAAAAACAGGGAGCAAAGGCAGCAAAGGCCGG

>MP21
AAGACGGCGTACGCGCATGCTCCCGGCCGCCATGGCGGCCGCGGGAATTC
GATTCCGACTCGAGGTAGCGCTGTGGGATGATGCCGATGACGGAGTTCTC
CGTATACAAGTAAGTGCCCAGTTAGGGTACAACAGGGCGGGCACTGCAGC
TAGCGCCCGACTCCGGTTCATGGGCGGAGGAGTGCCTCCGGAAATCCAGG
AGGGAGCAATTGCAGATTTTAAGGTCTTCACGTCTAAACCATGTTTACAC
CTATCACATAAAGGATACTTTGTCATTGTCAAGTGCCCTCCTGGTGATAG
TATTACAACATCATTGAAAGTACATGGCTCGGATCAAACCTGCACAATTC
CAATGCGAGTAGGTTACAAGTTCGTAGGCAAGGAAAAATATACTCTGCCA
CCAATGCATGGGACACAAATACCTTGCCTTACCTACGAAAGGACACCAGA
GAAAAGTGCAGGATACGTGACCATGCATCGTCCCGGACAACAATCCATAA
CCATGCTGATGGAAGAGAGCGGAGGGGAGGTGTACGTACAACCGACCAGT
GGGCGAAACGTCACCTACGAGTGTAAATGCGGAGACTTTAAAACTGGGAC
TGTCACTGCGCGCACTAAGATAGACGGCTGTACAGAAAGGAAACAATGCA
TTGCGTATTTCGCCGACCACGTCAAATGGGTGTTTAACTCCCCTGACTTG
ATCAGGCATACCGACCACACAGCATCCTCGAGTCGGAATCACTAGTGAAT
TCGCGGCCGCCTGCAGGTCGACCATATGGGAGAGCTCCCAACGCGTTGGA
TGCATAGCTTGAGTATTCTATAGTGTCACCTAAATAGCTTGGCGTAATCA
TGGTCATAGCTGTTTCCTGTGTGAAATTGTTATCCGCTCACAATTCCACA
CAACATACGAGCCGGAAGCATAAAGTGTAAAGCCTGGGGTGCCTAATGAG
TGAGCTAACTCACATTAATTGCGTTGCGCTCACTGCCCGCTTTCCAGTCG
GGAAACCTGTCGTGCCAGCTGCATTAATGAATCGGCCAACGCCCGGGGAA
AGGCGGTTTGCGTATTGGGCGCTCTTCCGCTTCCTCGCTCACTGACTCGC
TGCGCTCGGTCGTTCGGCTGCGGCGAGCGGTATCAGCTCACTCAAAGGCG
GGAATACGGTTATCCCCAGAATCAGGGGATAAAG

>MP23
GGCTGGCGAGGTGCATGGCTCCGGCCGCCTGGCGGCCGCGGGAATTCGAT
TCCGACTCGAGGGGCTTCTGTGGTACAAGAGGTTCAGCCAGTGCTGTTAG
CCCTCGCTACGTTCTCGCGTAGCGATAAAGCATTTAAAGCACTACGCGGG
AGCCCAAGACACCTCTACGGTGGTCCTAAATAGACGGTGTAGCACAGTAG
ATCGTCTAAATTATTTCACATCTTTACGTTACTATGAACTCTGTCTTTTA
CAATCCGTTTGGTCGAGGTGCCTACGCTCAACCTCCAATAGCATGGAGGC
CAAGACGTAGGCGTGCACCTGCGCCTCGACCATCCGGGTTGACTACCCAG
ATCCAACAGCTCACTAGGGCTGTTGGAGCTTTGGTGCTGGACAATGCTAC
ACGTCGCCAGCGCCCGGCTCCTCGCACGGCGCCGAGGAAGCCGAAGACTC

AAAAACCTAAGCCGAAGAAGCAAAACCAGAAACCACCACACCTAACCCTC
GAGTCGGAATCACTAGTGAATTCGCGGCCGCCTGCAGGTCGACCATATGG
GAGAGCTCCCAACGCGTTGGATGCATAGCTTGAGTATTCTATAGTGTCAC
CTAAATAGCTTGGCGTAATCATGGTCATAGCTGTTTCCTGTGTGAAATTG
TTATCCGCTCACAATTCCACACAACATACGAGCCGGAAGCATAAAGTGTA
AAGCCTGGGGTGCCTAATGAGTGAGCTAACTCACATTAATTGCGTTGCGC
TCACTGCCCGCTTTCCAGTCGGGAAACCTGTCGTGCCAGCTGCATTAATG
AATCGGCCAACGCGCGGGGAGAGGCGGTTTGCGTATTGGGCGCTCTTCCG
CTTCCTCGCTCACTGACTCGCTGCGCTCGGTCGTTCGGCTGCGGCGAGCG
GTATCAGCTCACTCAAAGGCGGTAATACGGTTATCCACAGAATCAGGGGA
TAACGCAGGAAAGAACATGTGAGCAAAAGGCCAGCAAAAGGCCAGGAACC
GTAAAAAGGCCGCGTTGCTGGCGTTTTCCATAGGCTCCGCCCCCCTGACG
AGCATCACAAAAATCGACGCTCAAGTCAGAGGTGGCGAAACCCGACAGGA
CTTTAAAGATACCAGGCGTTTCCCCCTGGAAAGTCCCTCGTGGGGTCTCC
TGTTCGGACCCTGCCGCTTACCGGATACTGGCCGCCTTTTTCCCTTCGGG
AGAGGGGGGGCTTTCTCTAACTCACCCCGGAGGGATTCAAATTCGGGGGA
AGGGGGTTCGTTCCAACCTGGGGTTGTGTGCAGAACCCCCCGTCAACCCG
AACGCGGCGCCTTATCTGGAAATATAACTATGGTGCACCCGGGAGACAGA
CATATCCCCTTGGCGCGCCCCCGGGAAAGATAAAAAAAAAAAGATAAAGG
GGGGGGGAAGTTTTTTAATGGGGGTTACCCGGCCTCAAAAAAATATTTTT
TATGTCTTGGGAGGTTTCTCCCAAAAAGAGTTTGGCGAAAAACAAAAAAG
GGGGTGTGTGTTGGTTGGTAAGAAAAAAAAGAAGAGAATTTATTTTTGGG
GAAAAAAAAAAAA

>MP25
GACTGGCCAACCTCCATGCTCCCGGCCGCCTGGCGGCCGCGGGAATTCGA
TTCCGACTCGAGGATGAGTTGTGGGATGATGCCGATGACGGAGTTCTCCG
TATACAAGTAAGTGCCCAGTTAGGGTACAACAGGGCGGGCACTGCAGCTA
GCGCCCGACTCCGGTTCATGGGCGGAGGAGTGCCTCCGGAAATCCAGGAG
GGAGCAATTGCAGATTTTAAGGTCTTCACGTCTAAACCATGTTTACACCT
ATCACATAAAGGATACTTTGTCATTGTCAAGTGCCCTCCTGGTGATAGTA
TTACAACATCATTGAAAGTACATGGCTCGGATCAAACCTGCACAATTCCA
ATGCGAGTAGGTTACAAGTTCGTAGGCAAGGAAAAATATACTCTGCCACC
AATGCATGGGACACAAATACCTTGCCTTACCTACGAAAGGACACCAGAGA
AAAGTGCAGGATACGTGACCATGCATCGTCCCGGACAACAATCCATAACC
ATGCTGATGGAAGAGAGCGGAGGGGAGGTGTACGTACAACCGACCAGTGG
GCGAAACGTCACCTACGAGTGTAAATGCGGAGACTTTAAAACTGGGACTG
TCACTGCGCGCACTAAGATAGACGGCTGTACAGAAAGGAAACAATGCATT
GCGTATTTCGCCGACCACGTCAAATGGGTGTTTAACTCCCCTGACTTGAT
CAGGCATACCGACCACACAATCACTAGTGAATTCGCGGCCGCCTGCAGGT
CGACCATATGGGAGAGCTCCCAACGCGTTGGATGCATAGCTTGAGTATTC
TATAGTGTCACCTAAATAGCTTGGCGTAATCATGGTCATAGCTGTTTCCT
GTGTGAAATTGTTATCCGCTCACAATTCCACACAACATACGAGCCGGAAG
CATAAAGTGTAAAGCCTGGGGTGCCTAATGAGTGAGCTAACTCACATTAA
TTGCGTTGCGCTCACTGCCCGCTTTCCAGTCGGGAAACCTGTCGTGCCAG
CTGCATTAATGAATCGGCCAACGCGCGGGGAGAGGCGGTTTGCGTATTGG
GCGCTCTTCCGCTTCCTCGCTCACTGACTCGCTGCGCTCGGTCGTTCGGC
TGCGGCGAGCGGGATCAGCTCACTCAAAGGCGGGAATACGGGTATCCACA
GAATCAGGGGATAACCCAGGAAAGAACATGTGAGCAAAAGGCCCGCAAAA
GGCCCGGAACCGTAAAAAGGCCCGTTGCTTGGGGTTTTTCCTAAGGGCCC
GCCCCCCTGACAAGAATCCAAAAATCAACCCTCAAGCAAAAGTGGGGAAA
CCCCCCGGGATTAAAAATACAAGGGGTTCCCCTGGAAGTCCCCTGGGCGT
CCTTGTCGAACTGCGCGTACGAGAACATGCCCCTTTCCCTTTCGGGAGGG
GGGTTCCATATCCCCCCGGGGGGTACAACCTTGGGGAGGGGGGTCCCCAC
GGGGTGGTGGGAACCCCGCCCCCCACCGCCCCTCCCGCAACTTTTTTTCC
CTAGCGCTTTCTTTTCCCCCCGGGCCCCCCCGGGCCGGGGAGGAGGGGGG
GAGGGGGGGGGGGGGGGGGGGGGGGGGGGGGGGGGGGTTGATTTTTTTT
TTTTTTTTTTTTTTGAAAACCCCCCCCCCCCCCCCCCCTCTCCCGGGTT
CAGGGGTCAAGGGCCCCGCAGAGTCGCAATTTATTTTTTAATCGCTTTAA
CATTTTTTTAATTTTCACCAAAAAATGAAGAATTTTTGAATTTTAAAAAT

AAGAAAAAACAAAACCTTTAAAACCCGCTTTTCCCCGCCCAAAGGCCGG

>MP27
GGGTAGGCGAACGTCGCATGGCTCCCGGCCGCCATGGCGGCCGCGGGAAT
TCGATTCCGACTCGAGGCGCATGTGTGGTACAACAGGTTCAGCCAGTGCT
GTTAGCCCTCGCTACGTTCTCGCGTAGCGATAAAGCATTTAAAGCACTAC
GCGGGAGCCCAAGACACCTCTACGGTGGTCCTAAATAGACGGTGTAGCAC
AGTAGATCGTCTAAATTATTTCACATCTTTACGTTACTATGAACTCTGTC
TTTTACAATCCGTTTGGTCGAGGTGCCTACGCTCAACCTCCAATAGCATG
GAGGCCAAGACGTAGGCGTGCACCTGCGCCTCGACCATCCGGGTTGACTA
CCCAGATCCAACAGCTCACTAGGGCTGTTGGAGCTTTGGTGCTGGACAAT
GCTACACGTCGCCAGCGCCCGGCTCCTCGCACGGCGCCGAGGAAGCCGAA
GACTCAAAAACCTAAGCCGAAGAAGCCAAACCGGAAACCACCACAATCAG
TCCTCGAGTCGGAATCACTAGTGAATTCGCGGCCGCCTGCAGGTCGACCA
TATGGGAGAGCTCCCAACGCGTTGGATGCATAGCTTGAGTATTCTATAGT
GTCACCTAAATAGCTTGGCGTAATCATGGTCATAGCTGTTTCCTGTGTGA
AATTGTTATCCGCTCACAATTCCACACAACATACGAGCCGGAAGCATAAA
GTGTAAAGCCTGGGGTGCCTAATGAGTGAGCTAACTCACATTAATTGCGT
TGCGCTCACTGCCCGCTTTCCAGTCGGGAAACCTGTCGTGCCAGCTGCAT
TAATGAATCGGCCAACGCGCGGGGAGAGGCGGTTTGCGTATTGGGCGCTC
TTCCGCTTCCTCGCTCACTGACTCGCTGCGCTCGGTCGTTCGGCTGCGGC
GAGCGGTATCAGCTCACTCAAAGGCGGTAATACGGTTATCCACAGAATCA
GGGGATAACGCAGGAAAGAACATGTGAGCAAAAGGCCAGCAAAAGGCCAG
GAACCGTAAAAAGGCCGCGTTGCTGGCGTTTTTCCATAGGCTCCGCCCCC
CTGACGAGCATCACAAAAATCGACGCTCAAGTCAGAGGTGGCGAAACCCG
ACAGGACTATAAAGATACCAAGGCGTTTCCCCCTGGAAACTCCCTCGTGC
GCTCTCCTGTTCCGACCCTGCCGCTTACCGGATACTTGTCCGCCTTTCTC
CCTTCTGGGAGACGGGGGGCTTTTCCTAGCTCACGCCTGGAGGGATCCCA
ATTCCGGGGGAGGGGGGTTCCCTCCAAACTTGGGGGTGTGGGGCAAAAAC
CCCCCGTTTAAGCCGAACGGTGGGCCTTTTTCTGGGAATATATTTTTTTG
GGGTCAACCCCGGGAAAGACAGATTATTTTCCTTTGGGGGGCGGCCCGGG
CAAGGGAAATAAAAAAAGAAGAAAATAAAAGGGGGGGTGCGAGGGTTCTT
TTAAGGGGGGGGGCCACCCCGGCCCCCCCAAAAAAACAAAAAAAAAAATA
TGTTTCCTTGGGGGGGGCTCCCTCCCGCAAAAGAAGGGGGTTTTTCGGGG
AAAAACCCCCCCCCACGGGGCGGGTGTGTGTGGTGGGGTTTTTAAAAAAA
AAAAAAAAAAGAAAAAAGTAAAAAAATTGGGGGGGGCCGCGGGGGAGGGCG

>MP31
GGATGGCGAGGTCCCATGGCTCCGGCCGCCATGGCGGCCGCGGGAATTCG
ATTCCGACTCGAGGATGAGGTGTGGTGGTTTCCGGTTTTGCTTCTTCGGC
TTAGGTTTTTGAGTCTTCGGCTTCCTCGGCGCCGTGCGAGGAGCCGGGCG
CTGGCGACGTGTAGCATTGTCCAGCACCAAAGCTCCAACAGCCCTAGTGA
GCTGTTGGATCTGGGTAGTCAACCCGGATGGTCGAGGCGCAGGTGCACGC
CTACGTCTTGGCCTCCATGCTATTGGAGGTTGAGCGTAGGCACCTCGACC
AAACGGATTGTAAAAGACAGAGTTCATAGTAACGTAAAGATGTGAAATAA
TTTAGACGATCTACTGTGCTACACCGTCTATTTAGGACCACCGTAGAGGT
GTCTTGGGCTCCCGCGTAGTGCTTTAAATGCTTTATCGCTACGCGAGAAC
GTAGCGAGGGCTAACAGCACTGGCTGAACCTCTTGTACCACACCTTCACC
TCGAGTCGGAATCACTAGTGAATTCGCGGCCGCCTGCAGGTCGACCATAT
GGGAGAGCTCCCAACGCGTTGGATGCATAGCTTGAGTATTCTATAGTGTC
ACCTAAATAGCTTGGCGTAATCATGGTCATAGCTGTTTCCTGTGTGAAAT
TGTTATCCGCTCACAATTCCACACAACATACGAGCCGGAAGCATAAAGTG
TAAAGCCTGGGGTGCCTAATGAGTGAGCTAACTCACATTAATTGCGTTGC
GCTCACTGCCCGCTTTCCAGTCGGGAAACCTGTCGTGCCAGCTGCATTAA
TGAATCGGCCAACGCGCGGGGAGAGGCGGTTTGCGTATTGGGCGCTCTTC
CGCTTCCTCGCTCACTGACTCGCTGCGCTCGGTCGTTCGGCTGCGGCGAG
CGGTATCAGCTCACTCAAAGGCGGTAATACGGTTATCCACAGAATCAGGG
GATAACGCAGGAAAGAACATGTGAGCAAAAGGCCAGCAAAAGGCCAGGAA
CCGTAAAAAGGCCGCGTTGCTGGCGTTTTTCCATAGGCTCCGCCCCCCTG
ACGAGCATCACAAAAATCGACGCTCAAGTCAGAGGTGGCGAAACCCGACA

GGACTATAAAGATACCAGGCGTTTCCCCCTGGAAGCTCCCTCGTGGGCTC
TCCTGTTCCGACCCTGCCGCTTACCGGATACCTGTCCGCTTTCTCCCTTC
GGGAAAGTGGGGCTTTTCCAAAGTCACCCCGGAGGGATTCCAATTCCGGG
GGAAGGGGTTCCCTCCAACTGGGGTTGGGTGCGAAACCCCCCGTCACCCC
AACGGTTGGCCTTATCTGGGAAATTATGCTTTTAGTCCACCCCGGGAAGA
ACAATTATTTCCCTTTGGGGGGGCCCCTGGGAAAGGGAAAAAAAAAAGAG
GGGTTAGGGGGGGGGGCGATTTTTTTATATGGGGGGGGCCCCCCGCCCCC
AAAAAAAAAATAAATTTTTTCTTTGTGGGCGCCCTCCTCCAAAAAAAAGG
GTTTGGTGTTAAAAACAACAAACCCGC

>MP33
GGGGGAGCGAGTCGCTGCTCCGGCCGCCTGGCGGCCGCGGGAATTCGATT
CCGACTCGAGGTGCCCGTGTGGCAAAGATAGTGAACGAGAAAGGCATCTC
CTCGGTAGCTATACCGCTGCTCTCAACAAGTATCTATGCTGCCGGAGCTG
ATCGCCTGGATTTCTCGCTGAGATGTCTTTTTACCGCGCTGGATCGTACG
GATGCGGATGTCACAATATATTGCCTAGATAAGAAGTGGGAGCAACGCAT
AGCAGATGCTATTAGGATGCGAGAACACGTAACTGAATTAAAAGATCCGG
ACATAGAGATAGATGAAGGATTAACCCGGGTACACCCAGATAGCTGCCTC
AAGGATCACATAGGCTACAGTACCAGTATGGGGAAATTGTACTCATACTT
TGAAGGTACTAAATTCCACCGAACCGCAAAAGACATAGCCGAGATTCGTG
CGCTGTTTCCTGATGTACAAGCCGCTAACGAACAAATCTGCCTGTACACT
TTAGGCGAACCGATGGAGTCCATACGCGAAAAGTGCCCAGTCGAAGACTC
CCCGGCATCAGCACCTCCTAAGACAATACCTTGCCTATGTATGTATGCTA
TGACAGCCGAACGTATTTGCCGGCTACGCAGTAACTCCGTAACGAACATA
ACGGTGTGCTCATCCTTTCCGTTACCCAAGTACCGAATAAAGAACGTACA
AAAGGTACAGTGCACGAAAGTTGTGCTATTTAACCCGGATGTACCGCCCT
ACATTCCCGCGAGAGTATACATTAACAAGGGACGAGCCCTCCTGTTACTC
CCCCACACCACGGGCCTCCAAGTCGGGAATCACCTAGGGGAAATTCCCGG
GCCGCCTTGCAGGGTCGACCCATATGGGGAGAAGCTCCCCAACCGCGTTG
GAATGCATAACCTTGGAGAATTCCTATAAGGGGCCCCCTAAAAAAGCTTG
GGCGGAAATCCTGGGGCCAAAAGCGTGTTTCCCGGGGGGGAAAATTGGTT
ATCCCGCTCCAAAATTTCCCACCAAAAAAAAACGAAGCCCGGGAAAGCAT
AAAAAGGTGAAAAACCCCGGGGGGGGGCCCAAAAAGAGAGGGAGCCAAAA
CCCCCCAAATTAAATTGGGGGGTGGGGCCCTCCATTGGCCCCGGCTTTTT
CCAAGTGGGGGGGAAAAACCTTGTGTTGTGGGCCCCCCCGGGCTTTATTA
TAAGGAAAACCGGGCCCAAACCCCCCCCGGGGGGGAAAAAAAGGGGGGGG
TTTTTTTGCCTTTTT

>MP35
GGGAGGGCAAGGCGCATGCTCTCGGCCGCCTGGCGGCCGCGGGAATTCGA
TTCCGACTCGAGGAAGCAGTGTGGTTCAGAGTGGGGATCCAAGGAGAACT
TTTGAAGGCCGTCGAGTCACGCTATGAGGTACAAGAGGTTCAGCCAGTGC
TGTTAGCCCTCGCTACGTTCTCGCGTAGCGATAAAGCATTTAAAGCACTA
CGCGGGAGCCCAAGACACCTCTACGGTGGTCCTAAATAGACGGTGTAGCA
CAGTAGATCGTCTAAATTATTTCACATCTTTACGTTACTATGAACTCTGT
CTTTTACAATCCGTTTGGTCGAGGTGCCTACGCTCAACCTCCAATAGCAT
GGAGGCCAAGACGTAGGCGTGCACCTGCGCCTCGACCATCCGGGTTGACT
ACCCAGATCCAACAGCTCACTAGGGCTGTTGGAGCTTTGGTGCTGGACAA
TGCTACACGTCGCCAGCGCCCGGCTCCTCGCACGGCGCCGAGGAAGCCGA
AGACTCAAAAACCTAAGCCGAAGAAGCAAAACCAGAAACCACCACAACTC
CCCCTCGAGTCGGAATCACTAGTGAATTCGCGGCCGCCTGCAGGTCGACC
ATATGGGAGAGCTCCCAACGCGTTGGATGCATAGCTTGAGTATTCTATAG
TGTCACCTAAATAGCTTGGCGTAATCATGGTCATAGCTGTTTCCTGTGTG
AAATTGTTATCCGCTCACAATTCCACACAACATACGAGCCGGAAGCATAA
AGTGTAAAGCCTGGGGTGCCTAATGAGTGAGCTAACTCACATTAATTGCG
TTGCGCTCACTGCCCGCTTTCCAGTCGGGAAACCTGTCGTGCCAGCTGCA
TTAATGAATCGGCCAACGCGCGGGGAGAGGCGGTTTGCGTATTGGGCGCT
CTTCCGCTTCCTCGCTCACTGACTCGCTGCGCTCGGTCGTTCGGCTGCGG
CGAGCGGTATCAGCTCACTCAAAGGCGGTAATACGGTTATCCACAGAATC
AGGGGATAACGCAGGAAAGAACATGTGAGCAAAAGGCCAGCAAAAGGCCA

GGAACCGTAAAAAGGCCGCGTTGCTGGCGTTTTTCCATAGGCTCCGCCCC
CCTGACGAGCATCACAAAAATCGACGCTCAAGTCAAAAGGTGGCGAAACC
CGACAGGACTATAAAGAAACCAAGCGTTTCCCCCTGGAAGCTCCCTCGGG
GGGTCTCCTGGTCCAACCCTGCGGGTTACGGGAAACTGTGCGGCTTTCTC
CCCTTCGGGAGAGGGGGGGCGTTTCCTAAACTCCCCCCTGAGGGAATCAC
AAACCCGGGGGAGGGGGTCCCTCCAACCGGGGCGGTGGTGGGAACACCCC
CGTAACCCAGACGGGCGCGCTTTCCTGGGAATAATTTTT

>MP37
GGACAGGCTAGGTCGCATGGCCTCCGGCCGCCATGGCGGCCGCGGGAATT
CGATTCCGACTCGAGGGGGCACTGTGGTACAAGAGGTTCAGCCAGTGCTG
TTAGCCCTCGCTACGTTCTCGCGTAGCGATAAAGCATTTAAAGCACTACG
CGGGAGCCCAAGACACCTCTACGGTGGTCCTAAATAGACGGTGTAGCACA
GTAGATCGTCTAAATTATTTCACATCTTTACGTTACTATGAACTCTGTCT
TTTACAATCCGTTTGGTCGAGGTGCCTACGCTCAACCTCCAATAGCATGG
AGGCCAAGACGTAGGCGTGCACCTGCGCCTCGACCATCCGGGTTGACTAC
CCAGATCCAACAGCTCACTAGGGCTGTTGGAGCTTTGGTGCTGGACAATG
CTACACGTCGCCAGCGCCCGGCTCCTCGCACGGCGCCGAGGAAGCCGAAG
ACTCAAAAACCTAAGCCGAAGAAGCAAAACCGGAAACCACCACATCCGCC
CCTCGAGTCGGAATCACTAGTGAATTCGCGGCCGCCTGCAGGTCGACCAT
ATGGGAGAGCTCCCAACGCGTTGGATGCATAGCTTGAGTATTCTATAGTG
TCACCTAAATAGCTTGGCGTAATCATGGTCATAGCTGTTTCCTGTGTGAA
ATTGTTATCCGCTCACAATTCCACACAACATACGAGCCGGAAGCATAAAG
TGTAAAGCCTGGGGTGCCTAATGAGTGAGCTAACTCACATTAATTGCGTT
GCGCTCACTGCCCGCTTTCCAGTCGGGAAACCTGTCGTGCCAGCTGCATT
AATGAATCGGCCAACGCGCGGGGAGAGGCGGTTTGCGTATTGGGCGCTCT
TCCGCTTCCTCGCTCACTGACTCGCTGCGCTCGGTCGTTCGGCTGCGGCG
AGCGGTATCAGCTCACTCAAAGGCGGTAATACGGTTATCCACAGAATCAG
GGGATAACGCAGGAAAGAACATGTGAGCAAAAGGCCAGCAAAAGGCCAGG
AACCGTAAAAAGGCCGCGTTGCTGGCGTTTTTCCTTAGGCTCCGCCCCCC
TGACGAGCATCACAAAAATCGACGCTCAAGTCAGAGGTGGCGAAACCCGA
CAGGACTTTAAAGATACCAGGCGTTTCCCCCTGGAAGCTCCCTCCGGGGG
CTTCCTTGTTCCGACCCTGCCGCTTACCGGAAACCTGGCCGCCTTTCTCC
CCTTCGGAAACGGGGGGCTTTCCCAAAGCTAACCCTGGAGGATTCCAATT
CCGGGTGAGGGCGTTCGCTCCCAACTGGGGGTTGTGGGCCAAAACCCCCC
GTTCAACCCCGACCGGTGGCCCTTTTCCCGGGAAATTTCGCTCTTGGTTC
CAACCCGGGGAAAACACAATTTTACCCCCCTTGGGGCGACCCCCCTGGGG
ACAAGGTTTTAAAAAAAAAAAGGATTATAAAGGG
